# Supplementary material for: Dual Mechanisms of LYN Kinase Dysregulation Drive Aggressive Behavior in Breast Cancer Cells
Source: Cell Rep. 2018 Dec 26;25(13):3674–3692.e10. doi: 10.1016/j.celrep.2018.11.103 (PMC6315108; doi:10.1016/j.celrep.2018.11.103)
Supplement: Document S1. Figures S1–S10 [file mmc1.pdf]

Cell Reports, Volume *25*

Supplemental Information

Dual Mechanisms of LYN Kinase Dysregulation

Drive Aggressive Behavior in Breast Cancer Cells

Giusy Tornillo, Catherine Knowlson, Howard Kendrick, Joe Cooke, Hasan Mirza, Iskander Aurrekoetxea-Rodríguez, Maria d.M. Vivanco, Niamh E. Buckley, Anita Grigoriadis, and Matthew J. Smalley

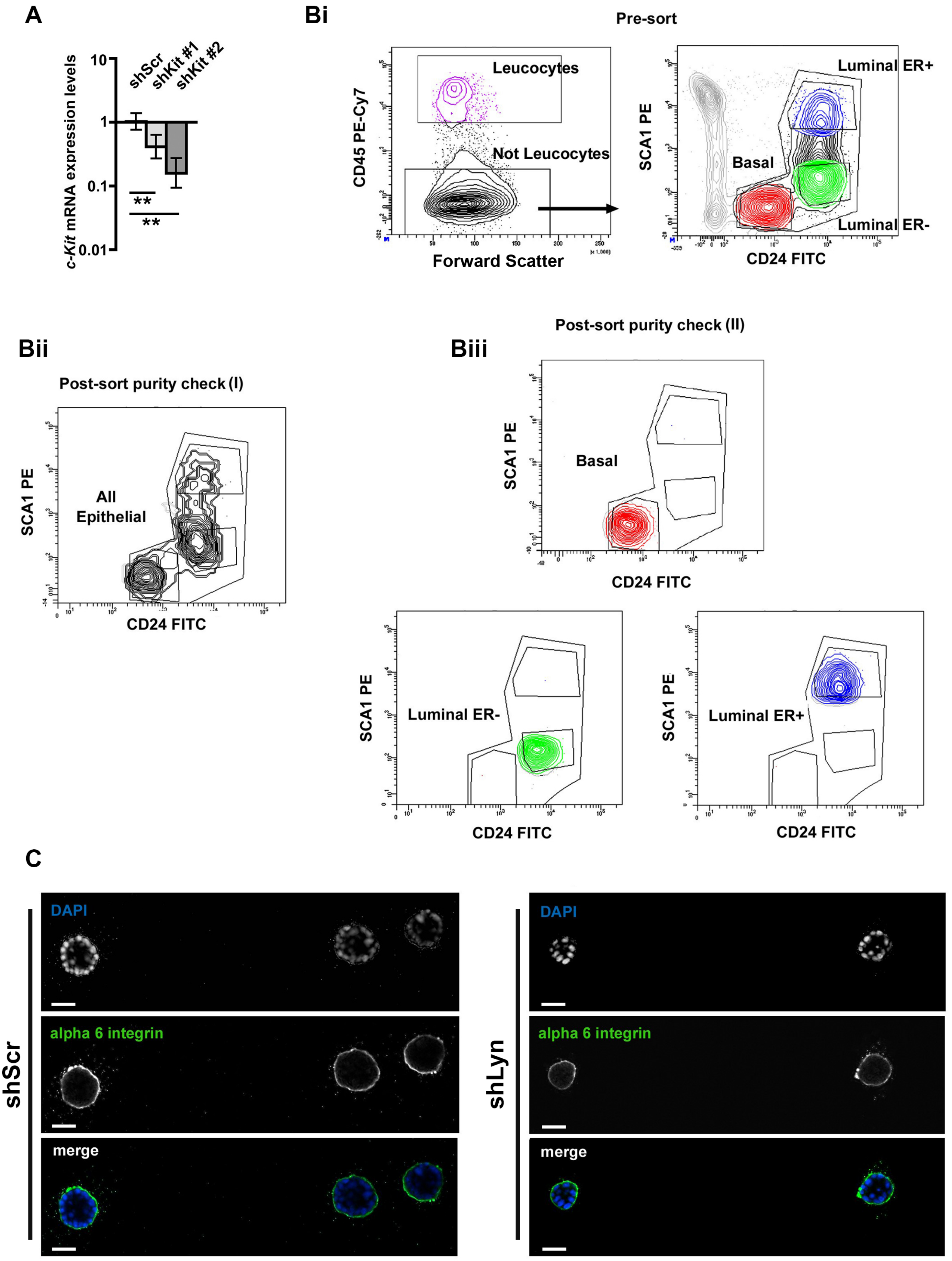

A
c-Kit mRNA expression levels
10
1
0.1
0.01
shScr
shKit #1
shKit #2
**
**
Bi
Pre-sort
CD45 PE-Cy7
Leucocytes
Not Leucocytes
Forward Scatter
SCA1 PE
Luminal ER+
Basal
Luminal ER-
CD24 FITC
Bii
Post-sort purity check (I)
SCA1 PE
All Epithelial
CD24 FITC
Biii
Post-sort purity check (II)
SCA1 PE
Basal
CD24 FITC
SCA1 PE
Luminal ER-
CD24 FITC
SCA1 PE
Luminal ER+
CD24 FITC
C
shScr
DAPI
alpha 6 integrin
merge
shLyn
DAPI
alpha 6 integrin
merge

Figure S1. *c-Kit* expression following shKit transduction, isolation of Luminal ER- progenitor cells and assessment of polarity in MCF10A acini (related to Figure 1 and Figure 2). (A) Analysis of *c-Kit* expression by qrtPCR in primary mouse mammary organoids after transduction with control (shScr) or c-Kit-knock-down (shKit#1 and shKit#2) lentiviruses (mean±95% confidence intervals; significance of real time rtPCR data determined from confidence intervals; n=3 independent experiments each of 3 technical replicates per sample) (Cumming et al., 2007). **P<0.01 **(B) (i)** Flow cytometry of primary mammary cells stained with antibodies against CD45, CD24 and Sca-1. $CD45^+$ leucocytes (purple) were gated out (left plot) and the $CD45^-$ cells (right plot) gated to fractionate All Epithelial cells or Basal ($CD24^{+/low}$ Sca-1^-, red), Luminal ER- ($CD24^{+/high}$ Sca-1^-, green), and Luminal ER+ ($CD24^{+/high}$ Sca-1^+, blue) epithelial cell subpopulations as we have previously described (Kendrick et al., 2008; Regan et al., 2012; Sleeman et al., 2007). Post-sort purity checks for total epithelial cells **(ii)** and sorted sub-populations **(iii)** also shown. **(C)** Polarity of shScr-, shLyn#1- or shLyn#2-transduced MCF10A cells in 3D culture (day 12) determined by immunofluorescence analysis of alpha6 integrin expression. Scale bar, 50 μm.

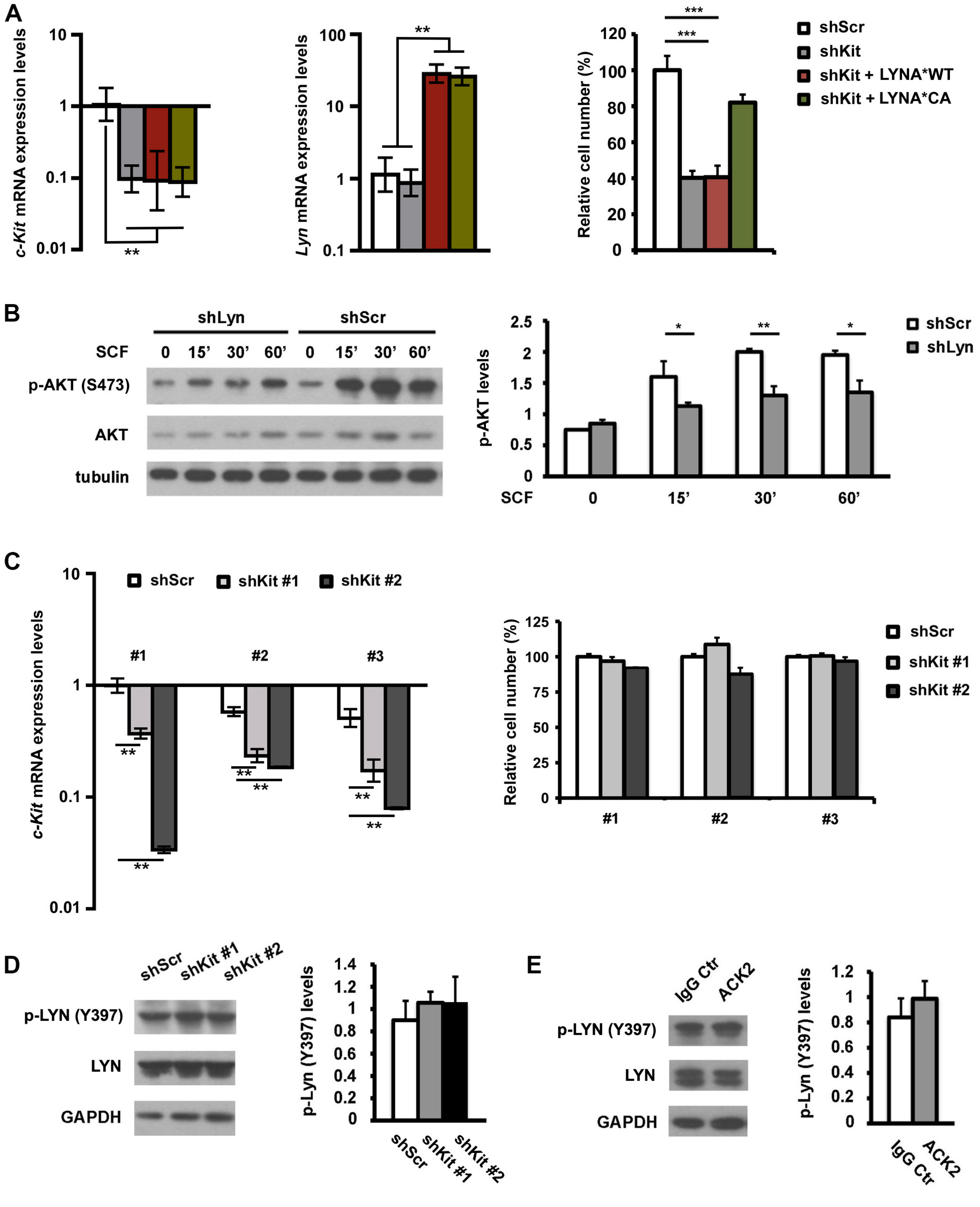
A
c-Kit mRNA expression levels
Lyn mRNA expression levels
Relative cell number (%)
shScr
shKit
shKit + LYNA*WT
shKit + LYNA*CA
B
shLyn
shScr
SCF
0 15' 30' 60' 0 15' 30' 60'
p-AKT (S473)
AKT
tubulin
p-AKT levels
shScr
shLyn
C
shScr
shKit #1
shKit #2
c-Kit mRNA expression levels
#1
#2
#3
Relative cell number (%)
D
shScr
shKit #1
shKit #2
p-LYN (Y397)
LYN
GAPDH
p-Lyn (Y397) levels
E
IgG Ctr
ACK2
p-LYN (Y397)
LYN
GAPDH
p-Lyn (Y397) levels

Figure S2. c-KIT promotes proliferation and survival of normal mammary epithelial cells but not BRCA1 tumor cells (related to Figure 2). **(A)** Mammary organoids were transduced with shScr and empty expression vectors, shKit and empty expression-vectors, or shKit and expression vectors carrying either wild-type LYNA (LYNA WT) or a constitutively active LYNA mutant (LYNA CA). *c-Kit* and *Lyn* expression levels were determined by qrtPCR 5 days after transduction (left and middle panels). Bar graph (right) shows cell number at day 5 of culture relative to shScr cells. **(B)** Western blot analysis (left) of AKT phosphorylation (S473) and total AKT levels in protein extracts from primary mouse mammary organoids transduced with either control (shScr) or *Lyn* knockdown (shLyn) lentiviruses, either unstimulated (0') or stimulated with SCF for 15, 30 or 60 minutes. Tubulin used as loading control. Right, quantification of phospho-AKT levels (normalized to total AKT). **(C)** *Brca1*-tumor derived cell lines (#1, #2, #3) were transduced with shScr control or two c-KIT knockdown lentivirus (shKit#1 and t#2). Left panel, qrtPCR analysis of *c-Kit* expression relative to shScr cells. Right panel, relative cell number of shScr, shKit#1 and #2 cells. **(D, E)** LYN autophosphorylation (Y397) in protein extracts from *Brca1*-tumor derived cells after transduction with control (shScr) or c-Kit-knockdown (shKit#1 and #2) lentivirus **(D)** or treatment with c-KIT blocking (ACK2) or IgG isotype (IgG Ctr) antibodies **(E)**. Blots are representative of three independent experiments. Quantitation shown as mean and SD (n=3 biological replicates; two-tailed unpaired t-tests), except for gene expression analysis by quantitative real time rtPCR (mean±95% confidence intervals; significance of real time rtPCR data determined from confidence intervals; n=3 independent experiments each of 3 technical replicates per sample) (Cumming et al., 2007). *P<0.05; **P<0.01; ***P<0.001.

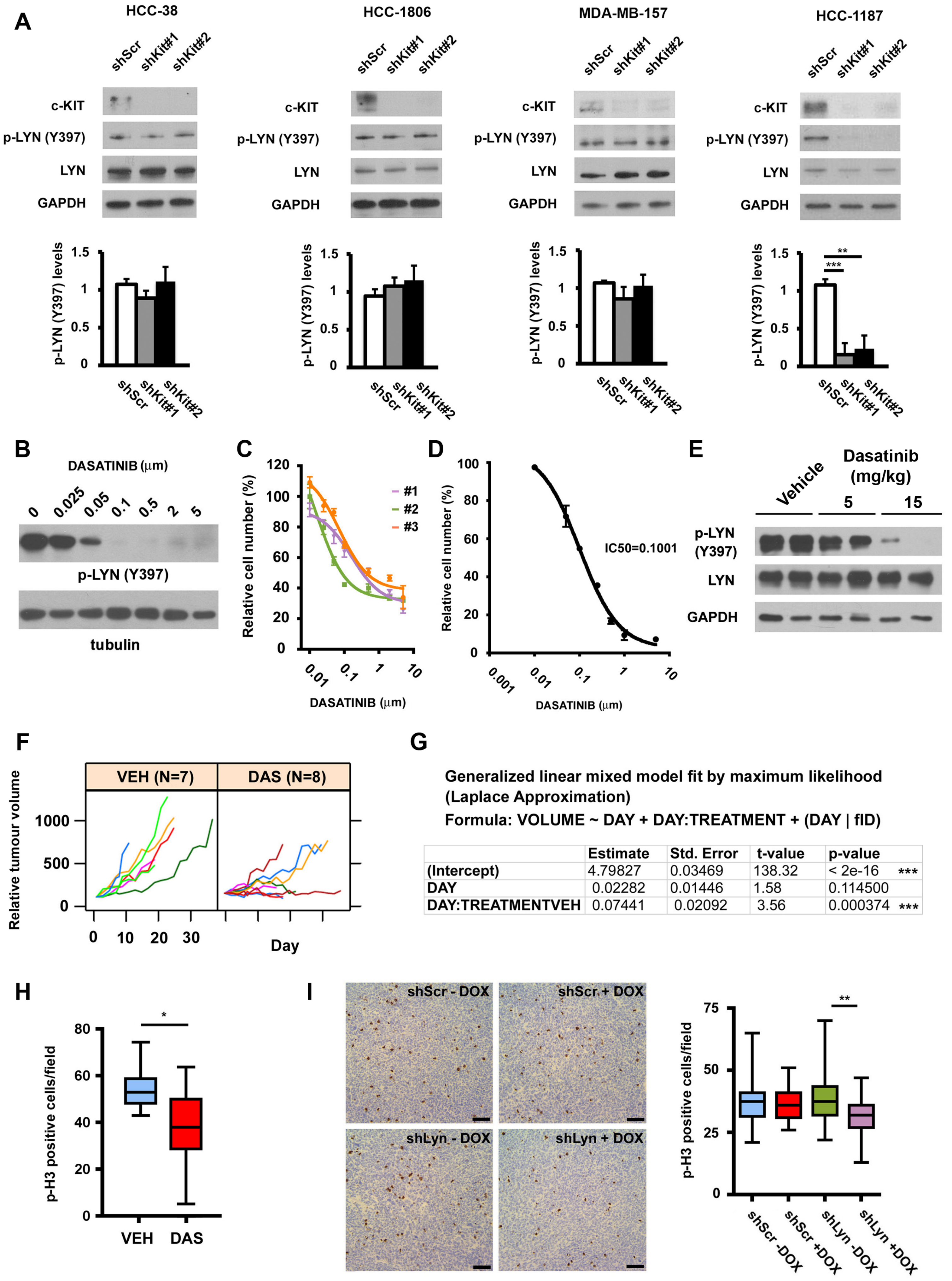

	Estimate	Std. Error	t-value	p-value
(Intercept)	4.79827	0.03469	138.32	< 2e-16 ***
DAY	0.02282	0.01446	1.58	0.114500
DAY:TREATMENTVEH	0.07441	0.02092	3.56	0.000374 ***

Figure S3. LYN phosphorylation is not affected by c-KIT knockdown in BRCA1 defective human breast cancer cell lines and Dasatinib both inhibits the growth of BRCA1 defective cell lines and blocks BRCA1 tumour growth *in vivo* (related to Figure 2 and Figure 3). (A) Representative western blot analyses and quantitation from three independent experiments of LYN autophosphorylation (Y397) following knockdown of c-KIT with two independent shRNAs in four c-KIT+ human breast cancer cell lines, HCC-38 (*BRCA1* silenced by methylation), HCC-1806 and MDA-MB-157 (BRCA1 low due to miRNA expression) and HCC-1187 (BRCA1 wt). **(B)** Western blot analysis of LYN autophosphorylation (Y397) in mammary organoids following treatment with vehicle or Dasatinib at concentrations from 0.025 to 5 μM. Tubulin used as loading control. **(C)** Primary *BlgCre Brca1*$^{fl/fl}$ *p53*$^{+/-}$ mouse mammary tumour cells (#1, #2 and #3) were seeded on Matrigel and treated with vehicle or the indicated Dasatinib concentrations for 5 days. Cell number assessed by CellTitreGlo assay relative to vehicle-only treated cells. Graph show dose response curves using a Nonlinear Regression (Curve Fit) model. **(D)** HCC1937 cells were treated with vehicle or the indicated Dasatinib concentrations stained with crystal violet after 7 days. Viable cell density was determined as in Figure 4A. Graph shows dose response curve and IC50 derived from nonlinear curve fitting (right panel). Blots are representative of three independent experiments. Unless otherwise stated, quantitation shown as mean and SD from three independent experiments and statistical significance determined using two-tailed unpaired t-tests. *P<0.05; **P<0.01; ***P<0.001. **(E)** Wild-type mice were treated with daily intra-peritoneal injection (IP) of vehicle (N=2), Dasatinib - 5mg/Kg (N=2) or Dasatinib - 15mg/Kg (N=2) for 7 days. Mammary glands were harvested from each mouse 6 hours after last injection and lysed. Protein extracts were analyzed for levels of phosphorylated (Y397) and total LYN by western blot. GAPDH was used as loading control. **(F)** *Blg-Cre Brca1*$^{fl/fl}$*p53*$^{+/-}$ mice carrying mammary tumours were treated with daily IP injection of vehicle (VEH, N=7) or Dasatinib (DAS, 15mg/Kg, N=8). Tumour size was recorded every two days. Tumour volume was calculated and plotted as relative to volume at day 0 of treatment. **(G)** Statistical analysis of tumour growth in vehicle- and Dasatinib-treated *Blg-Cre Brca1*$^{fl/fl}$*p53*$^{+/-}$ mice using the *glmer* function for generalized linear mixed models from the *lme4* package in the R software. The summary of the final model (VOLUME ~ DAY + DAY:TREATMENT + (DAY|fTUMOUR_ID), family = gaussian (link = "log) is shown in the table. **(H)** Quantitation of phospho-histone3 (pH3) immunohistochemical staining of tumours derived from vehicle- or Dasatinib-treated *BlgCre Brca1*$^{fl/fl}$ *p53*$^{+/-}$ mice. Ten fields from each tumour were counted. **(I)** Phospho-Histone3 (pH3) immunohistochemical staining of tumours derived from transduced and transplanted *BlgCre Brca1*$^{fl/fl}$ *p53*$^{+/-}$ tumour cells (arrowheads indicate mitotic pH3-positive cells in insets) Scale bar = 100 um. Representative images and quantification of percentage of pH3-positive cells (box-plots) from ten fields each tumour (seven tumours per group). Blots are representative of three independent experiments. Quantitation shown as mean and SD from three independent experiments. Statistical significance was determined using two-tailed unpaired t-tests unless otherwise stated. *P<0.05; **P<0.01; ***P<0.001.

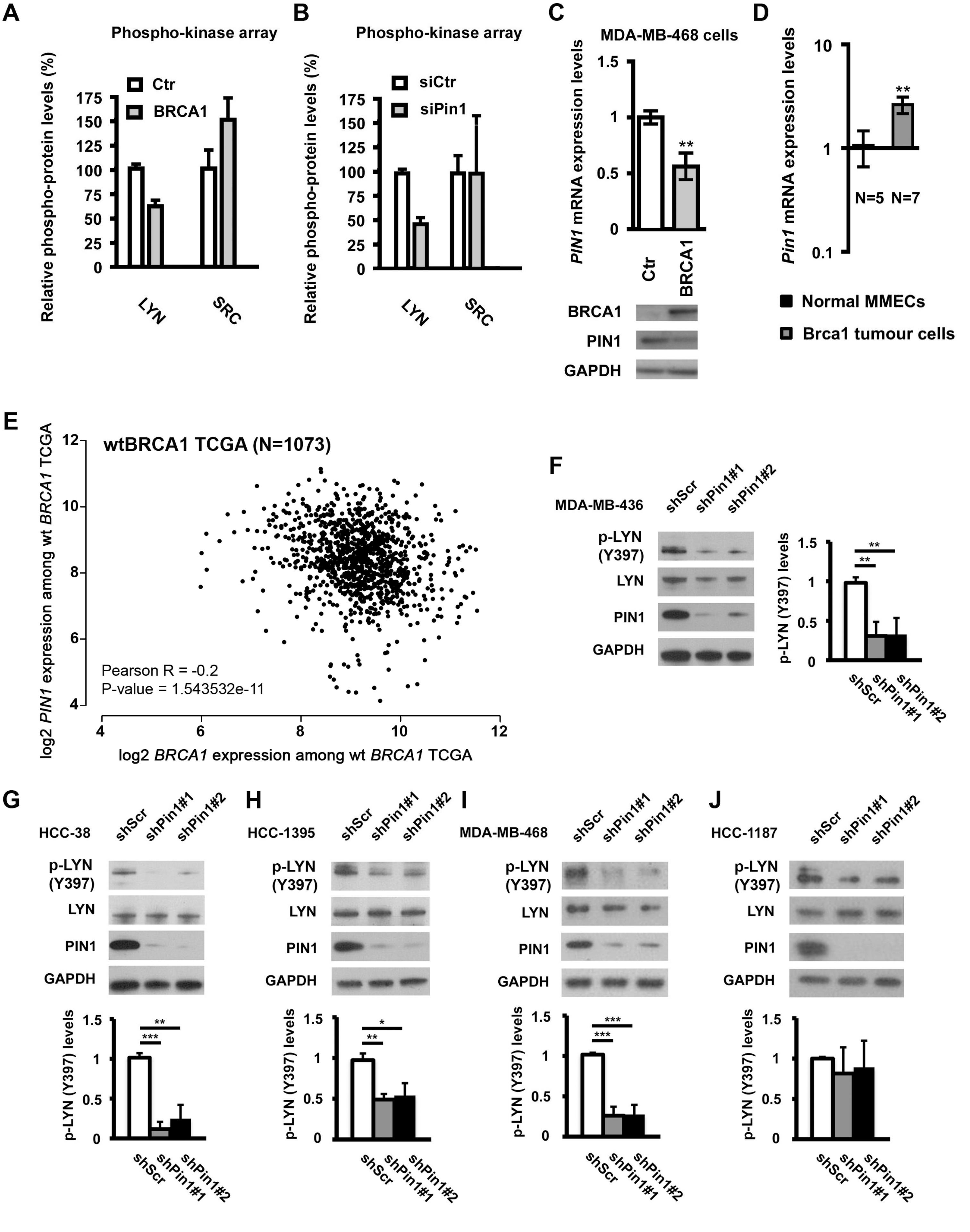

A
Phospho-kinase array
Ctr
BRCA1
Relative phospho-protein levels (%)
LYN
SRC
B
Phospho-kinase array
siCtr
siPin1
C
MDA-MB-468 cells
PIN1 mRNA expression levels
Ctr
BRCA1
BRCA1
PIN1
GAPDH
D
Pin1 mRNA expression levels
N=5
N=7
Normal MMECs
Brca1 tumour cells
E
wtBRCA1 TCGA (N=1073)
Pearson R = -0.2
P-value = 1.543532e-11
log2 PIN1 expression among wt BRCA1 TCGA
log2 BRCA1 expression among wt BRCA1 TCGA
F
MDA-MB-436
shScr
shPin1#1
shPin1#2
p-LYN (Y397)
LYN
PIN1
GAPDH
p-LYN (Y397) levels
G
HCC-38
H
HCC-1395
I
MDA-MB-468
J
HCC-1187

Figure S4: LYN activity is regulated by BRCA1 via the prolyl isomerase PIN1 (related to Figure 4). (A, B) Quantification of relative phosphorylation levels of LYN and SRC proteins in MDA-MB-468 cells stably transfected with empty vector (Ctr) or wild-type BRCA1 (BRCA1) **(A)** and MDA-MB-468 cells transiently transfected with scrambled (siCtr) or PIN1 siRNA (siPin1) **(B).** Phosphorylation levels were determined by using a phospho-kinase array (R&D) and densitometry-based quantification by ImageJ (mean±SEM). **(C)** Human MDA-MB-468 BRCA1-low cells were stably transfected with either an empty vector (Ctr) or a vector carrying wild-type BRCA1 (BRCA1). PIN1 expression levels were assessed by qrtPCR (top) and western blot (below). Blots were also probed for BRCA1 and GAPDH as a loading control. **(D)** *Pin1* mRNA expression levels in primary mouse mammary epithelial cells from wild-type mice (normal MMECs, N= 5 preparations) and primary *BlgCre Brca1*$^{fl/fl}$ *p53*$^{+/-}$ mouse tumour cells (N=7 tumours). **(E)** Correlation between BRCA1 and PIN1 expression levels in germline wildtype BRCA1 breast cancers. Log2 base expression levels for PIN1 and BRCA1 were downloaded from TCGA level 3, for 1,073 TCGA BRCA1 wild type breast cancers. Pearson correlation was performed between the two gene expression levels across all samples. P value and r value are shown above the plot. **(F – J)** Representative western blot analyses and quantitation from three independent experiments of LYN autophosphorylation (Y397) following knockdown of PIN1 with two independent shRNAs in five cell lines, (F) MDA-MB-436 (BRCA1 mutant), (G) HCC-38 (BRCA1 deficient), (H) HCC-1395 (BRCA1 mutant), (I) MDA-MB-468 (BRCA1 low) and (J) HCC-1187 (BRCA1 wild type) (mean+SD; unpaired two-tailed t-test). *P<0.05; **P<0.01; ***P<0.001.

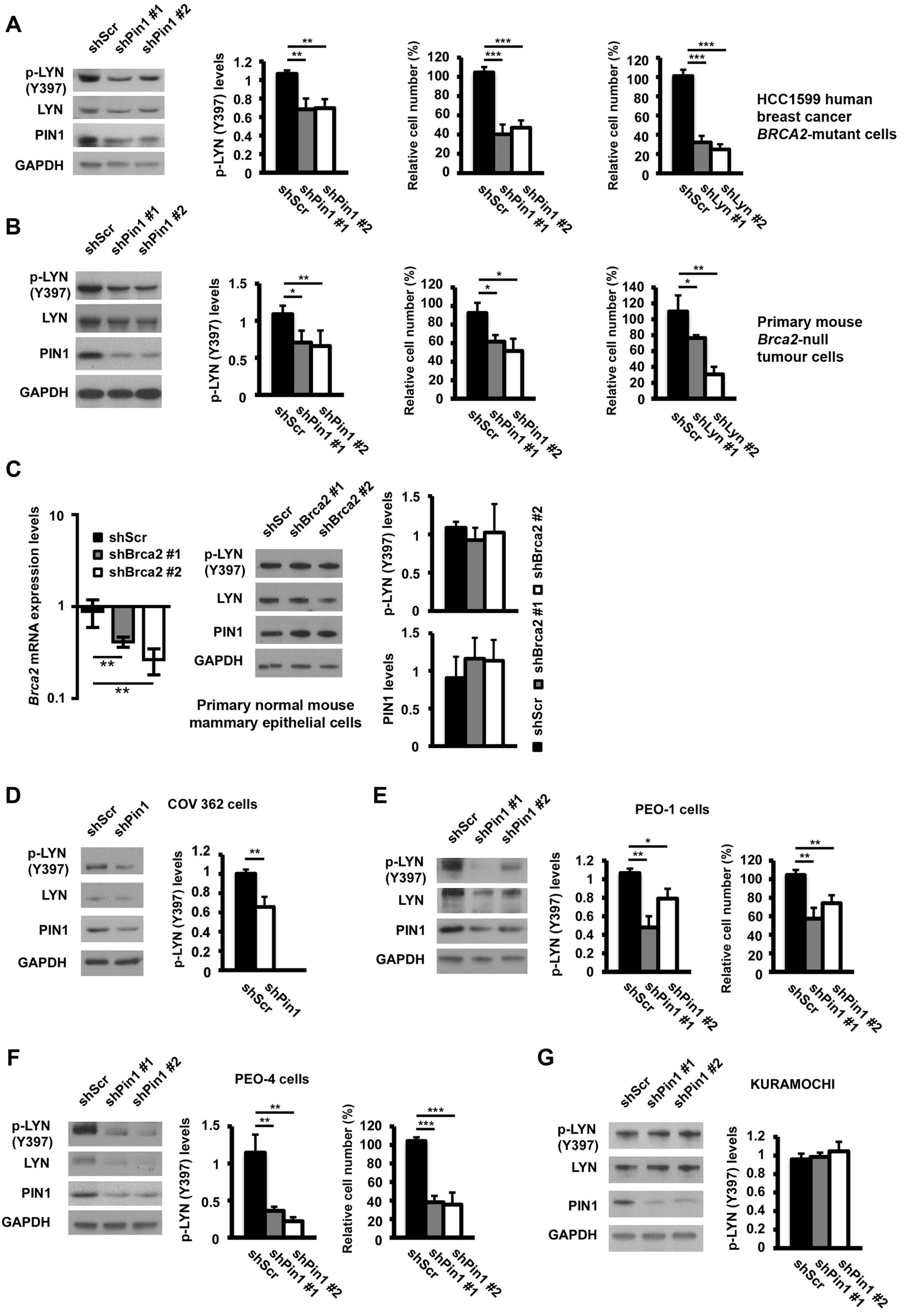
A
shScr shPin1 #1 shPin1 #2
p-LYN (Y397)
LYN
PIN1
GAPDH
p-LYN (Y397) levels
Relative cell number (%)
shScr shPin1 #1 shPin1 #2
shScr shLyn #1 shLyn #2
HCC1599 human breast cancer BRCA2-mutant cells
B
Primary mouse Brca2-null tumour cells
C
Brca2 mRNA expression levels
shScr
shBrca2 #1
shBrca2 #2
Primary normal mouse mammary epithelial cells
PIN1 levels
D
COV 362 cells
shScr shPin1
E
PEO-1 cells
F
PEO-4 cells
G
KURAMOCHI

Figure S5. PIN1 regulates LYN phosphorylation in BRCA2 null breast and ovarian cells, but BRCA2 does not regulate PIN1 (related to Figure 5).(A) Western blot analysis of LYN autophosphorylation levels in human HCC1599 BRCA2-deficient breast cancer cells transduced with either control (shScr) or or PIN1-knock-down lentiviruses (shPin1 #1 and shPin1 #2)(left). ShScr, shPin1 #1 and shPin1 #2 cells were also assayed for cell growth (middle). HCC1599 cells were also transduced with control (shScr) or Lyn-knock-down lentiviruses (shLyn#1 and shLyn#2) and tested for cell viability (right). **(B)** Primary cells isolated from three distinct *BlgCre Brca2*$^{fl/fl}$ *p53*$^{fl/fl}$ mouse mammary tumours were transduced with control (shScr) or *Pin1*-knock-down lentiviruses (shPin1 #1 and shPin1 #2) and assessed for p-LYN (Y397) levels by Western blot (left). ShScr, shPin1 #1 and shPin1 #2 *BlgCre Brca2*$^{fl/fl}$ *p53*$^{fl/fl}$ cells were seeded at low density in adherent conditions (2D) and stained with crystal violet after 6 days. Viable cell density was determined by absorbance measurement following solubilization of the dye (middle). *BlgCre Brca2*$^{fl/fl}$ *p53*$^{fl/fl}$ cells were also transduced with control (shScr) or Lyn-knock-down lentiviruses (shLyn#1 and shLyn#2) and tested for cell viability (right). **(C)** Primary mouse mammary organoids were transduced with control (shScr) or *Brca2* knock-down lentiviruses (shBrca2 #1 and shBrca2 #2). *Brca2* knock-down was assessed by qrtPCR (left; fold expression over comparator shScr cells). shScr and shBrca2 cells were assessed for levels of phospho-LYN (Y397), LYN, PIN1 and GAPDH by western blot after 4 days (middle panel, representative blot; right panel quantitation). **(D)** Western blot analysis of LYN autophosphorylation levels in human COV 362 BRCA1-deficient ovarian cancer cells transduced with either control (shScr) or PIN1-knock-down lentivirus (shPin1). **(E)** Analysis of LYN levels and cell viability in PEO1 human ovarian cancer cells after transduction with control (shScr) or PIN1-knock-down lentiviruses (shPin1 #1 and shPin1 #2). **(F)** Analysis of LYN levels and cell viability in PEO4 human ovarian cancer cells after transduction with control (shScr) or PIN1-knock-down lentiviruses (shPin1 #1 and shPin1 #2). **(G)** p-LYN (Y397) levels were measured in shScr, shPin1 #1 and shPin1 #2-transduced cells in KURAMOCHI human ovarian cancer cells. Blots are representative of three independent experiments. Quantitation shown as mean and SD from three independent experiments. Statistical significance was determined using two-tailed unpaired t-tests, except for gene expression analysis by quantitative real time rtPCR which is shown as mean±95% confidence intervals; significance of real time rtPCR data was determined from confidence intervals (n=3 independent experiments each of 3 technical replicates per sample) (Cumming et al., 2007). *P<0.05; **P<0.01; ***P<0.001.

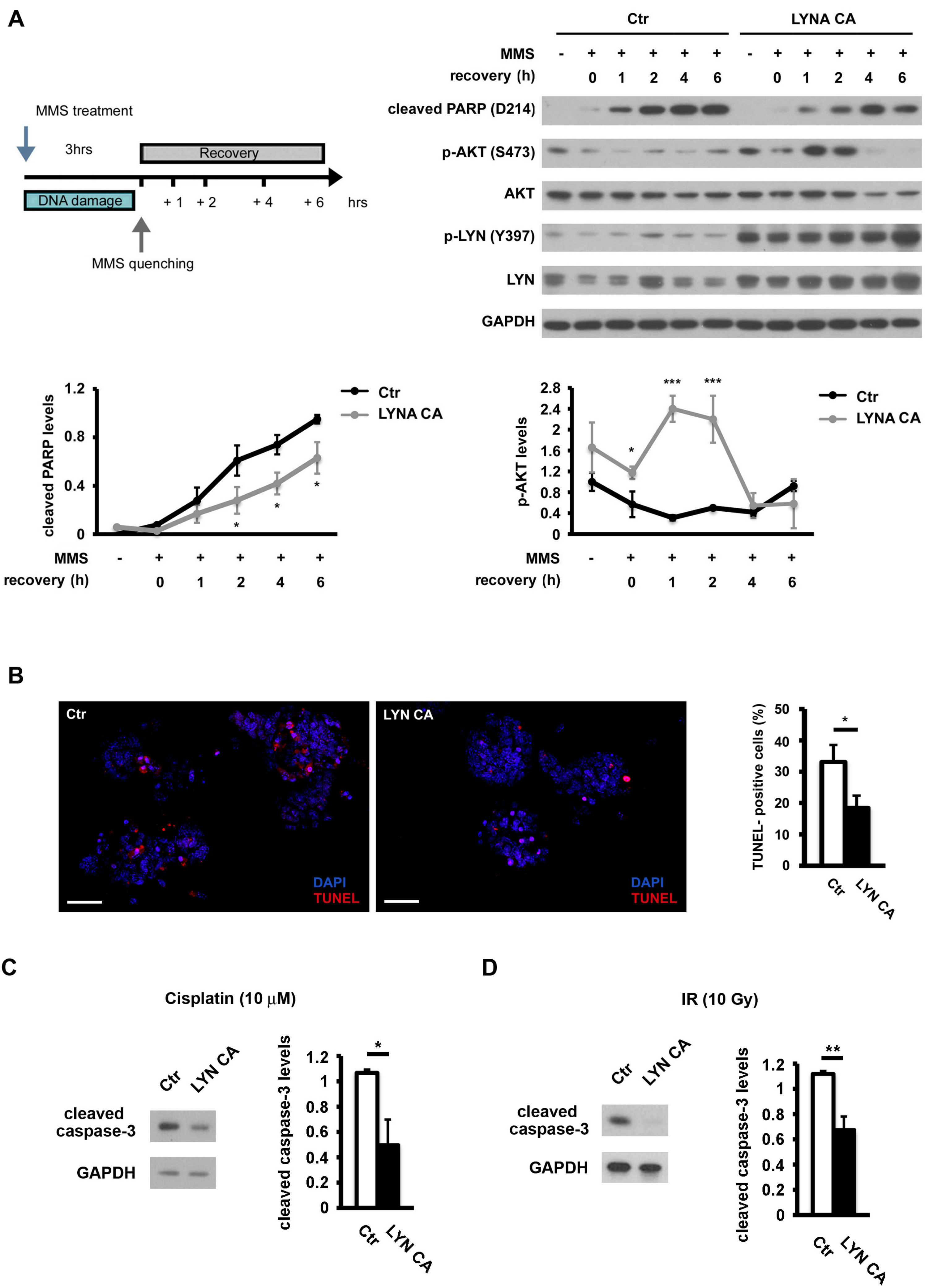
A
MMS treatment
3hrs
Recovery
DNA damage
+ 1
+ 2
+ 4
+ 6
hrs
MMS quenching
Ctr
LYNA CA
MMS
recovery (h)
cleaved PARP (D214)
p-AKT (S473)
AKT
p-LYN (Y397)
LYN
GAPDH
cleaved PARP levels
p-AKT levels
B
Ctr
LYN CA
DAPI
TUNEL
TUNEL- positive cells (%)
C
Cisplatin (10 μM)
cleaved caspase-3
cleaved caspase-3 levels
D
IR (10 Gy)

Figure S6. LYN protects normal mammary from apoptosis in response to DNA damage (related to Figure 5). (A) Response of primary mouse mammary organoids transduced with either an empty control (Ctr) lentiviral vector or a vector expressing a constitutively active mutant LYN (LYN CA) to Methyl Methane Sulfonate (MMS). 4 days after transduction both Ctr- and LYN CA-organoids were exposed to 100 uM MMS or left untreated. After 3 hours, cells were lysed or, following MMS quenching with sodium thiosulfate (2.5%), left to recover for 1, 2, 4 or 6 hours before lysis. Top left, schematics of the experimental conditions. Lysates were analyzed for phospho-LYN (Y397), LYN, cleaved PARP, phospho-AKT (S473), AKT and GAPDH by western blot (top right). Quantification of phospho-AKT and cleaved PARP levels, relative to AKT and GAPDH levels, respectively, are shown below. **(B)** Detection of i*n situ* apoptosis by TUNEL immunofluorescent staining in MMS-treated Ctr- or LYN CA-transduced organoids. 4 days after transduction organoids were treated with 1 uM MMS. After 2 hours, MMS was quenched and cells were left to recover for 24 hours before fixation. Left, representative images (bar=50 um). Right, quantitation. **(C, D)** Analysis and quantitation of cleaved caspase 3 levels in primary organoids transduced as in (B) and then either treated with Cisplatin (10 uM) for 48 hours before lysis (C) or exposed to ionising radiation (IR, 10 Gy) and left to recover for 24 hours before lysis (D). Blots are representative of three independent experiments. Unless otherwise stated, quantitation shown as mean and SEM from three independent experiments. Statistical significance was determined using two-tailed unpaired t-tests. *$P<0.05$; **$P<0.01$; ***$P<0.001$.

A LYN exons and isoforms (UCSC)

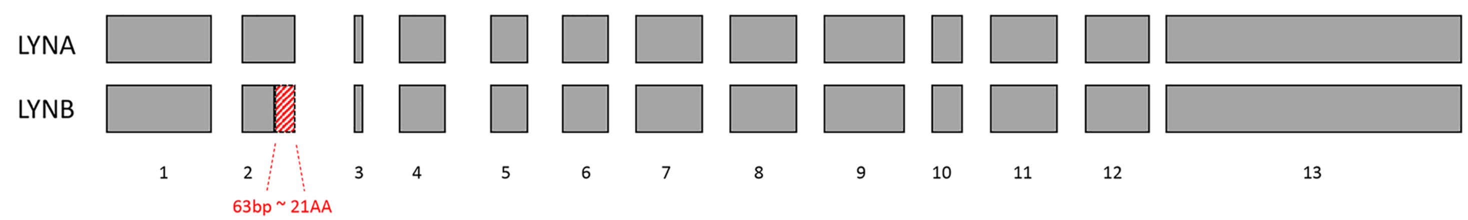

ExonNumber	exonStarts	exonEnds(uc003xsk.*, LYNA)	exonEnds(uc003xsl.*, LYNB)	ExonLength(LYNA)	ExonLength(LYNB)	DifferenceNT	DifferenceAA
Exon1	56792385	56792662	56792662	277	277	0	0
Exon2	56854413	56854550	56854487	137	74	63	21
Exon3	56859006	56859052	56859052	46	46	0	0
Exon4	56860176	56860282	56860282	106	106	0	0
Exon5	56863017	56863116	56863116	99	99	0	0
Exon6	56863239	56863343	56863343	104	104	0	0
Exon7	56864524	56864674	56864674	150	150	0	0
Exon8	56866390	56866543	56866543	153	153	0	0
Exon9	56879273	56879456	56879456	183	183	0	0
Exon10	56882275	56882352	56882352	77	77	0	0
Exon11	56910904	56911058	56911058	154	154	0	0
Exon12	56911976	56912108	56912108	132	132	0	0
Exon13	56922466	56925006	56925006	2540	2540	0	0

B

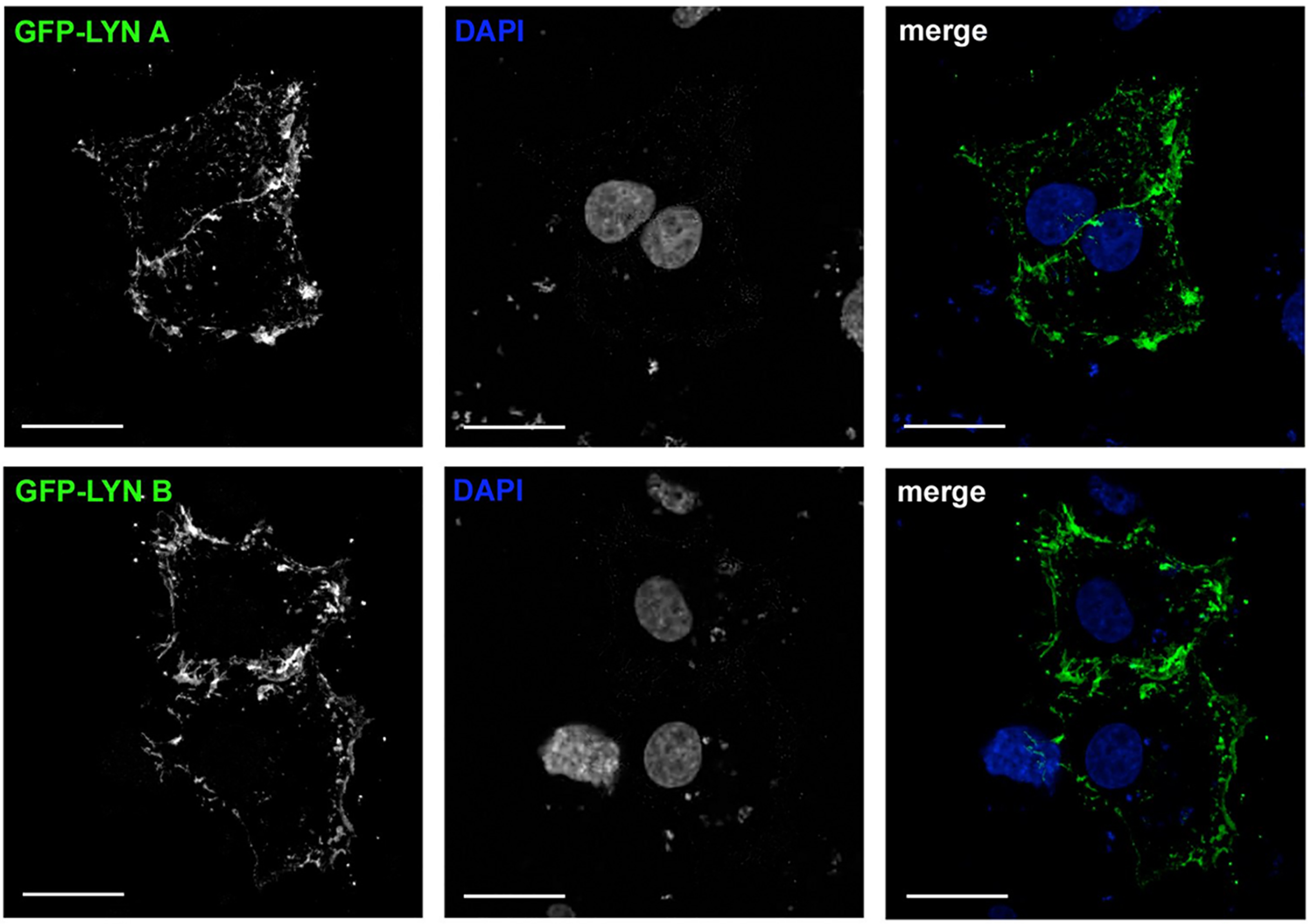

C

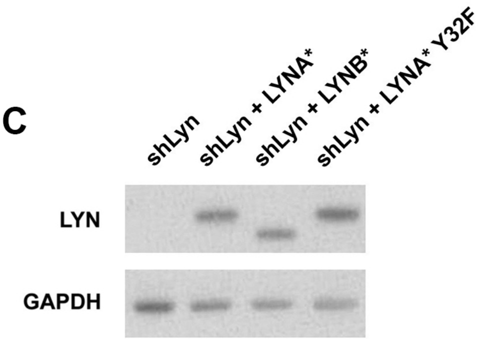

D

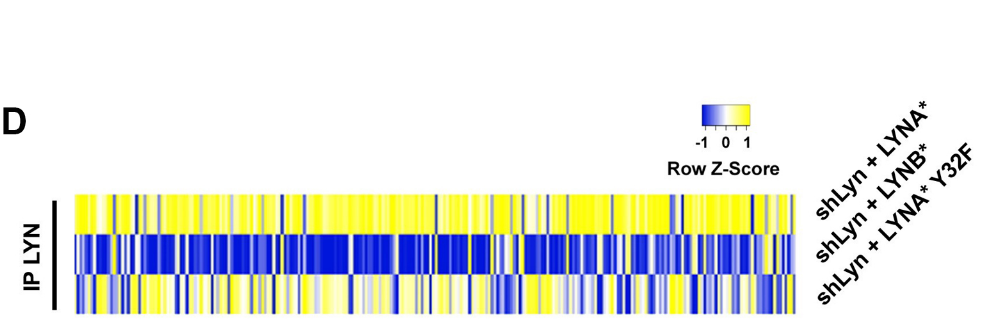

Figure S7. LYN isoforms have similar localisation but different binding partners (related to Figure 6). **(A)** Details of LYN isoforms and exon structure. **(B)** Localisation of LYNA-GFP and LYNB-GFP fusion proteins in transiently transfected MDA-MB-231 cells. Scale bar, 20 μm. **(C)** Representative western blot showing total LYN knock-down (shLyn) and LYN-A*, LYN-B* or LYN-A*^{Y32F} mutant reconstitution using shLyn-resistant forms (LYN-A*, B* or A*^{YF32}) in MDA-MB-231 cells. **(D)** Heatmap visualization of relative abundance levels of LYN co-immunoprecipitated proteins from shLyn + LYN-A*, LYN-B* or LYN-A*^{YF32} MDA-MB-231 cells. Each column represents a protein and the colour in each slot indicates its corresponding abundance. Note that the LYN-A*^{YF32} sample is more similar to LYN-A* than LYN-B*.

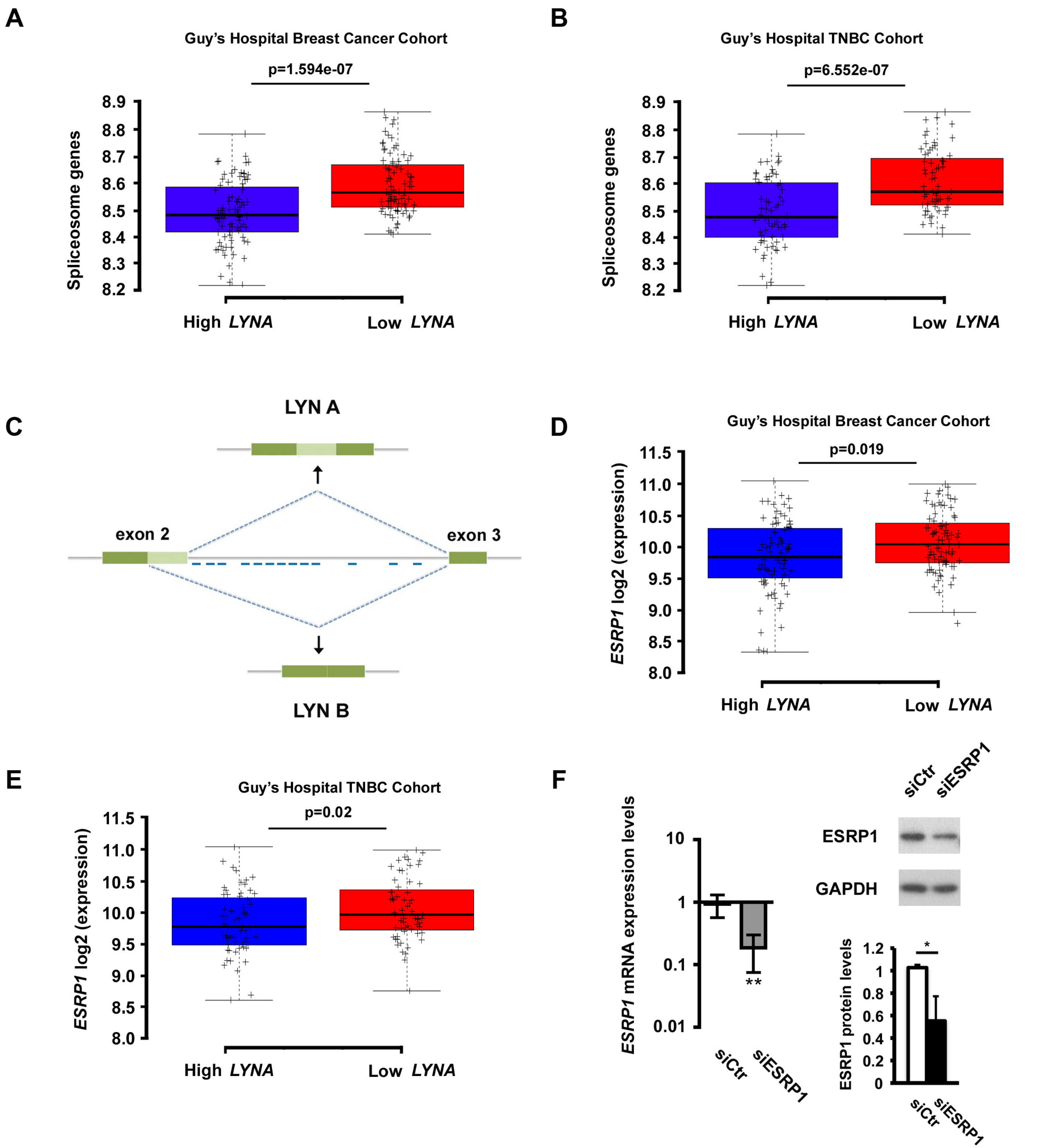
A
Guy's Hospital Breast Cancer Cohort
p=1.594e-07
Spliceosome genes
High LYNA
Low LYNA
B
Guy's Hospital TNBC Cohort
p=6.552e-07
Spliceosome genes
High LYNA
Low LYNA
C
LYN A
exon 2
exon 3
LYN B
D
Guy's Hospital Breast Cancer Cohort
p=0.019
ESRP1 log2 (expression)
High LYNA
Low LYNA
E
Guy's Hospital TNBC Cohort
p=0.02
ESRP1 log2 (expression)
High LYNA
Low LYNA
F
ESRP1 mRNA expression levels
siCtr
siESRP1
**
ESRP1
GAPDH
ESRP1 protein levels
*

Figure S8. LYN splicing is regulated by ESRP1 (related to Figure 6). (A, B) Boxplots showing the expression of a set of genes involved in splicing regulation (spliceosome) across 176 breast cancers **(A)** or 126 TNBCs divided based on *LYN A* transcript expression **(B)**. The significance of expression level differences between the two subgroups were evaluated with two-sided Student's t-tests. **(C)** Schematic of *LYN* transcript alternative splicing. Use of an alternative 5' (donor) splicing site within exon 2 results in the generation of LYN B transcript. UGG-rich motifs, putative binding elements for ESRP1 (Warzecha et al., 2010), are indicated by blue bars. **(D, E)** Boxplots showing *ESRP1* expression across 176 breast cancers **(D)** or 126 TNBC **(E)** divided based on *LYN A* transcript expression. The significant expression level differences between the two subgroups were evaluated with two-sided Student's t-tests. **(F)** MCF7 cells were transfected with control (siCtr) or *ESRP1* (siESRP1) siRNA. 72 hrs after transfection, *ESRP1* knock-down was assessed by qrtPCR (left; fold expression over comparator siCtr cells; mean±95% confidence intervals; n=3 independent experiments each of 3 technical replicates per sample) and western blot (right; mean+SD; n=3 independent experiments; two-tailed unpaired t test; $^{**}P<0.01$).

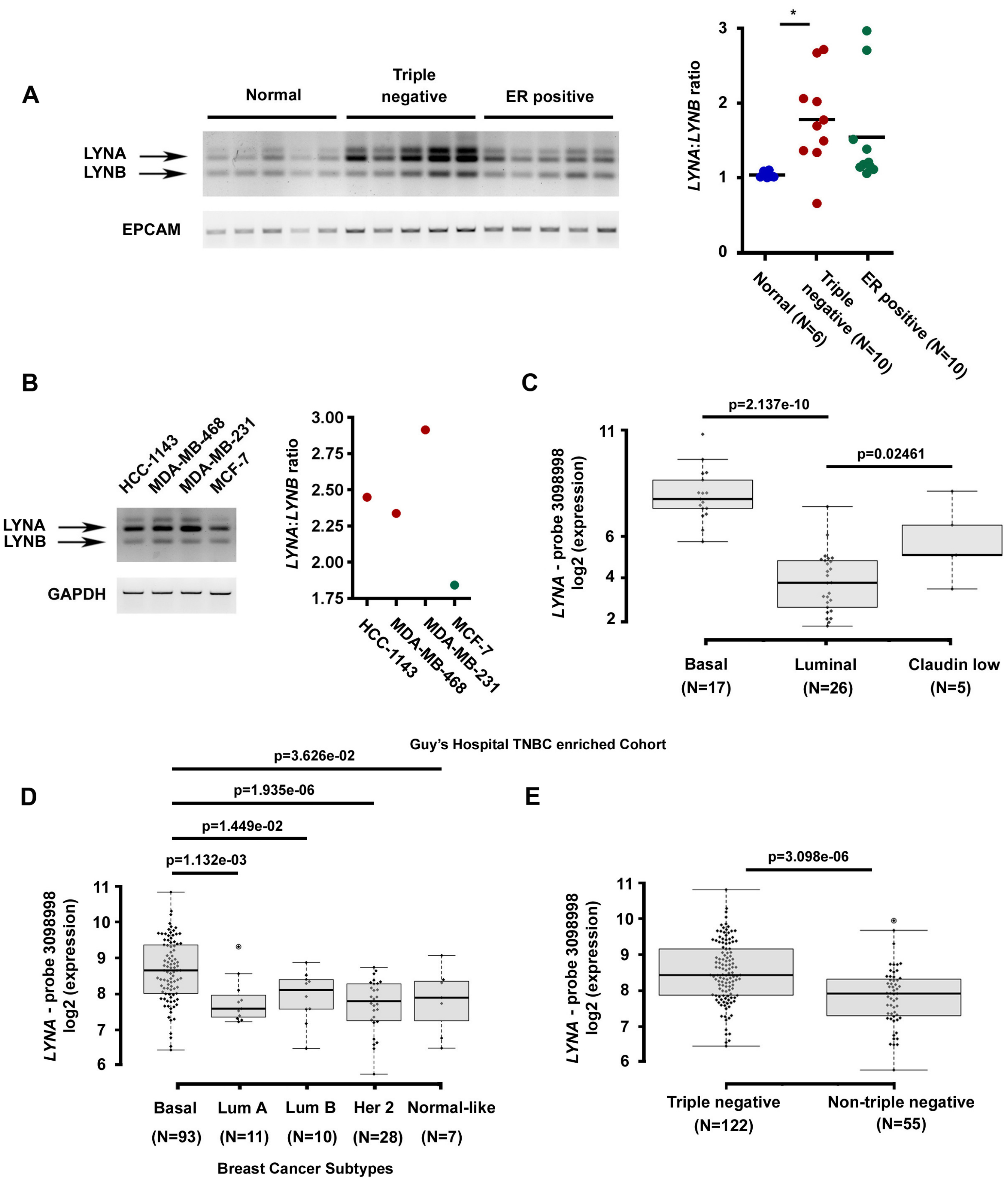

A
Normal
Triple negative
ER positive
LYNA
LYNB
EPCAM
*
LYNA:LYNB ratio
Normal (N=6)
Triple negative (N=10)
ER positive (N=10)
B
HCC-1143
MDA-MB-468
MDA-MB-231
MCF-7
GAPDH
C
p=2.137e-10
p=0.02461
LYNA - probe 3098998 log2 (expression)
Basal (N=17)
Luminal (N=26)
Claudin low (N=5)
Guy's Hospital TNBC enriched Cohort
D
p=3.626e-02
p=1.935e-06
p=1.449e-02
p=1.132e-03
Basal (N=93)
Lum A (N=11)
Lum B (N=10)
Her 2 (N=28)
Normal-like (N=7)
Breast Cancer Subtypes
E
p=3.098e-06
Triple negative (N=122)
Non-triple negative (N=55)

Figure S9. LYNA expression in TN/BLBC is significantly higher than in other breast cancer subtypes (related to Figure 7). (A) Analysis of *LYNA* and *LYNB* expression in human breast tissue by RT-PCR. Arrows indicate PCR products of the expected size using primers spanning the *LYN* splicing insert. *EPCAM*, an epithelial marker, was used for normalizing to control for possible differences in amount of epithelium in tissue samples. Left panel, gels showing representative PCR results. Note the band above the *LYNA* band results from hetero-hybridization of *LYNA* and *LYNB* PCR products. Right panel, quantitation of *LYNA*:*LYNB* ratio (normal tissue from reduction mammoplasty, blue; TNBC, red; ER-positive breast cancer, green)**.** Bars indicate mean values. *$P<0.05$, unpaired two-tailed t-test. **(B)** RT-PCR analysis and quantitation of *LYNA*, *LYNB* and *GAPDH* expression in a panel of human breast cancer cell lines. MCF-7, ER-positive breast cancer cell line; MDA-MB-231, MDA-MB-468, HCC1143, TN breast cancer cell line. **(C, D, E)** Boxplots showing the *LYN A* isoform expression across breast cancer cell lines divided based on their subtypes **(C)**, across 177 breast cancers classified based on their PAM50 breast cancer subtypes **(D)** or their immune-histochemical defined subtypes **(E)**. The significant expression level differences between the different subgroups were evaluated with two-tailed t-tests.

P4 = Luminal progenitors; P5 = Luminal differentiated; P6 = stroma; P7 = basal/stem

BP213

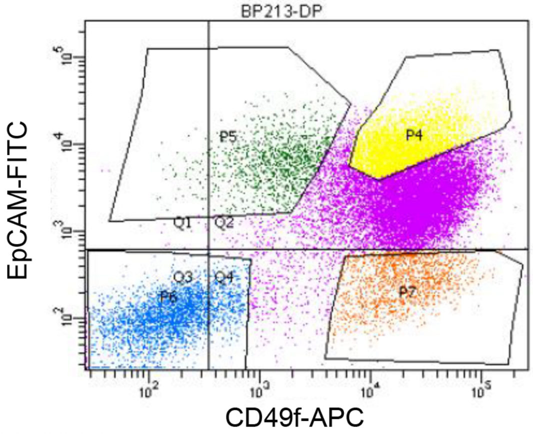

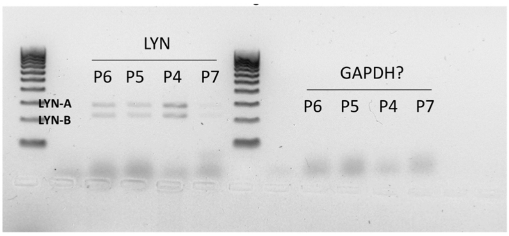

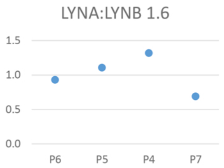

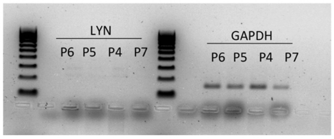

Comment: GAPDH failed in first PCR analysis; LYN very weak in second analysis

BP228

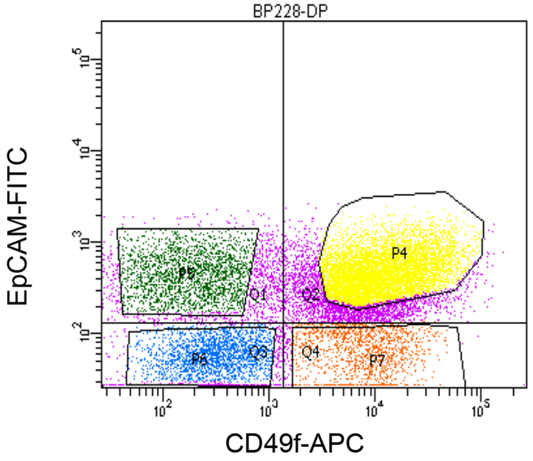

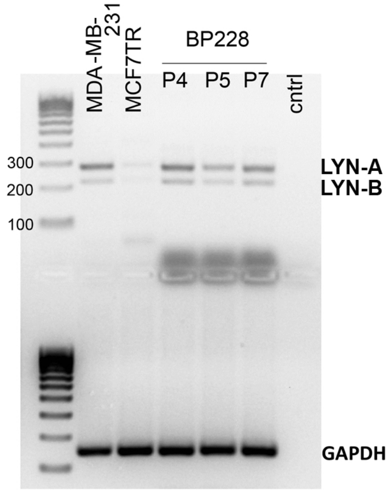

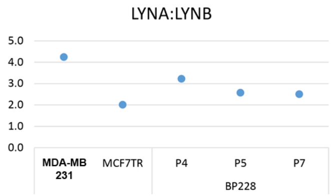

Comment: P6 stroma material insufficient for analysis

BP236

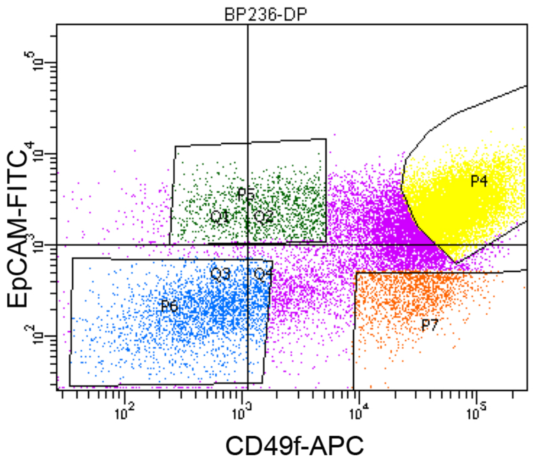

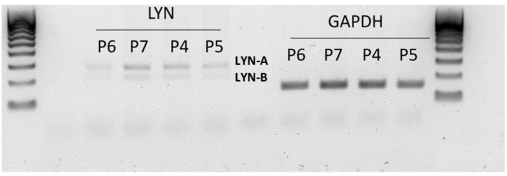

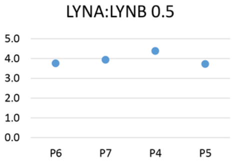

BP257

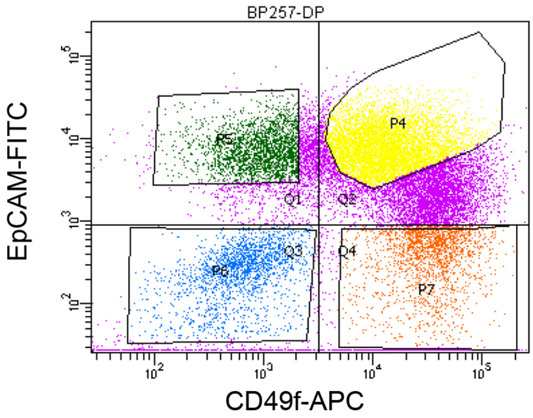

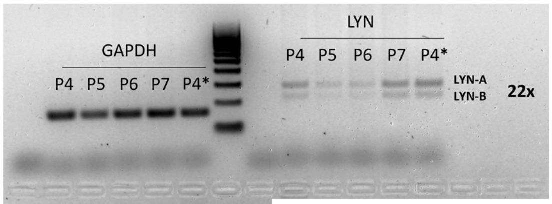

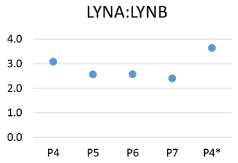

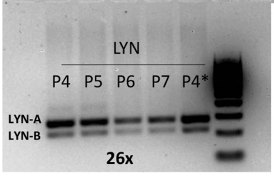

Comment: Analysis done with both 22 and 26 cycles; P4 populations collected twice with two different gates drawn, P4 and P4*. Plot shows P4*.

Figure S10. Isolation of primary normal human breast epithelial cells from reduction mammoplasty tissue (related to Figure 7). EpCAM-FITC and CD49f-APC dot plots and semi-quantitative rtPCR analysis results of *LYN* isoform expression from four independent breast preparations. Identity of the gated populations is indicated and based upon previous studies.
