# Supplementary material for: Dual Mechanisms of LYN Kinase Dysregulation Drive Aggressive Behavior in Breast Cancer Cells
Source: Cell Rep. 2018 Dec 26;25(13):3674–3692.e10. doi: 10.1016/j.celrep.2018.11.103 (PMC6315108; doi:10.1016/j.celrep.2018.11.103)
Supplement: Document S2. Article plus Supplemental Information [file mmc5.pdf]

Cell Reports

Article

Dual Mechanisms of LYN Kinase Dysregulation Drive Aggressive Behavior in Breast Cancer Cells

Graphical Abstract

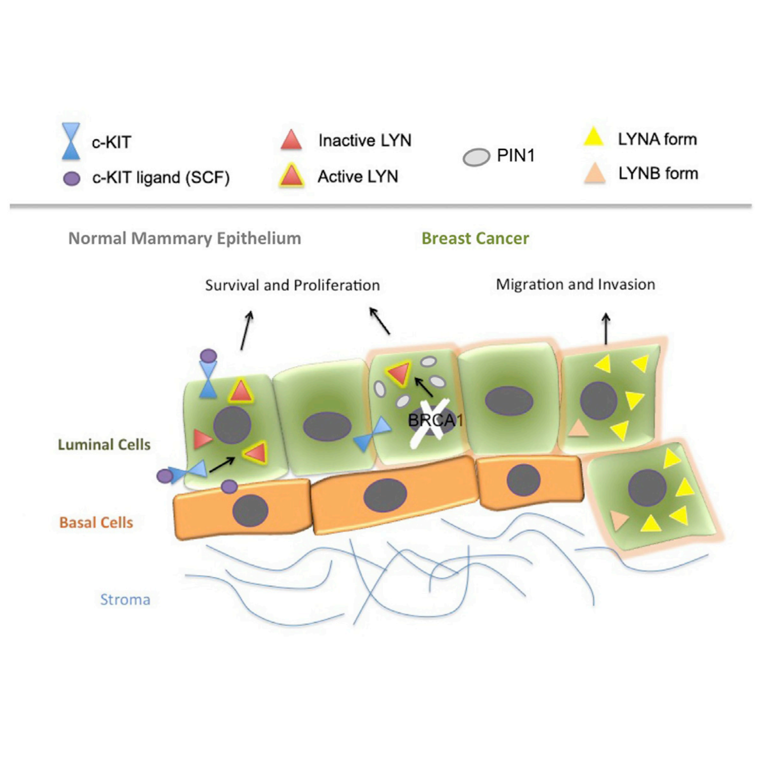

Highlights

- LYN kinase is a downstream effector of the c-KIT receptor in normal breast cells
- Loss of BRCA1 function hyperactivates LYN via prolyl isomerase 1 upregulation
- The full-length LYN isoform promotes tumor cell invasion
- Time to breast cancer death is shorter in tumors with a high LYNA::B isoform ratio

Authors

Giusy Tornillo, Catherine Knowlson, Howard Kendrick, ..., Niamh E. Buckley, Anita Grigoriadis, Matthew J. Smalley

Correspondence

smalleymj@cardiff.ac.uk

In Brief

Tornillo et al. show that in aggressive breast cancers, LYN activity is deregulated by a change in patterns of splice isoform expression. In *BRCA1*-dysfunctional breast cancers, LYN activity is upregulated by a prolyl isomerase (PIN1) that is normally repressed by BRCA1.

Tornillo et al., 2018, Cell Reports *25*, 3674–3692
December 26, 2018 © 2018 The Author(s).
https://doi.org/10.1016/j.celrep.2018.11.103

CellPress

OPEN ACCESS
CellPress

Cell Reports
Article

Dual Mechanisms of LYN Kinase Dysregulation Drive Aggressive Behavior in Breast Cancer Cells

Giusy Tornillo,[1] Catherine Knowlson,[2] Howard Kendrick,[1] Joe Cooke,[1] Hasan Mirza,[3] Iskander Aurrekoetxea-Rodríguez,[4] Maria d.M. Vivanco,[4] Niamh E. Buckley,[5,6] Anita Grigoriadis,[3,6] and Matthew J. Smalley[1,7,*]
[1]European Cancer Stem Cell Research Institute, School of Biosciences, Hadyn Ellis Building, Cardiff University, Cardiff CF24 4HQ, UK
[2]Centre for Cancer Research and Cell Biology, Queens University Belfast, 97 Lisburn Rd, Belfast BT9 7AE, UK
[3]School of Cancer & Pharmaceutical Sciences, CRUK King's Health Partners Centre, King's College London, Innovation Hub, Comprehensive Cancer Centre at Guy's Hospital, Great Maze Pond, London SE1 9RT, UK
[4]Center for Cooperative Research in Biosciences, CIC bioGUNE, 48160 Derio, Spain
[5]School of Pharmacy and Centre for Cancer Research and Cell Biology, Queens University Belfast, 97 Lisburn Rd, Belfast BT9 7AE, UK
[6]These authors contributed equally
[7]Lead Contact
*Correspondence: smalleymj@cardiff.ac.uk
https://doi.org/10.1016/j.celrep.2018.11.103

SUMMARY

The SRC-family kinase LYN is highly expressed in triple-negative/basal-like breast cancer (TNBC) and in the cell of origin of these tumors, c-KIT-positive luminal progenitors. Here, we demonstrate LYN is a downstream effector of c-KIT in normal mammary cells and protective of apoptosis upon genotoxic stress. LYN activity is modulated by PIN1, a prolyl isomerase, and in *BRCA1* mutant TNBC PIN1 upregulation activates LYN independently of c-KIT. Furthermore, the full-length LYN splice isoform (as opposed to the Δaa25–45 variant) drives migration and invasion of aggressive TNBC cells, while the ratio of splice variants is informative for breast cancer-specific survival across all breast cancers. Thus, dual mechanisms—uncoupling from upstream signals and splice isoform ratios—drive the activity of LYN in aggressive breast cancers.

INTRODUCTION

Breast cancers molecularly classified as basal-like breast cancer typically display the triple (ER/PR/HER2)-negative (TNBC) phenotype (Badve et al., 2011). The molecular etiology of sporadic TNBC is still poorly understood, although germline *BRCA1* mutations predispose to TNBC, and *BRCA1* silencing or dysfunction in the BRCA1 pathway can be found in sporadic TNBC (Badve et al., 2011). Limited therapeutic options are available for TNBC; chemotherapy is often initially beneficial, but TNBC has a high risk of relapse (Liedtke et al., 2008), emphasizing the need to elucidate its biology and identify targets for novel treatment options.

The mammary epithelium consists of luminal cells, including ER-negative (ER−) progenitor-like and ER-positive (ER+) differentiated cells, and basal cells. TNBC likely originates from luminal ER− progenitors, and the gene expression profile of both *BRCA1* mutation-associated and sporadic TNBC reflects a luminal progenitor-like profile (Lim et al., 2009; Molyneux et al., 2010). Elucidating the molecular regulation of this cell subset is important to understand not only the normal mammary cell homeostasis but also the origins of TNBC.

Mammary ER− luminal progenitors are characterized by expression of the membrane tyrosine kinase receptor c-KIT (Regan et al., 2012; Smart et al., 2011), which is required for growth and survival of these cells (Regan et al., 2012; Tornillo et al., 2013) as well as the SRC family tyrosine kinase (SFK) LYN (Bach et al., 2017; Regan et al., 2012; Smart et al., 2011), a known effector of c-KIT signaling in hematopoietic cells (Shivakrupa and Linnekin, 2005). Other SFKs are expressed in the mammary epithelium, but other than LYN, only FYN has an expression pattern restricted to a specific population (basal epithelial cells) (Bach et al., 2017; Kendrick et al., 2008). Based on this co-expression, a c-KIT-LYN signaling axis in mammary epithelial progenitors is proposed.

Previous studies have largely focused on LYN function in hematopoietic cells and leukemia, and persistent activation and/or deregulation of LYN has been associated with imatinib resistance in BCR-ABL+ leukemia (Wu et al., 2008). In breast cancer, LYN has been reported as overexpressed and a potential drug target in TNBC by several studies (Choi et al., 2010; Hochgräfe et al., 2010; Molyneux et al., 2010; Regan et al., 2012; Smart et al., 2011). LYN point mutations in breast cancer are rare (0.6%) (https://cancer.sanger.ac.uk/cosmic), but have been associated with anti-estrogen resistance in a subset of ER+ tumors (Schwarz et al., 2014); only 6%–10% of breast cancers show *LYN* amplification (http://www.cbioportal.org/index.do; https://cancer.sanger.ac.uk/cosmic). Other mechanisms contributing to the underlying LYN dysregulation in TNBC remain to be defined, as does the potential wider role of LYN in breast cancer.

Here we demonstrate that LYN kinase is a transducer of c-KIT growth signals in the normal mammary epithelium. We show that LYN can also be activated by prolyl isomerase 1 (PIN1), normally transcriptionally repressed by BRCA1. In *BRCA1*-deficient TNBC, loss of this transcriptional repression results in increased PIN1 levels and thus in LYN activation independently of c-KIT. Furthermore, we address the role of the two LYN splice isoforms

 © 2018 The Author(s).
This is an open access article under the CC BY license (http://creativecommons.org/licenses/by/4.0/).

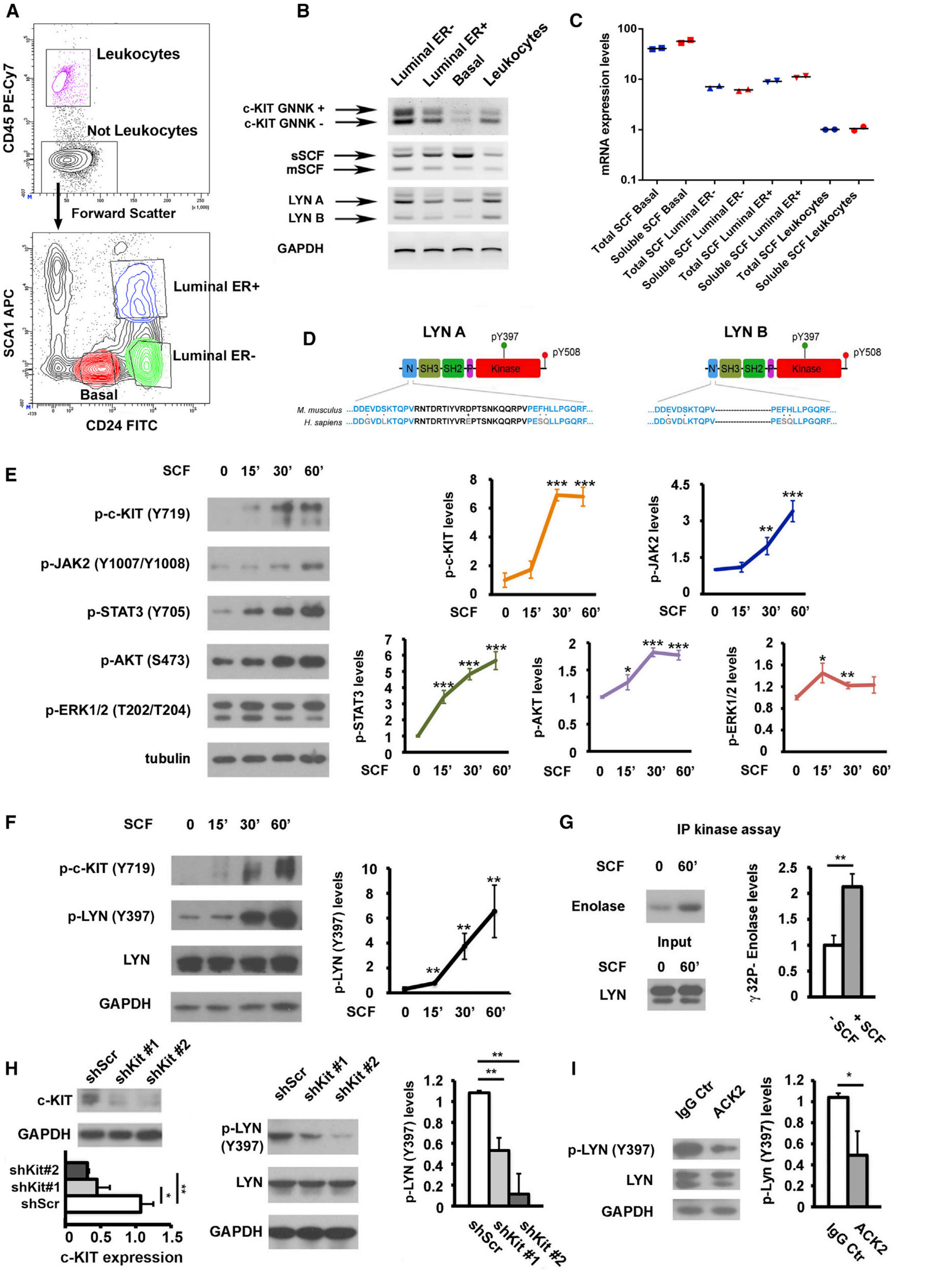

(legend on next page)

in breast cancer and find that only full-length LYN (LYNA), as opposed to LYN$^{\Delta 25-45}$ (LYNB), promotes cell migration and invasion. *LYNA* is expressed more highly in TNBC than other breast cancer types; however, we find that a higher ratio of *LYNA* over *LYNB* is present in breast cancers of patients with shorter survival times, irrespective of tumor subtype. Therefore, our findings demonstrate dual mechanisms, uncoupling from upstream signals and changing splice isoform ratios, driving the activity of LYN in aggressive breast cancers. These mechanisms have the potential to be targeted therapeutically, and the *LYNA::B* ratio is a biomarker that could identify patients who would benefit from such interventions.

RESULTS

LYN Kinase Is Regulated by c-KIT and Promotes Growth of Normal Mammary Epithelial Cells

To define the major components of the c-KIT signaling network in the mammary epithelium, we examined expression of c-KIT and its ligand stem cell factor (SCF) in normal mouse mammary cell populations (Figure 1A). The two splice variants of c-KIT, GNNK+,and GNNK−, were expressed primarily in luminal cells (particularly in the ER− luminal subpopulation) (Figure 1B). The two SCF isoforms, soluble SCF (sSCF) and membrane-bound SCF (mSCF), were present at low levels in luminal cells, whereas basal cells showed the highest levels of total SCF, with almost exclusive expression of the sSCF form (Figures 1B and 1C).

LYN is a key effector of c-KIT signaling in hematopoietic cells, and LYN expression and c-KIT expression in the mammary gland are correlated (Regan et al., 2012; Roskoski, 2005). LYN exists in two isoforms, LYNA (full-length LYN) and LYNB (LYN$^{\Delta 25-45}$) (Figure 1D). When expression of these isoforms was analyzed by semiquantitative RT-PCR, both *LynA* and *LynB* were found in all mammary epithelial populations; however, there was an association between higher *LynA* and *c-Kit* expression in the luminal ER− compartment (Figure 1B). Therefore, the expression pattern of c-KIT, SCF, and full-length LYN in the mammary epithelium indicated the existence of a basal-to-luminal paracrine c-KIT signaling network, mediated by the soluble form of SCF (sSCF), along with an enrichment of a potential c-KIT effector, LYN, in the SCF-responsive luminal cells.

To determine the signaling cascade activated by c-KIT, we treated primary mouse mammary epithelial cells with SCF and assessed the phosphorylation status of a series of previously described c-KIT effectors (Roskoski, 2005). Addition of SCF caused a marked increase in c-KIT phosphorylation, as well as upregulation of phosphorylation levels of JAK2, STAT3, AKT, and ERK1/2 with distinctive kinetics (Figure 1E). Phosphorylation levels of LYN at its positive regulatory site Y397 were elevated approximately 6-fold within 60 min of stimulation with SCF (Figure 1F), and SCF treatment induced an increase in LYN kinase activity as measured by an immunoprecipitation (IP) kinase assay (Figure 1G). Conversely, c-KIT inhibition, by using short hairpin RNA (shRNA) against c-KIT or a specific anti-c-KIT blocking antibody (ACK2), led to a significant decrease in LYN phosphorylation (Figures 1H and 1I; Figure S1A).

Because c-KIT is required for growth of normal mammary cells *in vitro* (Regan et al., 2012) and positively regulated LYN activity, we tested whether LYN depletion also affected mammary cell growth. Following *Lyn* knockdown with two distinct shRNAs (shLyn1 or shLyn2) (Figure 2A), primary mouse mammary epithelial cells exhibited defective growth (Figure 2B) and a significant reduction in the expression of the proliferation marker Ki67 (Figure 2C). This effect was observed both in unsorted primary mammary epithelial cells and in the purified luminal ER− progenitor population (Figures 2D and 2E; Figure S1B). Furthermore, knockdown of *LYN* in the human normal mammary epithelial cell line, MCF10A, with two distinct shRNAs caused a significant reduction in relative cell growth and in Ki67 expression compared to shScrambled (shScr) controls (Figures 2F and 2G), without obviously affecting acinar architecture (Figure S1C).

We next tested the ability of a constitutively active LYN (Y508F) mutant to rescue c-KIT knockdown. Whereas overexpression of wild-type LYNA (LYNA WT) had no effect on the viability of c-KIT knockdown cells, constitutively active LYN (LYNA CA) rescued the growth defect (Figure S2A). In addition, when we examined the ability of LYN-depleted cells to activate c-KIT downstream effectors in response to SCF, we found that LYN knockdown specifically interfered with AKT phosphorylation upon c-KIT stimulation (Figure S2B). Overall, these findings support the model that c-KIT activates LYN kinase to transduce

Figure 1. LYN Is Positively Regulated by c-KIT in Normal Mammary Cells

(A) Flow cytometry of primary mammary cells stained with CD45, CD24, and Sca-1 antibodies. CD45^{+} leukocytes (purple) were gated out (top plot), and CD45^{-} cells (bottom plot) were gated to define basal (CD24$^{+/low}$ Sca-1^{-}, red), luminal ER− (CD24$^{+/high}$ Sca-1^{-}, green), and luminal ER+ (CD24$^{+/high}$ Sca-1^{+}, blue) epithelial cell populations.

(B) Expression pattern of *c-Kit*, *Scf*, and *Lyn* splicing transcripts in mouse mammary cell populations. Semiquantitative RT-PCR data are representative of two independent isolates (four mice for each). Amplicons of the expected size using primers spanning the alternative exon for each gene are indicated. *Gapdh* was used as a control.

(C) qRT-PCR gene expression analysis of *Scf* in mouse mammary cell populations using probes for both total *Scf* (membrane bound and soluble) or soluble *Scf* (*sSCF*) only. Data are from two independent isolates (four mice for each), presented as relative expression levels with leukocytes as the comparators.

(D) Schematic of LYN isoforms showing the 21-amino acid insertion (black residues) in the N-terminal domain of LYNA.

(E) Representative western blot analysis and quantitation of c-KIT, JAK2, STAT3, AKT, and ERK1/2 phosphorylation levels in protein extracts from primary mouse mammary organoids cultured on Matrigel and stimulated with SCF for the indicated times. Tubulin was used as loading control.

(F and G) Representative western blot analysis and quantitation of LYN autophosphorylation (Y397) (F) and immunoprecipitation (IP) LYN kinase assay of protein extracts (G) from primary mouse mammary organoids cultured on Matrigel and stimulated with SCF for 0, 15, 30, and 60 min.

(H and I) Western blot of c-Kit expression and LYN autophosphorylation (Y397) in primary mouse mammary organoids after transduction with control (shScr) or c-Kit knockdown (shKit1 and shKit2) lentiviruses (H) or following treatment with c-KIT blocking (ACK2) or immunoglobulin G (IgG) isotype (IgG Ctr) antibodies (I). Unless otherwise stated, blots are representative of three independent experiments (mean and SD; two-tailed unpaired t tests) (in E and F, t tests are relative to time 0). *p < 0.05; **p < 0.01; ***p < 0.001. See also Figure S1.

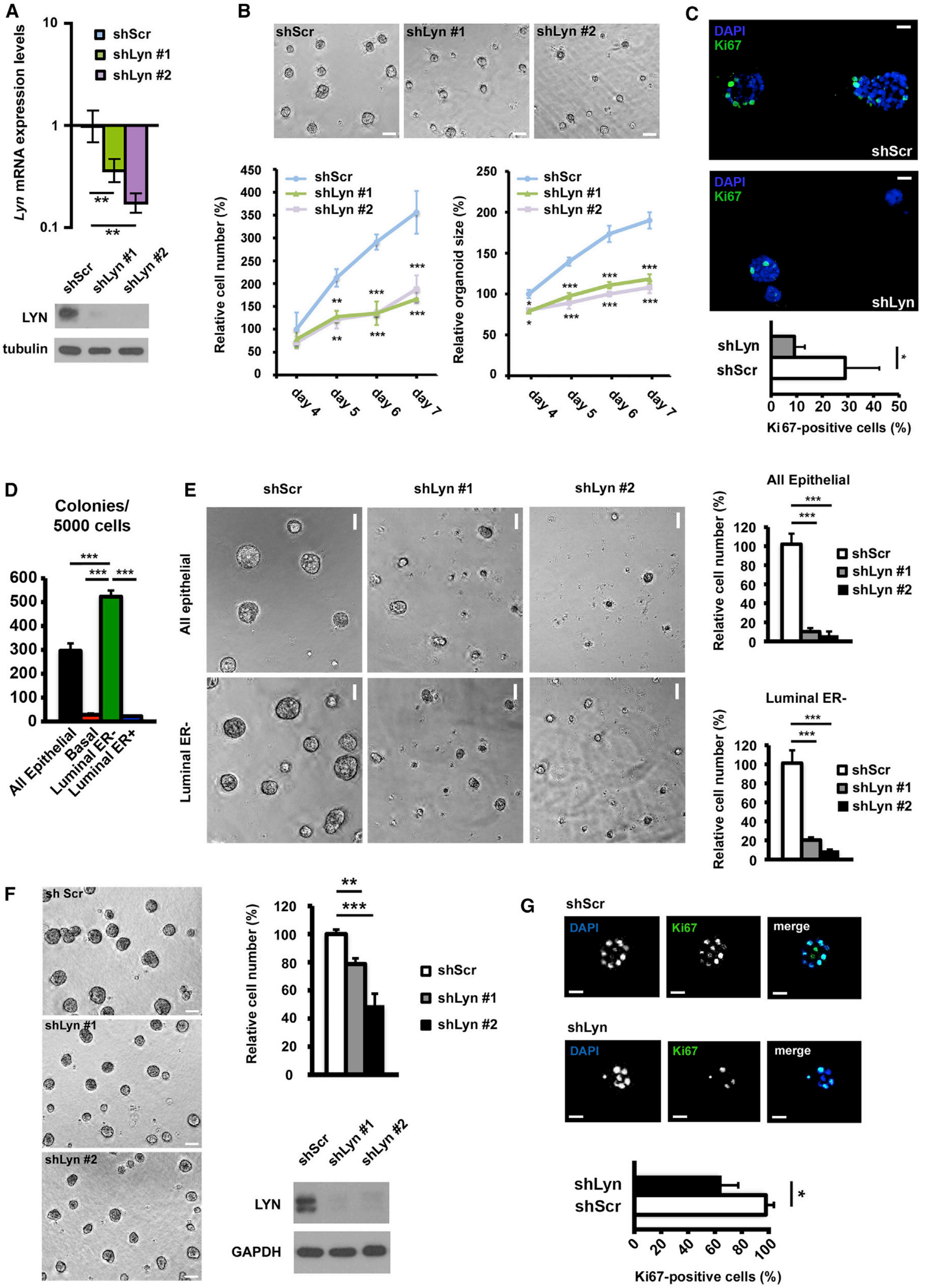

(legend on next page)

pro-growth and survival signals and activate the AKT pathway in mammary epithelial cells.

LYN Is Required for Growth of BRCA1-Deficient Mammary Tumor Cells

We have previously demonstrated that *Brca1* mutation-associated breast cancers originate from luminal ER− progenitors (Molyneux et al., 2010) and that c-KIT and LYN are expressed in mouse *Brca1* mammary tumors (Regan et al., 2012). To determine whether *Brca1* mutant cell growth depends on the activation of the c-KIT signaling pathway, primary mouse *Brca1* mutant mammary tumor cells transduced with lentiviruses expressing either one of two shRNAs against *c-Kit* (shKit1 and shKit2) or a control shRNA (shScr) were analyzed. Despite reduced *c-Kit* expression, no change in cell growth was observed in shKit cells compared to shScr cells (Figure S2C). Furthermore, unlike normal cells, *c-Kit*-depleted tumor cells had phospho-LYN levels similar to those of control cells (Figure S2D) and treatment of *Brca1* tumor cells with the ACK2 c-KIT blocking antibody did not alter LYN phosphorylation status (Figure S2E; contrast with Figure 1I). Likewise, c-KIT knockdown failed to affect phospho-LYN levels in three human c-KIT-positive breast cancer cell lines with low BRCA1 levels: HCC38 (*BRCA1* silenced by methylation), HCC1806, and MDA-MB-157 (BRCA1 low due to downregulation by microRNA [miRNA]) (Garcia et al., 2011; Li et al., 2013) (Figure S3A). However, c-KIT knockdown in a c-KIT-positive BRCA1-wild-type cell line, HCC1187, suppressed LYN phosphorylation (Figure S3A). These results indicate that in *Brca1*/*BRCA1* tumor cells, at least *in vitro*, c-KIT is dispensable for growth and does not regulate LYN activity.

Next, we evaluated the effects of LYN knockdown on *Brca1* tumor cell growth. LYN knockdown markedly impaired growth of mouse *Brca1* tumor-derived cells in monolayer culture (Figure 3A) and in three-dimensional (3D) culture conditions on Matrigel (Figure 3B). Staining of 3D-cultured tumor cells for the proliferation marker Ki67 showed that the number of proliferating cells was reduced by approximately 30% in shLyn-transduced cultures compared with control (Figure 3C). The kinase activity of LYN was required for its pro-survival functions, because expression of shRNA-resistant wild-type LYNA (LYNA*WT) was able to rescue the effect of shLyn transduction, but expression of a kinase-dead LYNA (T410K) mutant (LYNA*KD) was unable to do so (Figure 3D). The broad spectrum kinase inhibitor Dasatinib, which was able to block LYN Y397 phosphorylation in mammary epithelial cells in a dose-dependent manner (Figure S3B), inhibited growth of three mouse BRCA1 tumor-derived cell lines (half-maximal inhibitory concencetration [IC_{50}] 0.1–1 μM) and the human BRCA1 mutant HCC1937 line (IC_{50} 0.1 μM) (Figures S3C and S3D).

Use of two short hairpins targeting human *LYN* (Figure 3E) demonstrated that *LYN* knockdown in human breast cancer cells also significantly impaired cell growth in the *BRCA1*-mutated HCC1937 human breast cancer cell line (Figure 3F) and in cells from a *BRCA1* mutant breast cancer patient-derived xenograft (PDX) (Figure 3G). These effects, therefore, were consistent in both mouse and human cells.

Because LYN blockade effectively suppressed tumor cell growth *in vitro*, we next evaluated the effects of blocking LYN activity *in vivo*. Intraperitoneal (i.p.) injection of Dasatinib strongly reduced LYN phosphorylation in the normal mammary epithelium of wild-type mice (Figure S3E), and daily treatment with Dasatinib significantly inhibited growth of *BlgCre Brca1*$^{fl/fl}$ *p53*$^{+/-}$ tumors (Figures S3F and S3G). Immunohistochemical staining for phospho-histone H3 (phospho-H3) showed a lower number of mitotic cells in Dasatinib-treated compared to vehicle-treated tumors (Figure S3H).

Reduction of cell numbers following constitutive *Lyn* knockdown made testing the effects of specific *Lyn* depletion by shRNA on tumor cell growth *in vivo* difficult. Therefore, a conditional *Lyn* knockdown system in which mouse *Brca1* tumor cells expressed shRNA against *Lyn* under the control of doxycycline was established. Analysis of *Lyn* transcript levels after exposure to doxycycline confirmed that *Lyn* expression was reduced in inducible shLyn-carrying cells in the presence of doxycycline

Figure 2. LYN Promotes Normal Mammary Cell Growth

(A) Analysis of *Lyn* expression levels by qRT-PCR relative to shScr cells (top) and western blot (bottom) in primary mouse mammary organoids 4 days after transduction with control (shScr) or *Lyn* knockdown (shLyn1 and shLyn2) lentiviruses.

(B) Growth of mammary organoids after transduction with control (shScr) or *Lyn* knockdown (shLyn1 and shLyn2) lentiviruses, assessed by cell number change (left bottom panel) or organoid size (right bottom panel) relative to shScr cells, day 4 after plating. Top panels: representative images show organoids at day 6 of culture. Scale bar, 75 μm.

(C) Ki67 immunofluorescence staining (green) of control (shScr)- and shLyn-carrying mammary organoids 6 days after lentiviral transduction (DAPI nuclear counterstaining). Representative images and quantification of the percentage of Ki67-positive cells. Scale bar, 20 μm.

(D) Colony-forming potential of unfractionated primary mammary epithelial cells (all epithelial) or basal, luminal ER−, and luminal ER+ subpopulations. 5,000 cells from each fraction were plated on Matrigel, and colony numbers were determined after 12–14 days.

(E) Growth inhibition of unfractionated primary mammary epithelial cells (all epithelial) or the luminal ER− fraction transduced with control (shScr) or Lyn knockdown (shLyn1 and shLyn2) lentiviruses and seeded onto Matrigel. Cell growth was assessed after 12–14 days. Representative images, left (scale bar, 100 μm); quantitation, right.

(F) Growth inhibition of MCF10A cells transduced with lentiviral vectors carrying control shRNA (shScr) or shRNA against LYN (shLyn1 and shLyn2). Transduced cells were grown in 3D on Matrigel, and relative cell numbers were assessed after 12 days of culture. Representative images of acinar structures (day 12) derived from shScr, shLyn1, or shLyn2 cells are shown (scale bar, 100 μm), together with CellTiterGlo quantitation and assessment of LYN knockdown by western blot (GAPDH loading control).

(G) Confocal microscope analysis of Ki67 immunofluorescence-stained shScr, shLyn1, or shLyn2 knockdown MCF10A cells at day 4 of culture in 3D with quantitation. DAPI was used for counterstaining. Scale bar, 20 μm.

Blots are representative of three independent experiments. Quantitation, mean and SD (n = 3; two-tailed unpaired t tests) except for gene expression analysis by quantitative real-time RT-PCR (mean ± 95% confidence intervals; significance of real-time RT-PCR data was determined from confidence intervals; n = 3 independent experiments for each of 3 technical replicates per sample) (Cumming et al., 2007). *p < 0.05; **p < 0.01; ***p < 0.001. See also Figures S1–S3.

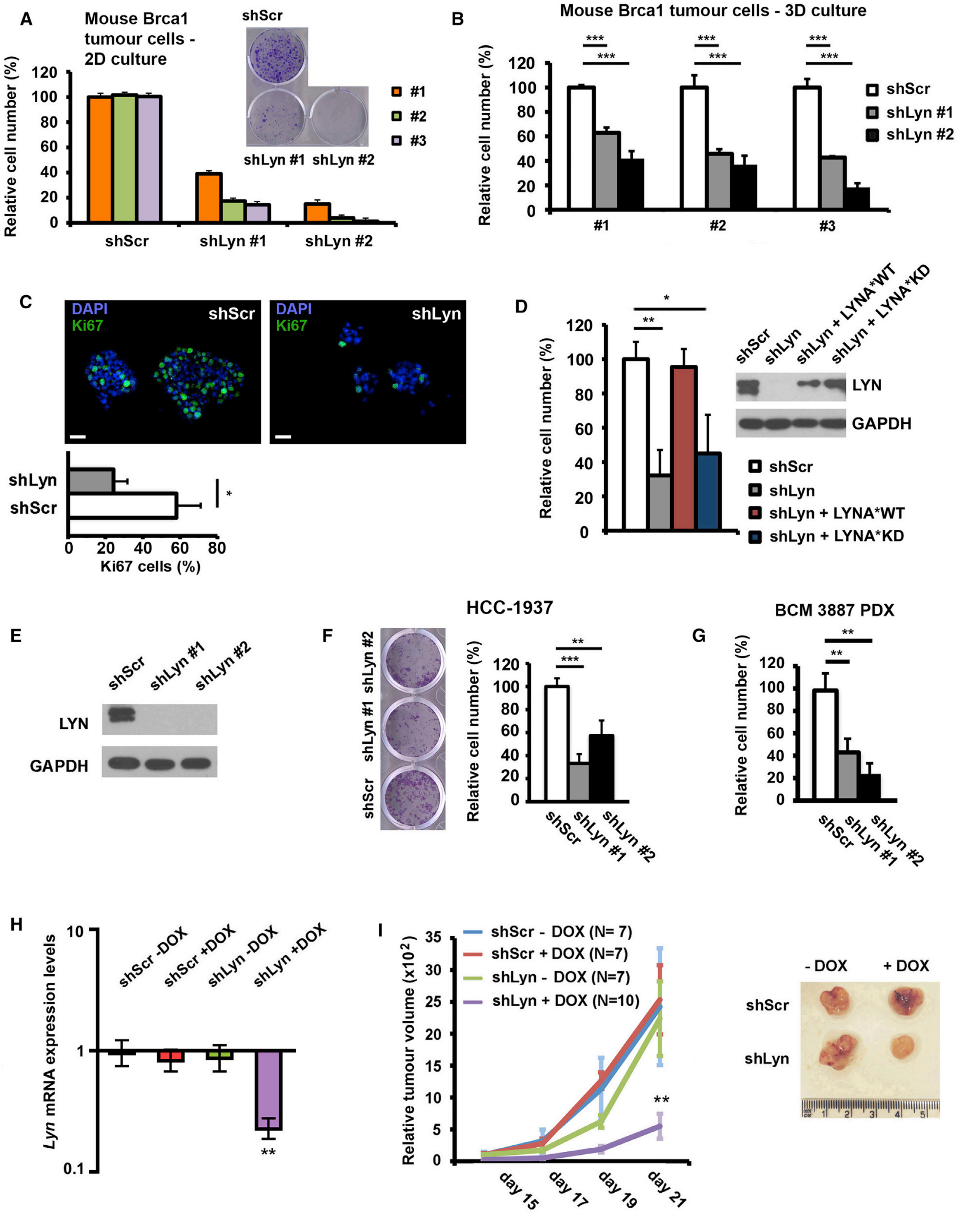

(legend on next page)

(Figure 3H). Upon orthotopic cell injection in immunodeficient mice, administration of doxycycline resulted in a significant decrease in the growth of tumors derived from cells carrying inducible anti-*Lyn* shRNA (Figure 3I). Staining of tumor sections for the mitotic cell marker phospho-H3 revealed a reduction in the number of mitotic cells in samples from doxycycline-treated shLyn tumors compared with controls (Figure S3I). Therefore, LYN kinase depletion suppresses *Brca1* mammary tumor cell growth both *in vitro* and *in vivo*.

Brca1 Depletion Leads to Upregulation of LYN Kinase Activity in a PIN1-Dependent Manner

Our data show that in normal mammary epithelial cells, LYN kinase activity is under the strict control of the c-KIT receptor, whereas in *Brca1* mutant tumor cells, LYN functions independently of c-KIT. We hypothesized that inactivation of *Brca1* might contribute to dysregulation of LYN kinase activity. First, we analyzed a panel of TNBC cell lines for LYN and phospho-LYN (Y397) levels. Three of the lines (MDA-MB-436, SUM-149, and HCC1937) carry inactivating *BRCA1* mutations, one (HCC38) has *BRCA1* promoter methylation, four (MDA-MB-157, HCC1806, MDA-MB-468, and HCC70) have been reported as having low *BRCA1* expression (Buckley et al., 2016; Garcia et al., 2011; Gong et al., 2015; Li et al., 2013), and six (MDA-MB-231, MDA-MB-453, BT-20, BT-549, HCC1143, and HCC1187) are *BRCA1* wild-type. Total LYN levels were variable across the lines; however, when phospho-LYN levels were normalized to total LYN, TNBC cells with defective *BRCA1* had significantly higher levels of phospho-LYN than those of wild-type cells (Figure 4A). Furthermore, *Brca1* knockdown in primary (normal) mouse mammary epithelial cells resulted in increased LYN phosphorylation but unchanged c-KIT phosphorylation (Figure 4B). Conversely, forced overexpression of hemagglutinin-tagged BRCA1 (HA-BRCA1) in primary mammary epithelial cells suppressed LYN phosphorylation (Figure 4C).

The prolyl isomerase PIN1 recognizes specific serine-proline or threonine-proline sequences in proteins, changing the conformation of the prolines within these sequences and resulting in altered activity of the target protein (Zhou and Lu, 2016). LYN contains potential PIN1 consensus target sequences (Pro197 and Pro229), and PIN1 is transcriptionally repressed by BRCA1 (MacLachlan et al., 2000). Therefore, we hypothesized that increased LYN activity following BRCA1 inactivation or depletion results from increased PIN1 levels and that PIN1 was activating LYN. To test this, we first used phospho-protein arrays to demonstrate that both BRCA1 overexpression and *PIN1* knockdown in MDA-MB-468 cells resulted in a significant reduction in phosphorylation of LYN, but not its close family member SRC (Figures S4A and S4B). Moreover, we confirmed that BRCA1 suppresses PIN1 expression by overexpressing *BRCA1* in MDA-MB-468 cells and showing that *PIN1* mRNA levels were reduced by approximately 50% (Figure S4C). We also compared PIN1 levels in mouse *Brca1* tumor cells and normal mouse mammary epithelium and confirmed that PIN1 levels were significantly higher in the tumor cells (Figure S4D).

Next, we stained a tissue microarray consisting of 15 germline *BRCA1* mutant and 15 sporadic TNBC cases. Cases from *BRCA1* patients showed, overall, significantly more intense PIN1 staining than did sporadic tumors (Figures 4D and 4E). Given our findings that *BRCA1* loss results in PIN1 upregulation, we hypothesized that even in sporadic breast cancers not linked to germline *BRCA1* mutation but that have low *BRCA1* levels through other mechanisms, levels of *BRCA1* and *PIN1* expression would be inversely correlated. We therefore

Figure 3. LYN Activity Is Required for Growth of *Brca1* Tumor Cells

(A) Primary cells isolated from three distinct *BlgCre Brca1*$^{fl/fl}$ *p53*$^{+/-}$ mouse mammary tumors (1–3) were transduced with control (shScr) or *Lyn* knockdown (shLyn1 and shLyn2) lentiviruses, seeded at low density in adherent conditions (2D), and stained with crystal violet after 6 days. Viable cell density was determined by absorbance measurement following solubilization of the dye. Representative images of tumor cell colonies at day 6 of culture are shown.

(B) shScr-, shLyn1-, or shLyn2-transduced *BlgCre Brca1*$^{fl/fl}$ *p53*$^{+/-}$ tumor cells (1–3) seeded in Matrigel (3D) were assessed for growth after 6 days. Graphs show cell number assessed at day 5 of culture relative to shScr cells.

(C) Ki67 immunofluorescence staining (green) of control (shScr)- and shLyn-transduced *BlgCre Brca1*$^{fl/fl}$ *p53*$^{+/-}$ tumor cells in 3D culture 6 days after lentiviral transduction. Representative images and quantification of the percentage of Ki67-positive cells (n = 3). Scale bar, 20 μm.

(D) Primary *BlgCre Brca1*$^{fl/fl}$ *p53*$^{+/-}$ mouse mammary tumor cells were transduced with either lentiviral shScr and empty expression vectors (shScr), shLyn and empty expression vectors (shLyn), or shLyn and expression vectors carrying either an shLyn-resistant form (indicated by an asterisk) of wild-type LYNA (shLyn + LYNA*WT) or a kinase-dead LYNA mutant (shLyn + LYNA*KD). LYN protein levels determined by western blot 6 days after transduction. The graph shows cell number assessed at day 5 of culture relative to shScr cells.

(E) HCC1937 cells were transduced with control (shScr) or Lyn knockdown (shLyn1 and shLyn2) lentiviruses and tested for LYN expression levels by western blot after 6 days.

(F) shScr-, shLyn1-, or shLyn2-transduced HCC1937 cells were seeded at low density in adherent conditions. Viable cell density was determined after 7 days as in (A). Representative images show tumor cell colonies at day 7 of culture.

(G) *BRCA1* mutant PDX-derived cells (BCM 3887) were transduced with control (shScr) or *LYN* knockdown (shLyn1 and shLyn2) lentiviruses and tested for cell viability after 10–12 days of culture in 3D on Matrigel.

(H) Primary mouse *BlgCre Brca1*$^{fl/fl}$ *p53*$^{+/-}$ mammary tumor cells were transduced with pHIV-RFP-Tet repressor and pSEW-GFP-TO-H1 (carrying either shScr or shLyn) lentiviruses. *Lyn* levels were determined in cells transduced with either inducible shScr or shLyn and in either the presence or the absence of doxycycline (DOX) by qRT-PCR relative to shScr cells without DOX.

(I) 250,000 inducible shScr- or shLyn-transduced cells were orthotopically injected into the fourth right mammary fat pad of nude mice. These were randomized to DOX treatment or normal diet, and tumor growth was monitored. Tumor volumes were calculated from caliper measurements of tumor width and length. Tumor growth curves (mean ± SEM) and representative images of endpoint tumors are shown.

Blots are representative of three independent experiments. Unless otherwise stated, quantitation is shown as mean and SD (n = 3; for PDX cell experiments n = 3 cell isolations from 3 PDX implants in 3 mice; two-tailed unpaired t tests), except for gene expression analysis by quantitative real-time RT-PCR (mean ± 95% confidence intervals; significance of real-time RT-PCR data was determined from confidence intervals; n = 3 independent experiments for each of 3 technical replicates per sample) (Cumming et al., 2007). *p < 0.05; **p < 0.01; ***p < 0.001. See also Figure S3.

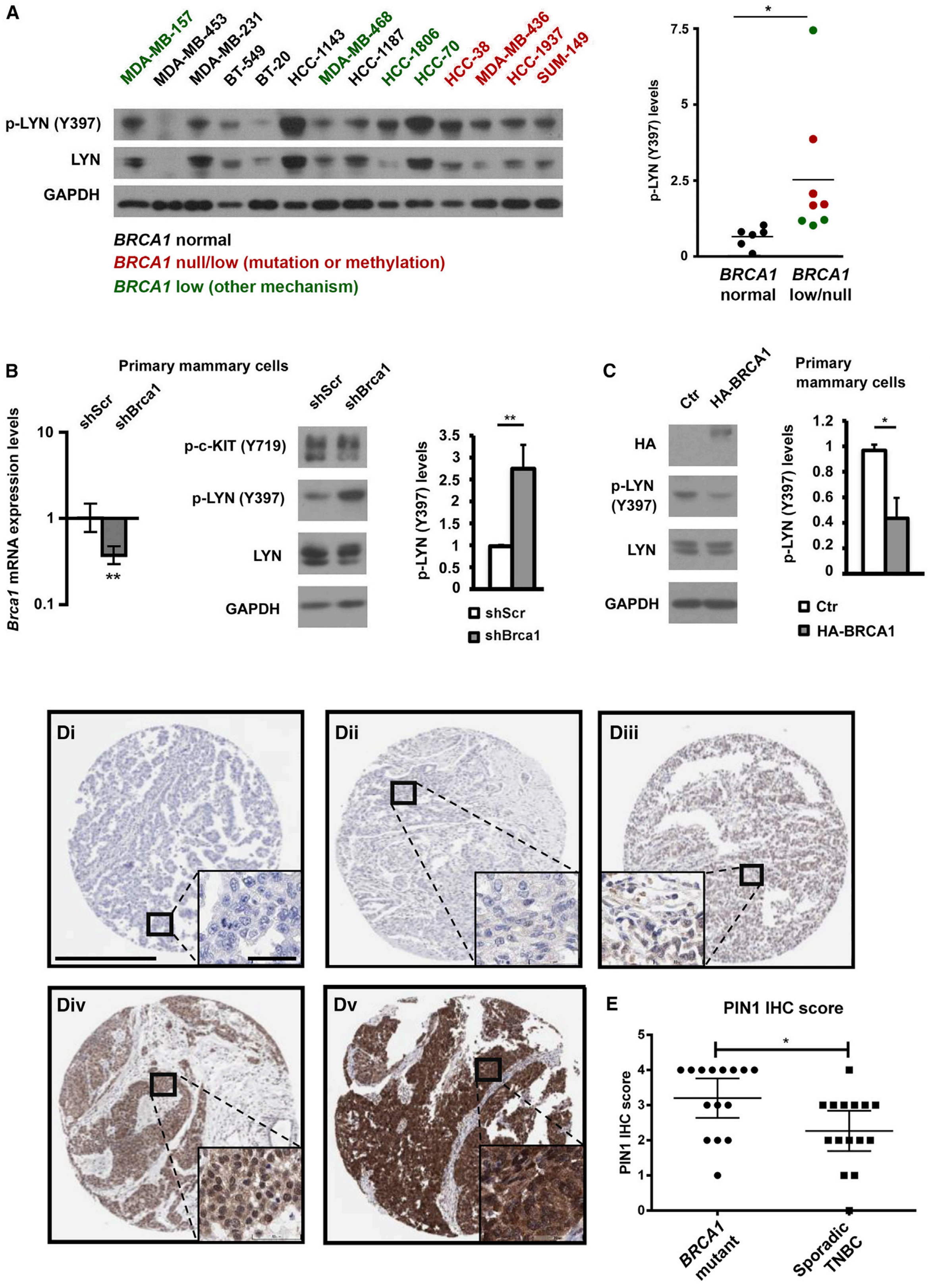

(legend on next page)

investigated their expression patterns in sporadic TCGA breast cancer cases and, consistent with our hypothesis, observed an inverse correlation between BRCA1 and PIN1 expression levels (Figure S4E).

To demonstrate a direct functional link between PIN1 expression and LYN activity, we knocked down PIN1 in primary mouse *Brca1* null cells (Figure 5A) and cells from a *BRCA1* mutant human breast cancer cell line (HCC1937) (Figure 5B) and the *BRCA1* mutant PDX (Figure 5C). In all cases, knockdown of PIN1 decreased active LYN phosphorylation and cell survival, mimicking the effect of LYN knockdown, but it did not change c-KIT phosphorylation.

To elucidate the relationship of LYN phosphorylation, PIN1, and BRCA1, we silenced *PIN1* in a broader panel of TNBC cell lines. In HCC38, MDA-MB-436, HCC1395, and MDA-MB-468 cells (*BRCA1* defective), *PIN1* knockdown resulted in decreased LYN Y397 phosphorylation (Figures S4F–S4J). In HCC1187 cells (*BRCA1* wild-type), *PIN1* knockdown did not affect phosphorylation (Figure S4J); in this line, LYN was still regulated by c-KIT (Figure S3A).

Co-immunoprecipitation (coIP) demonstrated that in mouse *Brca1* null tumor cells, PIN1 interacted with LYN (Figure 5D). Furthermore, generation of mutants in putative PIN1 consensus target sequences (Figure 5E) showed that proline-to-isoleucine mutation of either residue 197 or both 197 and 229 resulted in a significant increase in inhibitory LYN phosphorylation at the Y508 site (Figure 5F).

We next assessed whether the PIN1-LYN regulatory mechanism is likely to be more widely applicable than just to *BRCA1* breast cancer. We therefore knocked down PIN1 in *BRCA2* null mammary epithelial cells and in a panel of *BRCA1* and *BRCA2* null ovarian cancer cells. PIN1 knockdown significantly reduced LYN Y397 phosphorylation in a human *BRCA2* mutant breast cancer cell line (Figure S5A) and in primary mouse *Brca2* null tumor cells (Figure S5B). However, knockdown of *Brca2* in primary normal mouse mammary cells did not alter PIN1 or phospho-LYN levels (Figure S5C). PIN1 knockdown suppressed LYN Y397 phosphorylation in COV 362 cells (*BRCA1* mutant ovarian carcinoma) (Figure S5D) and PEO-1 and PEO-4 cells (*BRCA2* mutant ovarian carcinoma) (Figures S5E and S5F), but not in KURAMOCHI cells (*BRCA2* mutant ovarian carcinoma) (Figure S5G). Therefore, regulation of LYN by PIN1 is a general (but not universal) mechanism, but PIN1 is not regulated by BRCA2. These findings are consistent with transcriptional activity of BRCA1 being involved in PIN1 regulation, as previously shown (MacLachlan et al., 2000).

To further investigate the involvement of specific BRCA1 functional domains in the regulation of the PIN1-LYN axis, and the possibility that different clinically relevant BRCA1 mutants may have different effects on this axis, we re-expressed either the wild-type BRCA1 or clinically relevant BRCA1 missense mutants (C61G in the RING domain, L1407P in the CC motif, and A1708E in the BRCT domain) (Anantha et al., 2017) in the HCC1937 human *BRCA1*-deficient breast cancer cell line. We found that re-expression of both wild-type and C61G mutant BRCA1 resulted in both decreased PIN1 levels and decreased LYN phosphorylation, while expression of the L1407P and A1708E mutations showed no significant differences compared to control *BRCA1* mutant cells (Figure 5G). Therefore, mutation of the N-terminal RING domain (which disrupts binding to BARD1) does not alter the ability of BRCA1 to suppress the PIN1-LYN activation pathway. In contrast, mutation of the coiled-coil domain, affecting PALB2 binding (suggested to be critical for the activation of the BRCA1 transcriptional program, as well as for DNA repair) (Anantha et al., 2017; Gardini et al., 2014), and of the C-terminal BRCT domains, important for interactions with Abraxas, BRIP1, and CtIP (Anantha et al., 2017) and known to be important for BRCA1 transcriptional activity (Hayes et al., 2000; Iofrida et al., 2012), result in elevated levels of PIN1 and LYN activation. These support a model in which the transcriptional activity of BRCA1 is critical in the control of PIN1-LYN pathway activation.

Having established the BRCA1-PIN1-LYN axis, and given the important role of BRCA1 in repair of double-stranded DNA breaks, we examined whether LYN activity could affect the normal mammary cell response to DNA damage. Primary normal mouse mammary epithelial cells expressing LYNA CA were treated with the DNA damaging agent methyl methane sulfonate (MMS), which causes double-stranded breaks. Expression of LYNA CA led to a marked transient increase in Akt phosphorylation, suggesting elevated levels of survival signaling, and a significant reduction in cleaved PARP levels (Figure S6A) and TUNEL staining (Figure S6B), both markers of apoptosis, after MMS treatment relative to control cells. Consistent with this, levels of cleaved caspase-3 were significantly reduced in normal mammary cells expressing LYNA CA, compared to control cells, following treatment with 10 μM cisplatin (Figure S6C) or exposure to 10 Gy of ionizing radiation (Figure S6D).

Figure 4. LYN Activity Is Regulated by BRCA1 via the Prolyl Isomerase PIN1

(A) Protein extracts from TNBC cells with either wild-type *BRCA1* or impaired BRCA1 expression were analyzed for phospho-LYN (p-LYN) (Y397), total LYN, and GAPDH levels by western blot. Scatterplot shows quantification of p-LYN levels normalized to total LYN levels.

(B) Primary mouse mammary organoids were transduced with control (shScr) or *Brca1* knockdown (shBrca1) lentiviruses. Knockdown was assessed by qRT-PCR relative to comparator shScr cells (left). shScr and shBrca1 cells were assessed for levels of phospho-c-KIT (Y719), phospho-LYN (Y397), LYN, and GAPDH by western blot after 4 days (middle).

(C) Western blot analysis and quantitation of LYN autophosphorylation levels in primary mouse mammary organoids transduced with control (Ctr) or HA-tagged BRCA1 (HA BRCA1) expression lentiviruses.

(D) Examples of PIN1 immunohistochemistry scores in breast cancer TMAs: (i) 0, (ii) 1, (iii) 2, (iv) 3, and (v) 4. DAB staining of PIN1, and blue counterstaining of nuclei. Scale bar in main panels, 500 μm; scale bar in inset, 50 μm.

(E) Quantitation of PIN1 scoring in *BRCA1* mutant and sporadic TNBC TMAs.

Blots in (B) and (C) are representative of three independent experiments. Quantitation is shown as mean and SD (n = 3; two-tailed unpaired t tests) except for gene expression analysis by quantitative real-time RT-PCR (mean ± 95% confidence intervals; significance of real-time RT-PCR data was determined from confidence intervals; n = 3 independent experiments for each of 3 technical replicates per sample) (Cumming et al., 2007). $^{*}p < 0.05$; $^{**}p < 0.01$. See also Figure S4.

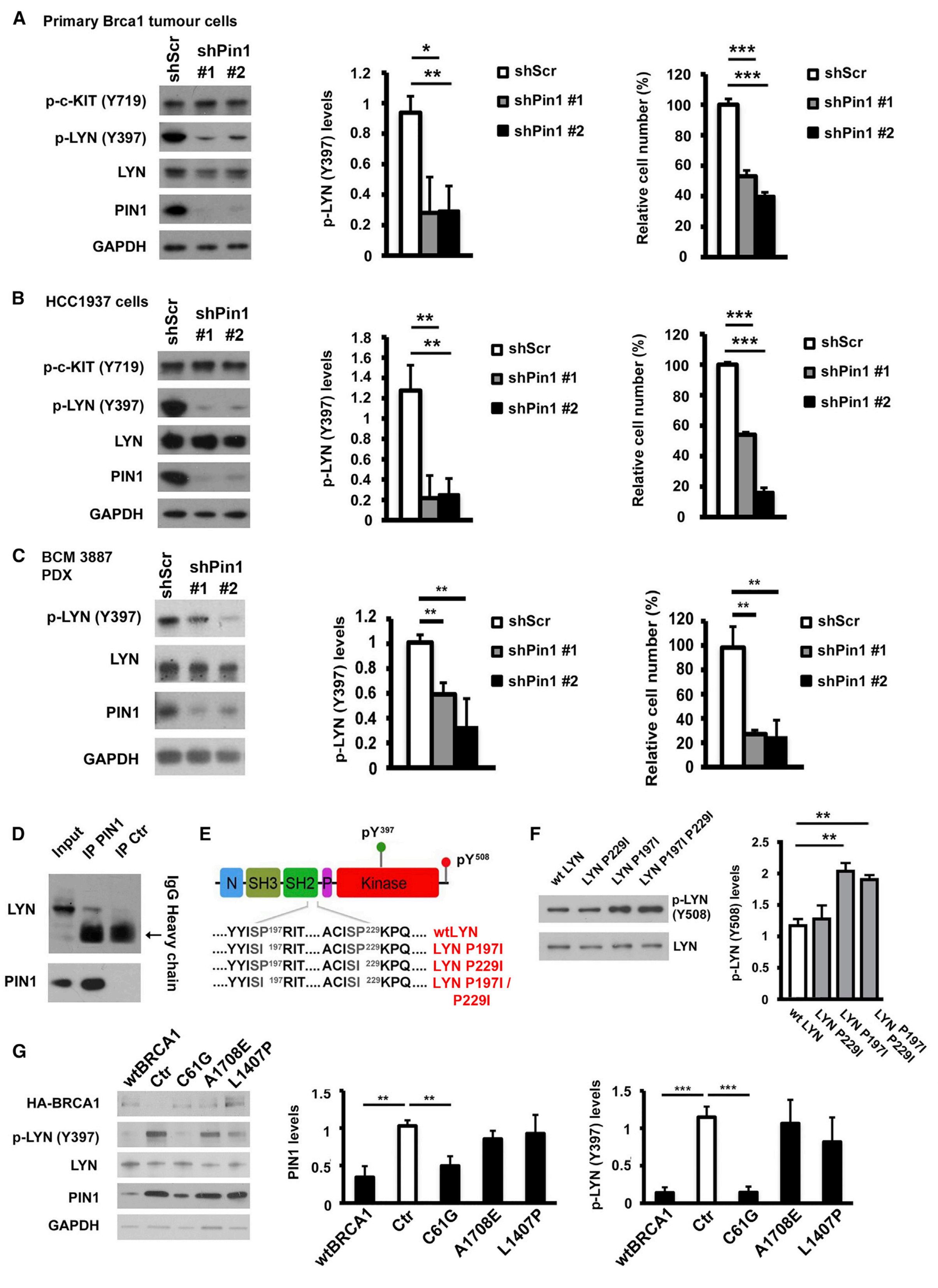

(legend on next page)

LYNA Drives Breast Tumor Cell Aggressiveness

We next asked whether the two LYN isoforms, LYNA and LYNB (shown in detail in Figure S7A), play different roles in breast cancer biology, independent of the BRCA1-PIN1-LYN axis. First, we transiently expressed GFP-tagged variants of LYNA and LYNB in MDA-MB-231 cells. After 48 hr, cells were fixed, counterstained with DAPI, and analyzed by confocal microscopy. Both LYNA and LYNB were predominantly membrane localized, with additional foci of intracellular staining, under these conditions (Figure S7B).

Next, we used a *LYNA*-specific shRNA to knock down *LYNA* expression in MDA-MB-231 cells. shLynA cells displayed an approximately 60% reduction in LYNA protein levels compared to control (shScr) cells (Figure 6A). *LYNA* knockdown resulted in an overall decrease in cell proliferation (Figure 6A) and a strong reduction in cell migration and invasion *in vitro* (Figure 6B). To exclude the possibility that the impaired growth, migration, and invasion of shLynA knockdown cells was due to a reduction in total LYN levels, rather than depletion of the LYNA form, and to determine the specific contribution of each LYN variant to the malignant behavior of the cells, we used a knockdown and reconstitution approach. Total *LYN* was knocked down in MDA-MB-231 cells, and then either a *LYNA* or a *LYNB* variant (*LYNA** or *LYNB**) not targetable by shLyn was re-expressed. We assessed cell growth and the ability of the cells to migrate and invade relative to control cells. Total *LYN* knockdown led to a decrease in cell growth, but this could be rescued by either LYNA* or LYNB* (Figure 6C), indicating that these two distinct LYN isoforms can compensate for each other in promoting tumor cell growth. *LYN* knockdown significantly reduced the ability of the cells to migrate and invade; this could be rescued by LYNA*; however, LYNB* was unable to do so (Figure 6D). Therefore, while both LYN isoforms promoted tumor cell growth, only LYNA drove aggressive behavior in these cells.

To determine whether LYNA and LYNB may associate with different protein partners, and whether this might explain their different effects on migration and invasion, we carried out a mass spectrometry analysis of proteins that interact with the two isoforms. LYN was knocked down in MDA-MB-231 cells, and then either LYNA* or LYNB* was re-expressed. We also expressed a LYNA* variant, LYNA^{*Y32F} (Figure S7C). Y32 is located within the 21-amino acid segment present in LYNA and has been reported as being regulated by epidermal growth factor (EGF) signaling (Huang et al., 2013); if phosphorylation of this tyrosine was required for the differential behavior of LYNA compared to LYNB, then we would predict LYNA^{*Y32F} would behave like LYNB. Cultures were established in duplicate, and one set was treated with EGF before lysis (Huang et al., 2013). LYN was immunoprecipitated from these eight conditions (LYN KD, LYNA*, LYNB*, and LYNA^{*Y32F}; all ±EGF), and lysates were analyzed by tandem mass tagging. The full results and differentially enriched proteins are provided in Table S1. There was little difference between the proteins that co-immunoprecipitated with LYNA* and LYNA^{*Y32F}, arguing against the hypothesis that LYNA^{*Y32F} was like LYNB (Figure S7D). The outcome of the analysis of the LYNA*−EGF, LYNA*+EGF, LYNA^{*Y32F}−EGF, and LYNA^{*Y32F}+EGF pull-downs, four independent cell preparations, was similar. Furthermore, the list of co-immunoprecipitated proteins included eight previously characterized LYN-interacting proteins (ANKRD54, LIMA1, HNRNPK, MYH9, STAT3, PRKDC, EGFR, and HSP90AB1) (Hein et al., 2015; Hornbeck et al., 2015; Huang et al., 2005; Kumar et al., 1998; Mertins et al., 2016; Petschnigg et al., 2014; Taipale et al., 2012; Tauzin et al., 2008; Van Seuningen et al., 1995).

By comparing LYN knockdown samples with LYNA*- and LYNB*-expressing samples, several proteins were identified that were differentially enriched in LYNA* samples. Using a cutoff for analysis of proteins that were enriched >1.2-fold both in the LYNA* pull-down compared to the LYN KD pull-down and in the LYNA* pull-down compared to the LYNB* pull-down, we identified 20 candidate LYNA-interacting proteins. We carried out a gene ontology analysis using DAVID (Huang et al., 2009) of the differentially interacting proteins to begin to understand their functional significance. The list of proteins and the results of this analysis are provided in Table S1. Six proteins (ACTC1, ACTG2, KRT5, LIMA1, MYH3, and TUBA1A) are associated with the cytoskeleton and its regulation, and two proteins (LPXN and TNS1) are associated with integrins and cell adhesion. These findings suggest that LYNA and LYNB may interact differently with cell adhesions and the cytoskeleton, potentially explaining the effects of LYNA on migration and invasion.

Figure 5. LYN Is Activated in BRCA1 Null Cells by the Prolyl Isomerase PIN1

(A–C) Primary cells from *BlgCre Brca1*$^{fl/fl}$ *p53*$^{+/-}$ mouse mammary tumors (A), human HCC1937 BRCA1-deficient breast cancer cells (B), and BRCA1 mutant PDX-derived cells (C) were transduced with control (shScr) or *Pin1* knockdown (shPin1#1 and shPin1#2) lentiviruses and lysed after 72 hr. Protein extracts were assessed for levels of PIN1, LYN, phospho-LYN (Y397), and c-KIT (Y719) (PDX samples were not probed for phospho-KIT). Representative western blots and quantitation of phospho-LYN (Y397) levels are shown. GAPDH was used as loading control. shScr-, shPin1#1-, and shPin1#2-transduced *BlgCre Brca1*$^{fl/fl}$ *p53*$^{+/-}$ tumor cells and HCC1937 cells were also seeded at low density in adherent conditions and stained with crystal violet after 6 days. Cell number was determined by absorbance measurement following solubilization of the dye. PDX-derived transduced cells were cultured for 10–12 days in 3D on Matrigel and then assayed for cell viability.

(D) Protein extracts from primary *BlgCre Brca1*$^{fl/fl}$ *p53*$^{+/-}$ mouse tumor cells transduced with vectors carrying wild-type LYNA were subjected to immunoprecipitation by anti-PIN1 or control (IgG) antibodies. Total extracts (input) and immunoprecipitates (IPs) were probed for PIN1 and LYN by western blot.

(E) Schematic of LYN showing the position of PIN1 consensus recognition sequences and the proline > isoleucine mutants generated.

(F) Representative western blot analysis of LYN phosphorylation levels at the negative regulatory phosphorylation site (Y508) in primary *BlgCre Brca1*$^{fl/fl}$ *p53*$^{+/-}$ transduced with vectors carrying wild-type LYNA or LYNA proline mutants (LYN P229I, LYN P197I, or LYN P197I P229I).

(G) Western blot analysis of LYN autophosphorylation and PIN1 levels in human HCC1937 cells transduced with either control (Ctr) lentivirus or virus-carrying HA-tagged wild-type or mutant BRCA1 (C61G, A1708E, or L1407P).

Blots are representative of three independent experiments. Quantitation is shown as mean and SD (n = 3; two-tailed unpaired t tests). *$p < 0.05$; **$p < 0.01$; ***$p < 0.001$. See also Figures S5 and S6.

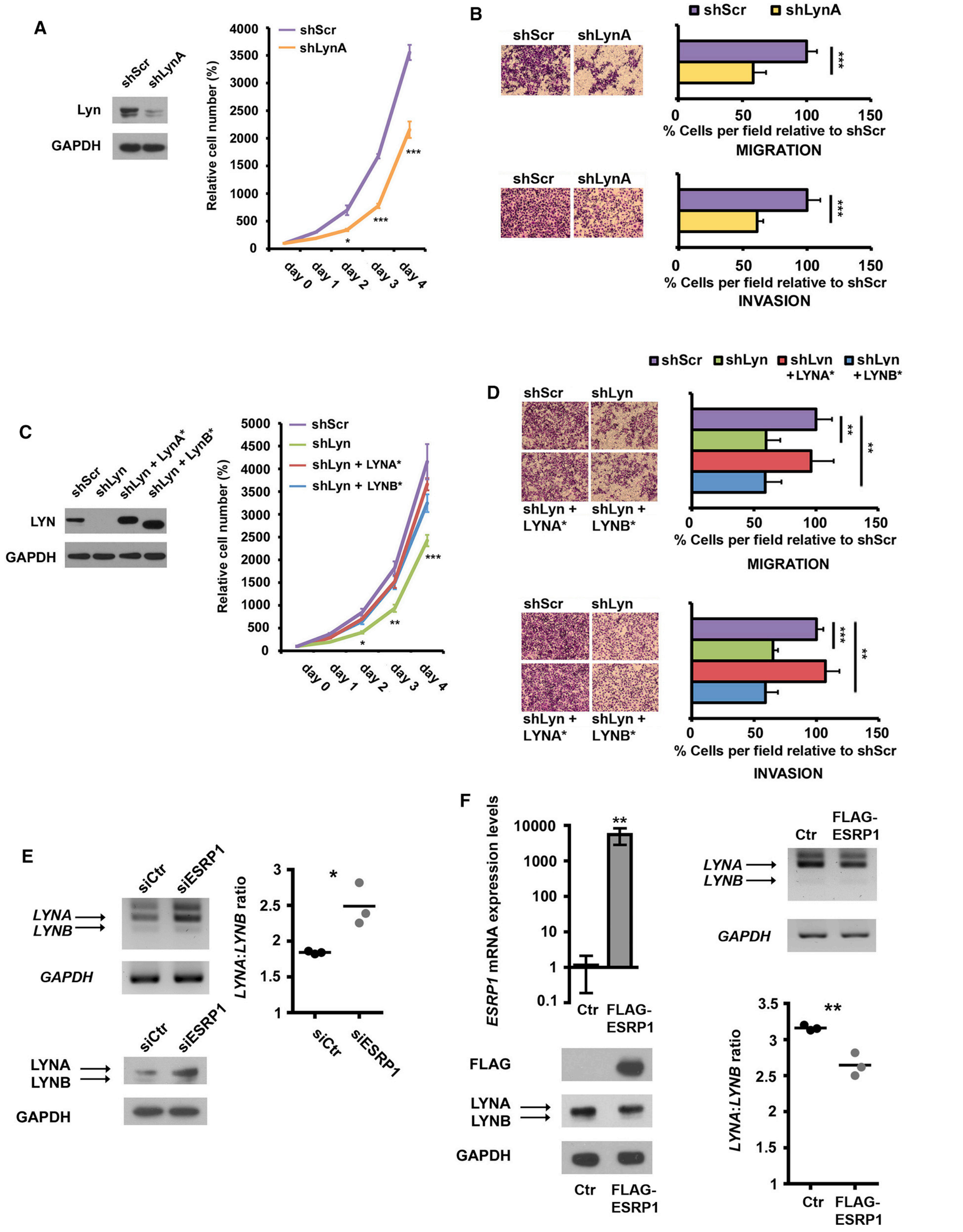

(legend on next page)

LYN Splicing Is Regulated by ESRP1

To determine what might regulate the balance between LYNA and LYNB expression, we first examined Affymetrix Human Exon 1.0ST array gene expression profiles of a breast cancer cohort from Guy's Hospital, London, and the TNBC subset of these cancers. Cohorts were split into high-*LYNA*- and low-*LYNA*-expressing tumors (i.e., above and below median expression of Affymetrix probe 3098998, uniquely targeting the N-terminal region of LYNA), and the expression levels of 270 splicing regulators (the spliceosome) (Table S2) (Papasaikas et al., 2015) were interrogated. We found that in 'all breast cancers' (Figure S8A) and the TNBC subset (Figure S8B), high-*LYNA* tumors had significantly lower spliceosome levels than those of low-*LYNA* tumors, indicating that splicing in general might be compromised. Next, we examined the expression of a splicing regulatory protein (ESRP1/RBM35A) with putative consensus sequences in *LYN* intron 2 (Figure S8C). We found that *ESRP1* levels were lower in high-*LYNA* breast cancers as a whole (Figure S8D) and in the high-*LYNA* TNBC subset (Figure S8E). When *ESRP1* was knocked down in MCF7 cells (Figure S8F), which normally have a *LYNA::B* ratio of <2, the A::B ratio was significantly increased to a mean of 2.5:1 (Figure 6E). Furthermore, when ESRP1 was overexpressed in MDA-MB-231 cells, which normally have a *LYNA::B* ratio of >3, this ratio was significantly reduced (Figure 6F). Therefore, a decrease in the expression of the spliceosome in TNBC, and in particular ESRP1, could result in an increased *LYNA::B* ratio.

Patients with a High Tumor *LYNA::B* Ratio Have Shorter Survival

Because LYNA drives aggressive migratory and invasive properties in breast cancer, we asked whether total *LYNA* expression levels, the relative amounts of *LYNA* and *LYNB*, or the *LYNA::B* ratio might have prognostic potential.

First, we analyzed the relative expression of the *LYNA* and *LYNB* isoforms in samples of human normal mammary tissue, as well as triple-negative (TN) and ER+/PR+ primary breast cancer. The ratio of *LYNA* to *LYNB* transcripts was close to 1 in the normal samples, but *LYNA* was preferentially expressed in TNBC (Figure S9A). No significant difference in relative *LYNA*::*LYNB* expression was observed in ER+/PR+ tumors compared to normal samples (Figure S9A). Similar results were observed in a small panel of human breast cancer cell lines (basal ER− MDA-MB-231, MDA-MB-468, and HCC1143 and luminal ER+ MCF7) (Figure S9B). However, while we could be confident that the tumor samples and cell lines in this analysis were predominantly composed of tumor cells, the normal tissue samples had not been purified and likely contained a mixture of normal epithelial cell populations and non-epithelial cells. Therefore, for a more accurate assessment of the *LYNA::B* ratio in normal human tissue, we used established flow cytometry protocols to purify the basal, luminal progenitor, luminal ER+ differentiated, and stromal cell populations from reduction mammoplasty samples from four individuals (Figures 7A–7C; Figure S10). Analysis of *LYNA::B* demonstrated that the luminal progenitor population had a significantly higher ratio compared with the other populations and that the *LYNA::B* ratio in normal cells was in a similar range to that of the tumor samples.

To expand our analysis, we investigated a panel of breast cancer cell lines (Heiser et al., 2012) and the Guy's Hospital TNBC-enriched breast cancer cohort (Gazinska et al., 2013) for the expression of the *LYNA* isoform using the Affymetrix probe 3098998. The *LYNA* sequence was significantly more highly expressed in basal and claudin-low cell lines than in luminal cell lines (Figure S9C), and in the Guy's dataset, it was more highly expressed in tumors classified by PAM50 (Parker et al., 2009) as basal (Figure S9D) or by immunohistochemistry as TNBC (Figure S9E).

Next, we interrogated *LYNA* and *LYNB* expression in TCGA breast cancer RNA sequencing (RNA-seq) data. Consistent with the microarray-based results, *LYNA* was expressed more highly in TNBC than in non-TNBC ($p = 6.528e-18$, Wilcoxon rank-sum test). Moreover, *LYNB* was higher in TNBC than non-TNBC ($p = 1.554e-20$, Wilcoxon rank-sum test), although it showed overall lower expression levels than *LYNA* ($p = 2.23e-3$, Wilcoxon rank-sum test, for TNBC; $p = 3.354e-22$, Wilcoxon rank-sum tests, for non-TNBC) (Figure 7D). There was no difference in *LYNA::B* ratio for normal breast tissue, TNBC, and non-TNBC in the TCGA dataset (Figure 7E).

When we investigated the distributions of *LYNA::B* ratios across all tumors, we noted that while most sample ratios were in the range seen in the purified normal breast cells (Figure 7C),

Figure 6. LYNA Drives Migration and Invasion in Breast Cancer Cells

(A) Western blot of LYN protein levels and growth of control (shScr) and *LYNA* knockdown (shLynA) MDA-MB-231 cells relative to shScr cells at day 0.

(B) Migration and invasion of shScr- and shLynA-MDA-MB-231 cells, assessed by transwell assay. Representative images show endpoint assays, and quantification of results compares the percentages of cells per field to shScr cells.

(C) Total *LYN* knockdown and *LYNA* or *LYNB* reconstitution using shLyn-resistant forms (*LYNA/B**) in MDA-MB-231 cells. Western blot shows *LYN* knockdown cells forced to express either LYNA or LYNB and growth relative to shScr cells at day 0.

(D) Migration and invasion of control MDA-MB-231 (shScr) cells, LYN-depleted (shLyn) cells, cells expressing LYNA only (shLyn + LYNA*), or cells expressing LYNB only (shLyn + LYNB*). Representative images show endpoint assays, and quantification of results compares the percentages of cells per field to shScr cells.

(E) siControl (siCtr) and siESRP1 MCF7 cells were analyzed for *LYNA*::*LYNB* transcript ratio and LYN protein levels by semiquantitative RT-PCR (top, with quantitation) and western blot (bottom), respectively.

(F) MDA-MB-231 cells were transduced with control (Ctr) or FLAG-tagged ESRP1 (FLAG-ESRP1) lentiviruses. ESRP1 overexpression was assessed by qRT-PCR (fold expression over comparator Ctr cells; upper left panel). Ctr and FLAG-ESRP1 cells were analyzed for *LYNA:LYNB* transcript ratio by semiquantitative RT-PCR (bottom panels), and LYN and ESRP1 protein levels were analyzed by western blot (upper right panel).

Blots are representative of three independent experiments. Quantitation is shown as mean and SD (n = 3; two-tailed unpaired t tests), except for gene expression analysis by quantitative real-time RT-PCR (mean ± 95% confidence intervals; significance of real-time RT-PCR data was determined from confidence intervals; n = 3 independent experiments for each of 3 technical replicates per sample) (Cumming et al., 2007). *$p < 0.05$; **$p < 0.01$; ***$p < 0.001$. See also Figures S7 and S8.

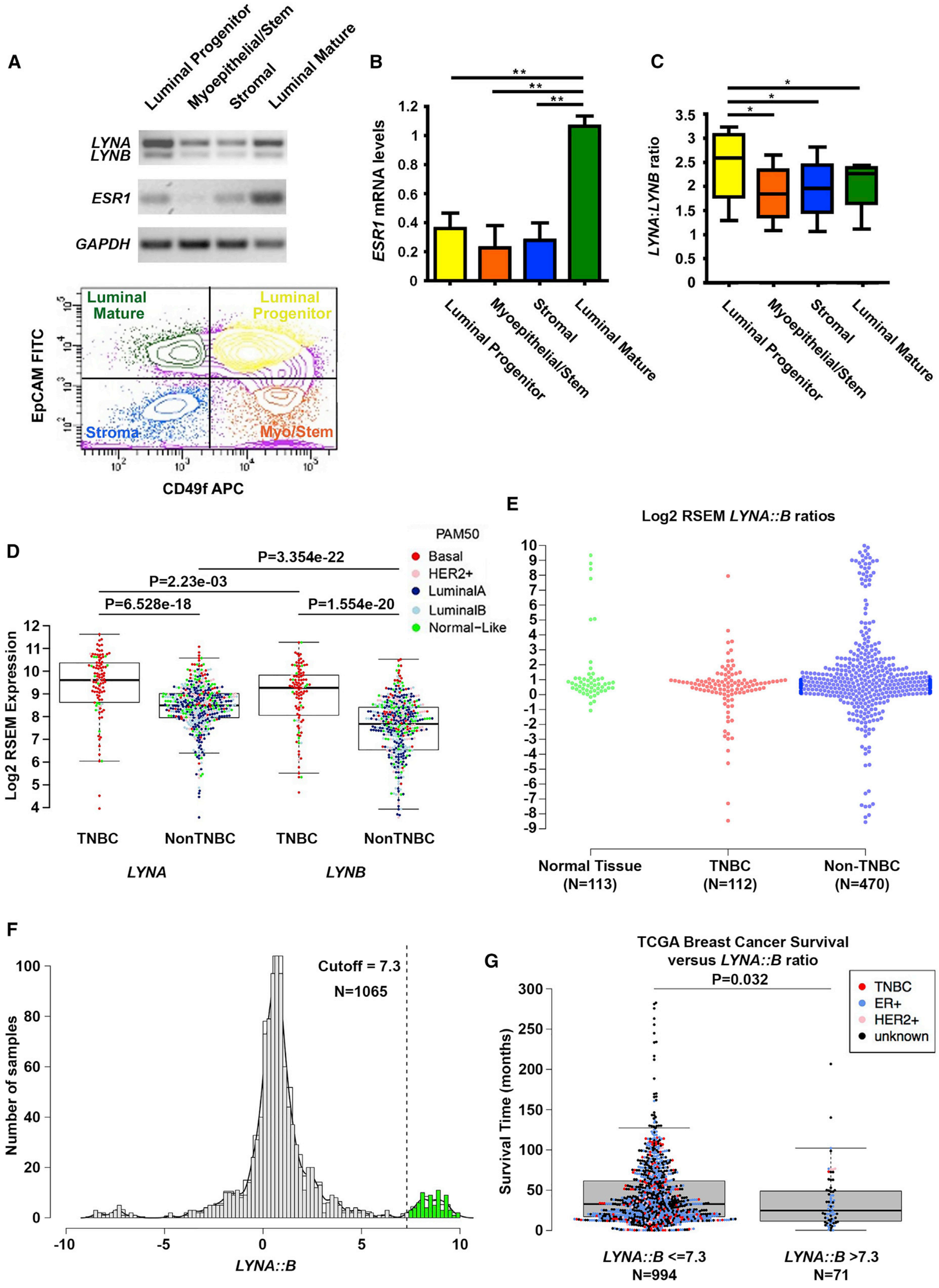

(legend on next page)

there was a distinct population of breast cancers with a log2 RSEM expression ratio of >7 (Figure 7F). When this population was compared for time to breast cancer-specific death with the remaining TCGA breast cancer cases, it had a shorter median time for survival (p = 0.032 for >7.3) (Figure 7G).

DISCUSSION

Although it has been previously reported that LYN is one of the most highly expressed SFKs in the normal mammary gland (Bach et al., 2017; Kendrick et al., 2008; Smart et al., 2011), its function in this tissue has not previously been investigated. LYN associates with c-KIT in hematopoietic cells and participates in numerous SCF-induced responses by promoting either positive or negative downstream signaling, depending on cell type and context (Shivakrupa and Linnekin, 2005). Our results demonstrate that LYN is activated by c-KIT and is critical for SCF:c-KIT-dependent phosphorylation of AKT in mammary progenitors. However, given that LYN has been implicated in other signaling pathways promoting cell survival and proliferation (Shivakrupa and Linnekin, 2005), it cannot be ruled out that additional pathways in mammary progenitors may be regulated by LYN.

c-KIT+/ER− mammary luminal progenitors are considered the cell of origin of BRCA1-mutated and sporadic TNBC (Lim et al., 2009; Molyneux et al., 2010). Although *c-Kit* is highly expressed in *Brca1* mutant mammary tumors (Regan et al., 2012; Smart et al., 2011), as well as in a subset of breast cancers within the TNBC group (Jansson et al., 2014), targeting this receptor has not been an effective therapeutic approach (Yardley et al., 2009). Our findings may at least partly explain why these trials have failed. Although carriers of *BRCA1* germline mutations have an 80% lifetime risk of breast cancer, such cases make a small contribution to breast cancer in the general population. However, *BRCA1* was found to be silenced through promoter methylation in 14% of sporadic basal-like and 11% of non-basal-like breast cancers, while in two special subtypes of TNBC, medullary and metaplastic breast cancer, promoter methylation was found in >60% of cases (Badve et al., 2011; Turner et al., 2007). Furthermore, *BRCA1* mRNA expression was two-fold lower in TNBC compared to matched controls, and this was suggested to depend on upregulation of ID4, a negative regulator of BRCA1 transcription (Turner et al., 2007). BRCA1 levels can also be suppressed by other epigenetic mechanisms, such as activity of miRNAs (Garcia et al., 2011; Li et al., 2013). Therefore, activation of the PIN1-LYN axis by BRCA1 downregulation is more widely applicable than to BRCA1 germline mutation carriers alone.

PIN1 can be aberrantly activated in human cancers by various mechanisms, including changes in transcription, translation, and/or post-translational modifications (Zhou and Lu, 2016). In addition to being a target for BRCA1 transcriptional activity, PIN1 is a direct transcriptional target of E2F (Ryo et al., 2002). PIN1 mRNA stability is also inhibited by miRNAs, while the phosphorylation and/or sumoylation status of specific PIN1 residues has been reported to be critical for PIN1 substrate binding and/or catalytic activity (Zhou and Lu, 2016).

PIN1 specifically catalyzes *cis-trans* proline isomerization within phosphorylated Ser/Thr-Pro motifs with important effects on phosphorylation-dependent signaling. Numerous oncogenes and tumor suppressors are directly regulated by PIN1 (Zhou and Lu, 2016), and here we show that PIN1 is an important contributor to LYN hyperactivation in BRCA1 mutant tumor cells. Consistent with PIN1 substrates typically containing one or few target motifs, LYN has only two putative PIN1 consensus sites (Ser^{196}-Pro^{197} and Ser^{228}-Pro^{229}). LYN phosphorylation at Ser^{196} is only predicted, but phosphorylation at Ser^{228} has been previously observed during cell-cycle progression (Daub et al., 2008), although the specific kinase or kinases involved are still unknown. These two sites are located in the SH2 domain and in the SH2-Kinase domain linker segment, respectively, which are involved in intra- and/or intermolecular interactions critical for the regulation of the open-closed LYN conformation, suggesting that local structural changes upon proline isomerization are likely to affect LYN activation status. Our findings suggest that regulation of LYN by PIN1 is a widely applicable mechanism of regulation of this SFK but that SRC is not a target of PIN1 (Figure S4B); whether other SFKs are PIN1 targets remains to be investigated.

The link between BRCA1 loss of function and LYN activation and the activation by LYN of signaling pathways that promote cell survival, growth, and invasion are important findings. In normal cells, the absence of functional BRCA1 results in genomic instability, which leads to p53 activation, followed by cell-cycle arrest and apoptosis (Roy et al., 2011), implying that additional molecular alterations are required for *BRCA1* mutant

Figure 7. The LYNA::LYNB Isoform Ratio Is Prognostic in Breast Cancer

(A) Representative semiquantitative RT-PCR expression analysis of *ESR1* and *LYN* levels in breast cells from reduction mammoplasty tissue separated into stromal cells, basal cells, luminal progenitors, and mature luminal populations (Iriondo et al., 2015). Plot is representative of outcomes of four independent sorts.

(B) Quantitation of *ESR1* expression levels, confirming *ESR1* is most highly expressed in the mature luminal population (mean + SD relative to mature luminal cells; n = 3 independent cell preparations; unpaired two-tailed t test; $^{**}p < 0.01$).

(C) Quantitation of the relative *LYNA::B* ratio (n = 4 independent cell preparations; paired two-tailed t test; $^{*}p < 0.05$) in breast cell populations.

(D) Expression of *LYNA* and *LYNB* isoforms in The Cancer Genome Atlas (TCGA) breast cancer data split by immunohistochemistry (IHC)-defined TNBC (n = 112) and non-TNBC (n = 470) tumors. Tumors are also color-coded based on their PAM50 molecular subtype.

(E) For each sample shown in (D), the *LYNA::B* ratio was established based on log2 expression of *LYNA* over *LYNB*. The distribution of *LYNA::B* ratios among samples derived from normal breast tissue (data also from TCGA), TNBC and non-TNBC were comparable when tested by Wilcoxon rank-sum test.

(F) Density distributions of *LYNA::B* log2 RSEM expression ratios in the TCGA breast cancer dataset. A *LYNA::B* ratio cutoff of 7.3 is indicated.

(G) Breast cancer-specific survival of patients based on *LYNA::B* isoform expression ratio from TCGA data in patient groups dichotomized at the 7.3 ratio boundary. The IHC phenotype of each tumor (where known) is indicated by the color of each data point. A high *LYNA::B* ratio selects patients with a shorter survival time independent of the breast cancer subtype (Wilcoxon rank-sum test).

See also Figures S9 and S10.

cells to survive and undergo malignant transformation. Not surprisingly, *TP53* mutations are frequently present in *BRCA1*-associated mammary tumors (Roy et al., 2011). As LYN hyperactivation suppressed cell death induced by DNA damage, aberrant LYN activation following *BRCA1* loss could facilitate neoplastic progression, allowing BRCA1 loss-of-function cells to survive long enough to accumulate *TP53* genetic alterations. Furthermore, activation of AKT downstream of LYN has been linked to ubiquitination and degradation of the p53 protein (Dos Santos et al., 2013; Iqbal et al., 2010), and this would enable functional suppression of the p53 pathway in BRCA1 mutant cells before genetic pathway suppression. There is some evidence that LYN is generally anti-apoptotic (Aira et al., 2018), and this warrants further investigation in breast cancer.

Alternative splicing is a critical post-transcriptional regulatory mechanism for many cancer-associated genes (Bonomi et al., 2013). LYN kinase exists as two isoforms, full-length LYN (LYNA) and $LYN^{\Delta 25-45}$ (LYNB), differing by a 21-amino acid insert found in the unique NH2-terminal domain (Alvarez-Errico et al., 2010). We have found that in breast epithelial cells, the balance between these transcripts is modulated by the splicing factor ESRP1. LYN has not been found among the ESRP1-regulated alternative spliced genes resulting from previous analyses (Shapiro et al., 2011; Warzecha et al., 2009, 2010), most likely due to the lack of representative probe sets in the array platforms used in those studies. Nevertheless, like LYN, known ESRP1 target genes play a role in cell motility, cell adhesion, and/or epithelial-mesenchymal transition (Shapiro et al., 2011; Warzecha et al., 2009, 2010), indicating that co-regulation by ESRP1 of splicing of transcripts for proteins that may function, together with LYN, in a pro-migratory and invasive pathway in TNBC cells.

We find that patients with breast cancer with a high *LYNA::LYNB* ratio have a shorter time to breast cancer death. Biologically, this clinical phenotype could be a result of *LYNA* conferring migratory and invasive properties on breast cancer cells. How alteration of the LYNA::LYNB ratio can generate signal outputs leading to cancer cell aggressiveness remains to be fully defined. Previous analysis of LYNA and LYNB function in mast cells revealed the two isoforms associate differentially with phosphoproteins (Alvarez-Errico et al., 2010), indicating that the 21-amino acid sequence governs protein interactions. Moreover, LYNA was more potent than LYNB in activating Phospholipase C gamma (PLCγ) and downstream Ca^{2+} signaling (Alvarez-Errico et al., 2010). In addition, unlike LYNB, LYNA kinase activity can be enhanced through phosphorylation by EGFR at a specific tyrosine residue (Y32) within the 21-amino acid insert (Huang et al., 2013). However, in an analysis of proteins differentially interacting with the LYN isoforms, we saw little effect of either EGF stimulation or Y32F mutation in the 21-amino acid insert. We did find that LYNA interacted more strongly with proteins associated with the cytoskeleton, integrins, and cell adhesion, pointing to differential effects of LYNA and LYNB on migration and invasion. This warrants further work.

Identification of patients who will respond to targeted, novel, or repurposed therapies remains a major goal of clinical research. Our findings demonstrate that patients with BRCA1 dysfunction or with a high *LYNA::B* isoform ratio would be particularly likely to benefit from specific therapies targeting LYN kinase. Furthermore, our findings on the key dual mechanisms of LYN regulation, combined with knowledge of LYN interaction partners, will enable rational design of new compounds to specifically block the oncogenic signaling driven by LYN without the need to directly target the kinase domain, increasing treatment specificity and reducing the likelihood of off-target effects.

STAR★METHODS

Detailed methods are provided in the online version of this paper and include the following:

- KEY RESOURCES TABLE
- CONTACT FOR REAGENT AND RESOURCE SHARING
- EXPERIMENTAL MODEL AND SUBJECT DETAILS
  - Animals
  - Human Tissue
  - Cell lines
- METHOD DETAILS
  - Isolation of and culture of normal mouse mammary epithelial cells
  - Preparation and flow cytometric separation of normal breast cells from reduction mammoplasty
  - Primary tumor cell isolation and culture
  - Protein extraction and western blot analysis
  - Immunoprecipitation (IP) LYN kinase assay
  - LYN-PIN1 co-immunoprecipitation
  - Gene expression analysis
  - Cell viability and growth assays
  - Cell migration and invasion assay
  - siRNA Transfection
  - Lentiviral vectors and cell transduction
  - Generation and expression of LYN-GFP fusion proteins
  - *In vitro* and *in vivo* Dasatinib treatment
  - *In vivo* conditional Lyn knockdown
  - Immunofluorescence staining
  - Phospho-Histone H3 immunohistochemical staining
  - PIN1 Immunohistochemistry and analysis of BRCA1 tumor TMA
  - Isoform specific expression analysis by Affymetrix
  - Isoform specific expression analysis by RNaseq
  - LYN pull-down for Tandem Mass Tag (TMT) labeling
  - TMT Labeling and High pH reversed-phase chromatography
  - Nano-LC Mass Spectrometry
  - TMT Data Analysis
- QUANTIFICATION AND STATISTICAL ANALYSIS

SUPPLEMENTAL INFORMATION

Supplemental Information includes ten figures and three tables and can be found with this article online at https://doi.org/10.1016/j.celrep.2018.11.103.

ACKNOWLEDGMENTS

This work was funded by Breast Cancer Now (2012MayPR076) and Breast Cancer Research Aid. G.T. is supported by Breast Cancer Now and the Wales Cancer Research Centre. M.J.S. and H.K. are supported by Cancer Research

UK (CRUK/A15938), the European Cancer Stem Cell Research Institute, and Cardiff University. H.M. and A.G. are funded by Breast Cancer Now (2012MaySF122 and 2013NovPhD184). A.G. is supported by Cancer Research UK. C.K. and N.E.B. are funded by Breast Cancer Now. M.d.M.V. thanks MINECO/FEDER, EU (SAF2017/84934/R) and the Centro Excelencia Severo Ochoa (SEV-2016-0644)-Agencia Estatal de Investigación. The authors acknowledge technical assistance from Danny Hawker, Ben Egan, and Liam Roche-Lamerton; bioinformatics support from Aisyah Mohd Noor; and assistance with tissue microarray (TMA) staining from Victoria Bingham and Stephen McQuaid. The authors also thank Professor Klaus Holzmann for the gift of the ESRP1 plasmid, Professor Vladimir Buchman (Cardiff University) for the gift of pEGFP-N3, and Professor Andrew Tutt for support and advice.

AUTHOR CONTRIBUTIONS

Conceptualization, M.J.S., N.E.B., and G.T.; Methodology, G.T. and M.d.M.V.; Investigation, G.T., C.K., J.C., I.A.-R., and H.K.; Writing – Original Draft, G.T., A.G., and M.J.S.; Writing – Review & Editing, G.T., A.G., M.d.M.V., and M.J.S.; Formal analysis, H.M. and A.G.; Funding acquisition, N.E.B. and M.J.S.

DECLARATION OF INTERESTS

The authors declare no competing interests.

Received: July 7, 2016
Revised: October 27, 2018
Accepted: November 29, 2018
Published: December 26, 2018

REFERENCES

Aira, L.E., Villa, E., Colosetti, P., Gamas, P., Signetti, L., Obba, S., Proics, E., Gautier, F., Bailly-Maitre, B., Jacquel, A., et al. (2018). The oncogenic tyrosine kinase Lyn impairs the pro-apoptotic function of Bim. Oncogene *37*, 2122–2136.

Alvarez-Errico, D., Yamashita, Y., Suzuki, R., Odom, S., Furumoto, Y., Yamashita, T., and Rivera, J. (2010). Functional analysis of Lyn kinase A and B isoforms reveals redundant and distinct roles in Fc epsilon RI-dependent mast cell activation. J. Immunol. *184*, 5000–5008.

Anantha, R.W., Simhadri, S., Foo, T.K., Miao, S., Liu, J., Shen, Z., Ganesan, S., and Xia, B. (2017). Functional and mutational landscapes of BRCA1 for homology-directed repair and therapy resistance. eLife *6*, e21350.

Bach, K., Pensa, S., Grzelak, M., Hadfield, J., Adams, D.J., Marioni, J.C., and Khaled, W.T. (2017). Differentiation dynamics of mammary epithelial cells revealed by single-cell RNA sequencing. Nat. Commun. *8*, 2128.

Badve, S., Dabbs, D.J., Schnitt, S.J., Baehner, F.L., Decker, T., Eusebi, V., Fox, S.B., Ichihara, S., Jacquemier, J., Lakhani, S.R., et al. (2011). Basal-like and triple-negative breast cancers: a critical review with an emphasis on the implications for pathologists and oncologists. Mod. Pathol. *24*, 157–167.

Bates, D., Maechler, M., Bolker, B., and Walker, S. (2015). Fitting linear mixed-effects models using lme4. J. Stat. Softw. *67*, 1–48.

Bonomi, S., Gallo, S., Catillo, M., Pignataro, D., Biamonti, G., and Ghigna, C. (2013). Oncogenic alternative splicing switches: role in cancer progression and prospects for therapy. Int. J. Cell Biol. *2013*, 962038.

Brasó-Maristany, F., Filosto, S., Catchpole, S., Marlow, R., Quist, J., Francesch-Domenech, E., Plumb, D.A., Zakka, L., Gazinska, P., Liccardi, G., et al. (2016). PIM1 kinase regulates cell death, tumor growth and chemotherapy response in triple-negative breast cancer. Nat. Med. *22*, 1303–1313.

Buckley, N.E., Conlon, S.J., Jirstrom, K., Kay, E.W., Crawford, N.T., O'Grady, A., Sheehan, K., Mc Dade, S.S., Wang, C.W., McCance, D.J., et al. (2011). The DeltaNp63 proteins are key allies of BRCA1 in the prevention of basal-like breast cancer. Cancer Res. *71*, 1933–1944.

Buckley, N.E., Haddock, P., De Matos Simoes, R., Parkes, E., Irwin, G., Emmert-Streib, F., McQuaid, S., Kennedy, R., and Mullan, P. (2016). A BRCA1 deficient, NFκB driven immune signal predicts good outcome in triple negative breast cancer. Oncotarget *7*, 19884–19896.

Choi, Y.L., Bocanegra, M., Kwon, M.J., Shin, Y.K., Nam, S.J., Yang, J.H., Kao, J., Godwin, A.K., and Pollack, J.R. (2010). LYN is a mediator of epithelial-mesenchymal transition and a target of dasatinib in breast cancer. Cancer Res. *70*, 2296–2306.

Cumming, G., Fidler, F., and Vaux, D.L. (2007). Error bars in experimental biology. J. Cell Biol. *177*, 7–11.

Daub, H., Olsen, J.V., Bairlein, M., Gnad, F., Oppermann, F.S., Körner, R., Greff, Z., Kéri, G., Stemmann, O., and Mann, M. (2008). Kinase-selective enrichment enables quantitative phosphoproteomics of the kinome across the cell cycle. Mol. Cell *31*, 438–448.

Dos Santos, C., McDonald, T., Ho, Y.W., Liu, H., Lin, A., Forman, S.J., Kuo, Y.H., and Bhatia, R. (2013). The Src and c-Kit kinase inhibitor dasatinib enhances p53-mediated targeting of human acute myeloid leukemia stem cells by chemotherapeutic agents. Blood *122*, 1900–1913.

Garcia, A.I., Buisson, M., Bertrand, P., Rimokh, R., Rouleau, E., Lopez, B.S., Lidereau, R., Mikaélian, I., and Mazoyer, S. (2011). Down-regulation of BRCA1 expression by miR-146a and miR-146b-5p in triple negative sporadic breast cancers. EMBO Mol. Med. *3*, 279–290.

Gardini, A., Baillat, D., Cesaroni, M., and Shiekhattar, R. (2014). Genome-wide analysis reveals a role for BRCA1 and PALB2 in transcriptional co-activation. EMBO J. *33*, 890–905.

Gazinska, P., Grigoriadis, A., Brown, J.P., Millis, R.R., Mera, A., Gillett, C.E., Holmberg, L.H., Tutt, A.N., and Pinder, S.E. (2013). Comparison of basal-like triple-negative breast cancer defined by morphology, immunohistochemistry and transcriptional profiles. Mod. Pathol. *26*, 955–966.

Gong, C., Fujino, K., Monteiro, L.J., Gomes, A.R., Drost, R., Davidson-Smith, H., Takeda, S., Khoo, U.S., Jonkers, J., Sproul, D., and Lam, E.W. (2015). FOXA1 repression is associated with loss of BRCA1 and increased promoter methylation and chromatin silencing in breast cancer. Oncogene *34*, 5012–5024.

Hay, T., Matthews, J.R., Pietzka, L., Lau, A., Cranston, A., Nygren, A.O., Douglas-Jones, A., Smith, G.C., Martin, N.M., O'Connor, M., and Clarke, A.R. (2009). Poly(ADP-ribose) polymerase-1 inhibitor treatment regresses autochthonous Brca2/p53-mutant mammary tumors *in vivo* and delays tumor relapse in combination with carboplatin. Cancer Res. *69*, 3850–3855.

Hayes, F., Cayanan, C., Barillà, D., and Monteiro, A.N. (2000). Functional assay for BRCA1: mutagenesis of the COOH-terminal region reveals critical residues for transcription activation. Cancer Res. *60*, 2411–2418.

Hein, M.Y., Hubner, N.C., Poser, I., Cox, J., Nagaraj, N., Toyoda, Y., Gak, I.A., Weisswange, I., Mansfeld, J., Buchholz, F., et al. (2015). A human interactome in three quantitative dimensions organized by stoichiometries and abundances. Cell *163*, 712–723.

Heiser, L.M., Sadanandam, A., Kuo, W.L., Benz, S.C., Goldstein, T.C., Ng, S., Gibb, W.J., Wang, N.J., Ziyad, S., Tong, F., et al. (2012). Subtype and pathway specific responses to anticancer compounds in breast cancer. Proc. Natl. Acad. Sci. USA *109*, 2724–2729.

Hochgräfe, F., Zhang, L., O'Toole, S.A., Browne, B.C., Pinese, M., Porta Cubas, A., Lehrbach, G.M., Croucher, D.R., Rickwood, D., Boulghourjian, A., et al. (2010). Tyrosine phosphorylation profiling reveals the signaling network characteristics of Basal breast cancer cells. Cancer Res. *70*, 9391–9401.

Hornbeck, P.V., Zhang, B., Murray, B., Kornhauser, J.M., Latham, V., and Skrzypek, E. (2015). PhosphoSitePlus, 2014: mutations, PTMs and recalibrations. Nucleic Acids Res. *43*, D512–D520.

Huang, H.D., Lee, T.Y., Tzeng, S.W., and Horng, J.T. (2005). KinasePhos: a web tool for identifying protein kinase-specific phosphorylation sites. Nucleic Acids Res. *33*, W226–W299.

Huang, D.W., Sherman, B.T., and Lempicki, R.A. (2009). Bioinformatics enrichment tools: paths toward the comprehensive functional analysis of large gene lists. Nucleic Acids Res. *37*, 1–13.

Huang, T.H., Huo, L., Wang, Y.N., Xia, W., Wei, Y., Chang, S.S., Chang, W.C., Fang, Y.F., Chen, C.T., Lang, J.Y., et al. (2013). Epidermal growth factor receptor potentiates MCM7-mediated DNA replication through tyrosine phosphorylation of Lyn kinase in human cancers. Cancer Cell *23*, 796–810.

Iofrida, C., Melissari, E., Mariotti, V., Guglielmi, C., Guidugli, L., Caligo, M.A., and Pellegrini, S. (2012). Effects on human transcriptome of mutated BRCA1 BRCT domain: a microarray study. BMC Cancer *12*, 207.

Iqbal, M.S., Tsuyama, N., Obata, M., and Ishikawa, H. (2010). A novel signaling pathway associated with Lyn, PI 3-kinase and Akt supports the proliferation of myeloma cells. Biochem. Biophys. Res. Commun. *392*, 415–420.

Iriondo, O., Rábano, M., Domenici, G., Carlevaris, O., López-Ruiz, J.A., Zabalza, I., Berra, E., and Vivanco, Md. (2015). Distinct breast cancer stem/progenitor cell populations require either HIF1α or loss of PHD3 to expand under hypoxic conditions. Oncotarget *6*, 31721–31739.

Jansson, S., Bendahl, P.O., Grabau, D.A., Falck, A.K., Fernö, M., Aaltonen, K., and Rydén, L. (2014). The three receptor tyrosine kinases c-KIT, VEGFR2 and PDGFRα, closely spaced at 4q12, show increased protein expression in triple-negative breast cancer. PLoS ONE *9*, e102176.

Kendrick, H., Regan, J.L., Magnay, F.A., Grigoriadis, A., Mitsopoulos, C., Zvelebil, M., and Smalley, M.J. (2008). Transcriptome analysis of mammary epithelial subpopulations identifies novel determinants of lineage commitment and cell fate. BMC Genomics *9*, 591.

Kumar, S., Pandey, P., Bharti, A., Jin, S., Weichselbaum, R., Weaver, D., Kufe, D., and Kharbanda, S. (1998). Regulation of DNA-dependent protein kinase by the Lyn tyrosine kinase. J. Biol. Chem. *273*, 25654–25658.

Leontieva, O.V., and Ionov, Y. (2009). RNA-binding motif protein 35A is a novel tumor suppressor for colorectal cancer. Cell Cycle *8*, 490–497.

Li, D., Ilnytskyy, Y., Kovalchuk, A., Khachigian, L.M., Bronson, R.T., Wang, B., and Kovalchuk, O. (2013). Crucial role for early growth response-1 in the transcriptional regulation of miR-20b in breast cancer. Oncotarget *4*, 1373–1387.

Liedtke, C., Mazouni, C., Hess, K.R., André, F., Tordai, A., Mejia, J.A., Symmans, W.F., Gonzalez-Angulo, A.M., Hennessy, B., Green, M., et al. (2008). Response to neoadjuvant therapy and long-term survival in patients with triple-negative breast cancer. J. Clin. Oncol. *26*, 1275–1281.

Lim, E., Vaillant, F., Wu, D., Forrest, N.C., Pal, B., Hart, A.H., Asselin-Labat, M.L., Gyorki, D.E., Ward, T., Partanen, A., et al.; kConFab (2009). Aberrant luminal progenitors as the candidate target population for basal tumor development in BRCA1 mutation carriers. Nat. Med. *15*, 907–913.

MacLachlan, T.K., Somasundaram, K., Sgagias, M., Shifman, Y., Muschel, R.J., Cowan, K.H., and El-Deiry, W.S. (2000). BRCA1 effects on the cell cycle and the DNA damage response are linked to altered gene expression. J. Biol. Chem. *275*, 2777–2785.

Mertins, P., Mani, D.R., Ruggles, K.V., Gillette, M.A., Clauser, K.R., Wang, P., Wang, X., Qiao, J.W., Cao, S., Petralia, F., et al.; NCI CPTAC (2016). Proteogenomics connects somatic mutations to signalling in breast cancer. Nature *534*, 55–62.

Molyneux, G., Geyer, F.C., Magnay, F.A., McCarthy, A., Kendrick, H., Natrajan, R., Mackay, A., Grigoriadis, A., Tutt, A., Ashworth, A., et al. (2010). BRCA1 basal-like breast cancers originate from luminal epithelial progenitors and not from basal stem cells. Cell Stem Cell *7*, 403–417.

Papasaikas, P., Tejedor, J.R., Vigevani, L., and Valcárcel, J. (2015). Functional splicing network reveals extensive regulatory potential of the core spliceosomal machinery. Mol. Cell *57*, 7–22.

Parker, J.S., Mullins, M., Cheang, M.C., Leung, S., Voduc, D., Vickery, T., Davies, S., Fauron, C., He, X., Hu, Z., et al. (2009). Supervised risk predictor of breast cancer based on intrinsic subtypes. J. Clin. Oncol. *27*, 1160–1167.

Perou, C.M., Sørlie, T., Eisen, M.B., van de Rijn, M., Jeffrey, S.S., Rees, C.A., Pollack, J.R., Ross, D.T., Johnsen, H., Akslen, L.A., et al. (2000). Molecular portraits of human breast tumours. Nature *406*, 747–752.

Petschnigg, J., Groisman, B., Kotlyar, M., Taipale, M., Zheng, Y., Kurat, C.F., Sayad, A., Sierra, J.R., Mattiazzi Usaj, M., Snider, J., et al. (2014). The mammalian-membrane two-hybrid assay (MaMTH) for probing membrane-protein interactions in human cells. Nat. Methods *11*, 585–592.

Regan, J.L., Kendrick, H., Magnay, F.A., Vafaizadeh, V., Groner, B., and Smalley, M.J. (2012). c-Kit is required for growth and survival of the cells of origin of Brca1-mutation-associated breast cancer. Oncogene *31*, 869–883.

Roskoski, R., Jr. (2005). Signaling by Kit protein-tyrosine kinase—the stem cell factor receptor. Biochem. Biophys. Res. Commun. *337*, 1–13.

Roy, R., Chun, J., and Powell, S.N. (2011). BRCA1 and BRCA2: different roles in a common pathway of genome protection. Nat. Rev. Cancer *12*, 68–78.

Ryo, A., Liou, Y.C., Wulf, G., Nakamura, M., Lee, S.W., and Lu, K.P. (2002). PIN1 is an E2F target gene essential for Neu/Ras-induced transformation of mammary epithelial cells. Mol. Cell. Biol. *22*, 5281–5295.

Schwarz, L.J., Fox, E.M., Balko, J.M., Garrett, J.T., Kuba, M.G., Estrada, M.V., González-Angulo, A.M., Mills, G.B., Red-Brewer, M., Mayer, I.A., et al. (2014). LYN-activating mutations mediate antiestrogen resistance in estrogen receptor-positive breast cancer. J. Clin. Invest. *124*, 5490–5502.

Shapiro, I.M., Cheng, A.W., Flytzanis, N.C., Balsamo, M., Condeelis, J.S., Oktay, M.H., Burge, C.B., and Gertler, F.B. (2011). An EMT-driven alternative splicing program occurs in human breast cancer and modulates cellular phenotype. PLoS Genet. *7*, e1002218.

Shivakrupa, R., and Linnekin, D. (2005). Lyn contributes to regulation of multiple Kit-dependent signaling pathways in murine bone marrow mast cells. Cell. Signal. *17*, 103–109.

Smalley, M.J. (2010). Isolation, culture and analysis of mouse mammary epithelial cells. Methods Mol. Biol. *633*, 139–170.

Smalley, M.J., Kendrick, H., Sheridan, J.M., Regan, J.L., Prater, M.D., Lindeman, G.J., Watson, C.J., Visvader, J.E., and Stingl, J. (2012). Isolation of mouse mammary epithelial subpopulations: a comparison of leading methods. J. Mammary Gland Biol. Neoplasia *17*, 91–97.

Smart, C.E., Wronski, A., French, J.D., Edwards, S.L., Asselin-Labat, M.L., Waddell, N., Peters, K., Brewster, B.L., Brooks, K., Simpson, K., et al. (2011). Analysis of Brca1-deficient mouse mammary glands reveals reciprocal regulation of Brca1 and c-kit. Oncogene *30*, 1597–1607.

Taipale, M., Krykbaeva, I., Koeva, M., Kayatekin, C., Westover, K.D., Karras, G.I., and Lindquist, S. (2012). Quantitative analysis of HSP90-client interactions reveals principles of substrate recognition. Cell *150*, 987–1001.

Tauzin, S., Ding, H., Khatib, K., Ahmad, I., Burdevet, D., van Echten-Deckert, G., Lindquist, J.A., Schraven, B., Din, N.U., Borisch, B., and Hoessli, D.C. (2008). Oncogenic association of the Cbp/PAG adaptor protein with the Lyn tyrosine kinase in human B-NHL rafts. Blood *111*, 2310–2320.

Tornillo, G., Elia, A.R., Castellano, I., Spadaro, M., Bernabei, P., Bisaro, B., Camacho-Leal, Mdel.P., Pincini, A., Provero, P., Sapino, A., et al. (2013). p130Cas alters the differentiation potential of mammary luminal progenitors by deregulating c-Kit activity. Stem Cells *31*, 1422–1433.

Turner, N.C., Reis-Filho, J.S., Russell, A.M., Springall, R.J., Ryder, K., Steele, D., Savage, K., Gillett, C.E., Schmitt, F.C., Ashworth, A., and Tutt, A.N. (2007). BRCA1 dysfunction in sporadic basal-like breast cancer. Oncogene *26*, 2126–2132.

Van Seuningen, I., Ostrowski, J., and Bomsztyk, K. (1995). Description of an IL-1-responsive kinase that phosphorylates the K protein. Enhancement of phosphorylation by selective DNA and RNA motifs. Biochemistry *34*, 5644–5650.

Warzecha, C.C., Shen, S., Xing, Y., and Carstens, R.P. (2009). The epithelial splicing factors ESRP1 and ESRP2 positively and negatively regulate diverse types of alternative splicing events. RNA Biol. *6*, 546–562.

Warzecha, C.C., Jiang, P., Amirikian, K., Dittmar, K.A., Lu, H., Shen, S., Guo, W., Xing, Y., and Carstens, R.P. (2010). An ESRP-regulated splicing

programme is abrogated during the epithelial-mesenchymal transition. EMBO J. *29*, 3286–3300.

Wu, J., Meng, F., Kong, L.Y., Peng, Z., Ying, Y., Bornmann, W.G., Darnay, B.G., Lamothe, B., Sun, H., Talpaz, M., and Donato, N.J. (2008). Association between imatinib-resistant BCR-ABL mutation-negative leukemia and persistent activation of LYN kinase. J. Natl. Cancer Inst. *100*, 926–939.

Yardley, D.A., Burris, H.A., 3rd, Markus, T., Spigel, D.R., Greco, F.A., Mainwaring, M., Waterhouse, D.M., Webb, C.D., and Hainsworth, J.D. (2009). Phase II trial of docetaxal plus imatinib mesylate in the treatment of patients with metastatic breast cancer. Clin. Breast Cancer *9*, 237–242.

Zhang, X., Claerhout, S., Prat, A., Dobrolecki, L.E., Petrovic, I., Lai, Q., Landis, M.D., Wiechmann, L., Schiff, R., Giuliano, M., et al. (2013). A renewable tissue resource of phenotypically stable, biologically and ethnically diverse, patient-derived human breast cancer xenograft models. Cancer Res. *73*, 4885–4897.

Zhou, X.Z., and Lu, K.P. (2016). The isomerase PIN1 controls numerous cancer-driving pathways and is a unique drug target. Nat. Rev. Cancer *16*, 463–478.

STAR★METHODS

KEY RESOURCES TABLE

REAGENT or RESOURCE	SOURCE	IDENTIFIER
Antibodies		
CD24-FITC	BD PharMingen, Oxford, UK	Cat# 553261, FITC-conjugated rat monoclonal clone M1/69
Sca-1-PE	BD PharMingen, Oxford, UK	Cat# 553336, PE-conjugated rat monoclonal clone E13-161.7
Sca-1-APC	eBioscience, Thermo Fisher Scientific, Life Technologies, Paisley, UK	Cat# 17-5981-81, APC-conjugated rat monoclonal clone D7
CD45-PE-Cy7	BD PharMingen, Oxford, UK	Cat# 552848, PE-Cy7-conjugated rat monoclonal clone 30-F11
Anti-Human CD326 (EpCAM)	StemCell Technologies Inc.	Cat# 60147FI, FITC-conjugated mouse monoclonal clone 5E11.3.1
CD49f-APC	eBioscience, Thermo Fisher Scientific, Life Technologies, Paisley, UK	Cat# 17-0495-80, APC-conjugated rat monoclonal clone GoH3
IgG isotype control	eBioscience, Thermo Fisher Scientific, Life Technologies, Paisley, UK	Cat# 14-4031-82, clone eB149/10H5, functional grade, purified
c-KIT	eBioscience, Thermo Fisher Scientific, Life Technologies, Paisley, UK	Cat# 14-1172-82, rat monoclonal ACK2, functional grade, purified
c-KIT	Cell Signaling Technology, Leiden, the Netherlands	Cat# 3074, rabbit monoclonal clone D13A2
phospho-Y719 c-KIT	Cell Signaling Technology, Leiden, the Netherlands	Cat# 3391, rabbit polyclonal
AKT	Cell Signaling Technology, Leiden, the Netherlands	Cat# 4685, rabbit monoclonal clone 11E7
phospho-S473 AKT	Cell Signaling Technology, Leiden, the Netherlands	Cat# 9271, rabbit polyclonal
phospho-T202/Y204 MAPK (ERK1/2)	Cell Signaling Technology, Leiden, the Netherlands	Cat# 9101, rabbit polyclonal
phospho-Y1007/1008 JAK2	Cell Signaling Technology, Leiden, the Netherlands	Cat# 3771, rabbit polyclonal
phospho-Y705 STAT3	Cell Signaling Technology, Leiden, the Netherlands	Cat# 9131, rabbit polyclonal
LYN	Abcam, Cambridge, UK	Cat# ab1890, mouse monoclonal clone LYN-01
LYN	Santa Cruz, Heidelberg, Germany	Cat# sc-15, rabbit polyclonal
phospho-Y396 LYN / phosphor-Y418 SRC-family kinases This antibody was originally sold as anti-pY396 then its description was changed to anti-pY418; as a result we changed to ab226778 below. We carried out a number of optimization experiments and found no difference between their reactivity in our samples.	Abcam, Cambridge, UK	Cat# ab40660, rabbit monoclonal EP503Y
phospho-Y396 LYN	Abcam, Cambridge, UK	Cat# ab226778, rabbit polyclonal
phospho-Y507 (human)/Y508 (mouse|) LYN	Abcam, Cambridge, UK	Cat# ab2731, rabbit polyclonal
PIN1	Santa Cruz, Heidelberg, Germany	Cat# sc-15340, rabbit polyclonal
PIN1	Santa Cruz, Heidelberg, Germany	Cat# sc-46660, mouse monoclonal clone G-8
ESRP1	GeneTex, Insight Biotechnology, London, UK	Cat# GTX131373, rabbit polyclonal

(*Continued on next page*)

Continued

REAGENT or RESOURCE	SOURCE	IDENTIFIER
BRCA1	Sigma, Poole, Dorset, UK	Cat# HPA034966, rabbit polyclonal
α6 Integrin	eBioscience, Thermo Fisher Scientific, Life Technologies, Paisley, UK	Cat# 14-0495-82, rat monoclonal clone GoH3
cleaved PARP1	Cell Signaling Technology, Leiden, the Netherlands	Cat# 9544, rabbit polyclonal
Ki-67	Vector Laboratories, Orton Southgate, Peterborough, UK This antibody is no longer available from this company.	Cat# VP-K452, mouse monoclonal clone MM1
phospho-Histone H3	Cell Signaling Technology, Leiden, the Netherlands	Cat# 9701, rabbit polyclonal
DYKDDDDK FLAG Tag	Cell Signaling Technology, Leiden, the Netherlands	Cat# 2368, rabbit polyclonal
HA	Cell Signaling Technology, Leiden, the Netherlands	Cat# 3724, rabbit monoclonal clone C29F4
GAPDH	Merck Millipore, Watford, Hertfordshire, UK	Cat# CB1001, mouse monoclonal clone 6C5
α-tubulin	Sigma, Poole, Dorset, UK	Cat# T9026, mouse monoclonal clone DM1A
HRP-conjugated anti-mouse IgG	Sigma, Poole, Dorset, UK	Cat# A4416, polyclonal
HRP-conjugated anti-rabbit IgG	Sigma, Poole, Dorset, UK	Cat# A6154, polyclonal
Alexa Fluor® 488-conjugated anti-mouse IgG	Life Technologies, Molecular Probes, Paisley, UK	Cat# R37114, polyclonal
Alexa Fluor® 488-conjugated anti-rat IgG	Life Technologies, Molecular Probes, Paisley, UK	Cat# A-11006, polyclonal
Biological Samples		
RNA samples from human breast tumor tissue and reduction mammoplasties	Breast Cancer Now Tissue Bank	Anonymized
Normal breast tissue from women (n = 4; aged 15, 24, 35, 39 years) undergoing reduction mammoplasty with no previous history of breast cancer	Cruz Roja, Clínica Indautxu	Anonymized
Human breast cancer patient-derived xenograft (PDX) BCM 3887	Baylor College of Medicine; an MTA may be required for distribution of this material	Zhang et al., 2013
Human BRCA1 breast cancer tissue microarray	Northern Ireland Biobank via Niamh Buckley, Queen's University Belfast; an MTA may be required for distribution of this material	N/A
Chemicals, Peptides, and Recombinant Proteins		
Dasatinib	Selleckchem, Stratech, Newmarket, Suffolk, UK	Cat# S1021
Soluble murine SCF	Peprotech, London, UK	Cat# 250-03
Critical Commercial Assays		
Proteome Profiler Human Phospho-Kinase Array Kit	R&D Systems, Abingdon, Oxford, UK	Cat# ARY003B
ApopTag® Red *In Situ* Apoptosis Detection Kit	Merck Millipore, Watford, Hertfordshire, UK	Cat# S7165
Mouse Tumor Dissociation Kit for GentleMACS	Miltenyi Biotec, Bisley, Surrey, UK	Cat# 130-096-730
Experimental Models: Cell Lines		
COV362	European Collection of Authenticated Cell Cultures (ECACC)	Cat# 07071910
PEO1	European Collection of Authenticated Cell Cultures (ECACC)	Cat# 10032308

(*Continued on next page*)

Continued

REAGENT or RESOURCE	SOURCE	IDENTIFIER
PEO4	European Collection of Authenticated Cell Cultures (ECACC)	Cat# 10032308
KURAMOCHI	Japanese Collection of Research Bioresources Cell Bank (JCRB).	Cat# JCRB0098
MCF-7	American Type Culture Collection (ATCC)	Cat# ATCC HTB-22
MCF10A	American Type Culture Collection (ATCC)	Cat# ATCC CRL-10317
MDA-MB-157	American Type Culture Collection (ATCC)	Cat# ATCC CRL-24
MDA-MB-231	American Type Culture Collection (ATCC)	Cat# ATCC HTB-26
MDA-MB-436	American Type Culture Collection (ATCC)	Cat# ATCC HTB-130
MDA-MB-453	American Type Culture Collection (ATCC)	Cat# ATCC HTB-131
MDA-MB-468	American Type Culture Collection (ATCC)	Cat# ATCC HTB-132
MDA-MB-468 EV (Empty vector)	Niamh Buckley and Paul Mullan, Queens University Belfast; an MTA may be required for distribution of this material	N/A
MDA-MB-468 BR (BRCA1 overexpressing)	Niamh Buckley and Paul Mullan, Queens University Belfast; an MTA may be required for distribution of this material	N/A
BT-20	American Type Culture Collection (ATCC)	Cat# ATCC HTB-19
BT-549	American Type Culture Collection (ATCC)	Cat# ATCC HTB-122
HCC38	American Type Culture Collection (ATCC)	Cat# ATCC CRL-2314
HCC70	American Type Culture Collection (ATCC)	Cat# ATCC CRL-2315
HCC1143	American Type Culture Collection (ATCC)	Cat# ATCC CRL-2321
HCC1187	American Type Culture Collection (ATCC)	Cat# ATCC CRL-2322
HCC1395	American Type Culture Collection (ATCC)	Cat# ATCC CRL-2324
HCC1599	American Type Culture Collection (ATCC)	Cat# ATCC CRL-2331
HCC1806	American Type Culture Collection (ATCC)	Cat# ATCC CRL-2335
HCC1937	American Type Culture Collection (ATCC)	Cat# ATCC CRL-2336
SUM-149	BioIVT, West Sussex, UK	Cat# SUM-149PT
HEK293T	From in-house frozen stocks; also available from American Type Culture Collection (ATCC)	ATCC Cat# CRL-11268
Experimental Models: Organisms/Strains		
10 week old virgin female FVB mice	Charles River, Margate, Kent, UK	FVB/NCrl
Trp53^{tm1Brd} Brca1^{tm1Aash} Tg(LGB-cre)74Acl/J (*BlgCre Brca1$^{fl/fl}$ p53$^{+/-}$*) mice	The Jackson Laboratory, Bar Harbor, Maine, USA	Stock# 012620
BlgCre Brca2$^{fl/fl}$ p53$^{fl/fl}$ mice	In house; an MTA may be required for distribution of this material	Hay et al., 2009
NOD SCID γ mice	Charles River, Margate, Kent, UK	NSG
Oligonucleotides		
See Table S3 for details of oligos used for RT-PCR, site directed mutagenesis, PCR cloning, shRNA and siRNA knockdown, SYBR Green qRTPCR oligos and TAQman qrtPCR assays		
Non-Targeting siRNA Pool #1	Dharmacon, Cambridge, UK	Cat# D-001206-13-05
ON-TARGETplus ESRP1 siRNA	Dharmacon, Cambridge, UK	Cat# L-020672-01-0005
Recombinant DNA		
pEGFP-N3	Prof Vladimir Buchman, Cardiff University	N/A
pENTR^{TM}/H1/TO	Thermo Fisher Scientific, Life Technologies, Paisley, UK	Cat# K4920-00
ESRP1 cDNA	Prof Klaus Holzmann, Institute of Cancer Research, Medical University of Vienna	Leontieva and Ionov, 2009

(*Continued on next page*)

Continued		
REAGENT or RESOURCE	SOURCE	IDENTIFIER
pcDNA^{TM}6/TR (part of T-REx core kit)	Thermo Fisher Scientific, Life Technologies, Paisley, UK	Cat# K102002
pENTR^{TM}/U6 (part of BLOCK-iT U6 RNAi Entry Vector Kit)	Thermo Fisher Scientific, Life Technologies, Paisley, UK	Cat# K4944-00
pHIV-H2BmRFP	Addgene (https://www.addgene.org/)	Cat# 18982
pLKO.1 MISSION TRC shRNA targeting mouse *Brca1*	Sigma, Poole, Dorset, UK	Cat# TRCN0000042559
pLKO.1 MISSION TRC shRNA targeting mouse *Kit*#1	Sigma, Poole, Dorset, UK	Cat# TRCN0000023672
pLKO.1 MISSION TRC shRNA targeting mouse *Kit*#2	Sigma, Poole, Dorset, UK	Cat# TRCN0000023673
pLKO.1 MISSION TRC shRNA targeting human *Lyn*#1	Sigma, Poole, Dorset, UK	Cat# TRCN0000230901
pLKO.1 MISSION TRC shRNA targeting human *Lyn*#2	Sigma, Poole, Dorset, UK	Cat# TRCN0000218210
pLKO.1 MISSION TRC shRNA targeting mouse *Lyn*#1	Sigma, Poole, Dorset, UK	Cat# TRCN0000023666
pLKO.1 MISSION TRC shRNA targeting mouse shLyn#2	Sigma, Poole, Dorset, UK	Cat# TRCN0000023668
pLKO.1 scramble (shScr)	Addgene (https://www.addgene.org/)	Cat# 26701
Gateway modified pWPI	In house; an MTA may be required for distribution of this material; pWPI originally from Tronolabs	Regan et al., 2012
psPAX2	Addgene	Cat# 12260
pMD2.G	Addgene	Cat# 12259
pSEW-GFP-TO-H1	In house; an MTA may be required for distribution of this material	Regan et al., 2012
Software and Algorithms		
Proteome Discoverer software v2.1	Thermo Fisher Scientific, Life Technologies, Paisley, UK	Cat# OPTON-30795
Statistical analysis of tumor growth was conducted using the *glmer* function for generalized linear mixed models from the *lme4* package in R (version 3.2.2)	https://www.r-project.org/	Bates et al., 2015
Other		
Gentle MACS^{TM} Dissociator	Miltenyi Biotec, Bisley, Surrey, UK	Cat# 130-093-235
McIlwain Tissue Chopper	Campden Instruments, Loughborough, Leicestershire, UK	Cat# TC752

CONTACT FOR REAGENT AND RESOURCE SHARING

Further information and requests for resources and reagents should be directed to and will be fulfilled by the Lead Contact, Professor Matt Smalley (SmalleyMJ@cardiff.ac.uk).

EXPERIMENTAL MODEL AND SUBJECT DETAILS

Animals

All animal work was carried out under UK Home Office project and personal licenses following local ethical approval and in accordance with local and national guidelines, including ARRIVE guidelines. Normal primary mammary cells were prepared from fourth mammary fat pads of 10 week-old virgin female FVB mice. The *BlgCre Brca1*$^{fl/fl}$ *p53*$^{+/-}$ and *BlgCre Brca2*$^{fl/fl}$ *p53*$^{fl/fl}$ mice and the tumors they generate have been fully described previously (Hay et al., 2009; Molyneux et al., 2010).

Human Tissue

Normal breast tissue was obtained from women (n = 4; aged 15, 24, 35, 39 years) undergoing reduction mammoplasty with no previous history of breast cancer. Patients provided written informed consent and the procedures were approved by the local Hospital Research Ethics Committee and by the 'Ethics Committee of Clinical Investigation of Euskadi'.

The human breast cancer patient-derived xenograft (PDX) BCM 3887 derived from a patient with a BRCA1 mutation (Zhang et al., 2013) was passaged in NOD *scid* gamma (NGS) mice.

The BRCA1 breast tumor (n = 15) and normal triple negative breast cancer (n = 15) tissue microarray was prepared by the Northern Ireland Biobank under ethical approval number NIB17-0232.

RNA samples from human tumor tissue were obtained from Breast Cancer Now Tissue Bank. Normal tissue samples were from reduction mammoplasties, selected to contain > 50% epithelium. All tumor samples (10 ER+PR+HER2- and 10 Triple Negative) were from primary tumors of no specific type, grade III, from pre-menopausal patients.

Cell lines

Cells were maintained at 37°C in a 5% CO_2 atmosphere with the exception of MDA-MB-157, which were kept in L-15 medium with 10% FBS, streptomycin (100 ug/ml) and penicillin (100 U/ml) in a free gas exchange with atmospheric air.

MCF10A cells were maintained in DMEM/F12 supplemented with 5% horse serum, 20 ng/ml EGF, 10 μg/ml insulin, 1 ng/ml cholera toxin, 100 μg/ml hydrocortisone, 50 U/ml penicillin and 50 μg/ml streptomycin (growth medium). BT-549, KURAMOCHI, MCF-7, MDA-MB-231 and MDA-MB-436 cells were cultured in RPMI 1640 medium with 10% FBS, L-glutamine (4 mM), streptomycin (100 ug/ml) and penicillin (100 U/ml). HCC38, HCC-70, HCC1143, HCC1187, HCC-1395, HCC-1599, HCC1806 and HCC1937 cells were cultured in modified RPMI-1640 medium (ATCC 30-2001) supplemented with 10% FBS, streptomycin (100 ug/ml) and penicillin (100 U/ml). COV-362, MDA-MB-453 and MDA-MB-468 cells were grown in DMEM with 10% FBS, streptomycin (100 ug/ml) and penicillin (100 U/ml). HCC1937 and MDA-MB-468 cells stably overexpressing BRCA1 were previously generated (Buckley et al., 2011) and were grown in the presence of puromycin (1 ug/ml). BT-20 cells were grown in MEM added with 10% FBS, non-essential amino acids (0.1 mM), L-glutamine (2 mM), sodium pyruvate (1 mM), streptomycin (100 ug/ml) and penicillin (100 U/ml). PEO-1 and PEO-4 cells were cultured in RPMI 1640 medium with 10% FBS, L-glutamine (2 mM), sodium pyruvate (2mM) streptomycin (100 ug/ml) and penicillin (100 U/ml). SUM-149 cells were grown in Ham's F-12 medium containing 5% FBS, HEPES (10 mM), insulin (5 ug/ml), hydrocortisone (1 ug/ml), streptomycin (100 ug/ml) and penicillin (100 U/ml). See Key Resources Table for more details.

METHOD DETAILS

Isolation of and culture of normal mouse mammary epithelial cells

All animal work was carried out under UK Home Office project and personal licenses following local ethical approval and in accordance with local and national guidelines, including ARRIVE guidelines.

Single cells were prepared from fourth mammary fat pads of humanely killed 10 week-old virgin female FVB mice. Intramammary lymph nodes were removed prior to tissue collection. Fat pads were finely minced on a McIlwain Tissue Chopper and then digested for 1 hr at 37°C in 3 mg/ml collagenase A / 1.5 mg/ml trypsin (both from Sigma, Poole Dorset, UK) in serum-free L15 medium (ThermoFisher Scientific, Life Technologies, Paisley, UK) with gentle rotation. Tissue fragments ('organoids') released from the fat pad were washed and then incubated for 5 min in Red Blood Cell Lysis buffer (Sigma), washed and then plated for 1 hr at 37°C in DMEM/10%FBS (ThermoFisher) to partially purify fibroblasts by differential attachment. Organoids were then poured off, pelleted, washed twice with versene (ThermoFisher) and then incubated for 15 min in serum-free Joklik's Low Calcium medium (Sigma) at 37°C. They were then pelleted and resuspended in 2mls of 0.25% trypsin / 0.02% EDTA in HBSS (Sigma) and incubated for two min 37°C to release single epithelial cells. 5 ml of 5 μg/ml DNase I (Sigma) in serum-free L15 was then added to digest DNA liberated from any lysed cells. Single epithelial cells were then pelleted and washed in L15/10% FBS (ThermoFisher Scientific, Life Technologies, Paisley, UK) and then resuspended at 10^6 cells/ml in L15/10% FBS (Regan et al., 2012; Smalley, 2010; Smalley et al., 2012).

Cell suspensions were stained with combinations of anti-CD24-FITC (1.0 μg/ml; BD Biosciences, Oxford, UK), anti-CD45-PE-Cy7 (1.0 μg/ml; BD Biosciences), anti-Sca-1-APC (1.0 μg/ml; eBioscience, Hatfield, UK) or anti-Sca-1-PE (1.0 μg/ml; BD Biosciences) antibodies and DAPI. Cells were then sorted on a FACSAria flow cytometer (BD Biosciences) excluding non-single cells by Time-Of-Flight analysis, dead cells by DAPI staining and leukocytes by CD45 staining. Basal mammary epithelial cells were defined as $CD24^{+/Low}$ $Sca\text{-}1^{Negative}$. Luminal ER negative progenitor cells were defined as $CD24^{+/High}$ $Sca\text{-}1^{Negative}$. Luminal ER positive differentiated cells were defined as $CD24^{+/High}$ $Sca\text{-}1^{Positive}$. Cells incubated in non-specific IgG were used to set the limits of negative and positive staining for each antibody (Regan et al., 2012; Smalley, 2010; Smalley et al., 2012).

For 3D cultures, cells were resuspended in complete growth medium (DMEM:F12 with 10% FBS (ThermoFisher Scientific), 5 ug/ml insulin (Sigma, Poole, UK), 10 ng/ml cholera toxin (Sigma) and 10 ng/ml epidermal growth factor) supplemented with 2.5% growth factor reduced Matrigel (BD Biosciences, Oxford, UK) and plated in 96- or 48- well plates onto Matrigel (40 ul or 100 ul per well, respectively). Cultures were maintained at 37 °C in a 5% CO_2/5%O_2 atmosphere in a Galaxy 170R incubator (New Brunswick, Eppendorf, Stevenage, UK). Stimulation with soluble murine SCF (Peprotech, London, UK) (100 ng/ml) and treatment with anti-c-Kit (ACK2) or IgG isotype control antibodies (50 ug/ml) were carried out after starving cells for 12 hr.

Phase-contrast images were taken using a Leica MI6000B microscope (10X PH1 objective) and the LAS AF software.

Preparation and flow cytometric separation of normal breast cells from reduction mammoplasty

Normal breast tissue was obtained from pre-menopausal women undergoing reduction mammoplasty, with no previous history of breast cancer, who gave their informed consent. All samples were confirmed by histopathological examination to be free of malignancy. Immediately upon arrival at the laboratory, breast tissue was cut up manually into small pieces (approximately 0.5 cm cubed). Breast material was incubated in an equal volume of Dulbecco's modified Eagle's medium (DMEM) (GIBCO) supplemented with 5% fetal calf serum (FCS) and collagenase (Type I, Sigma) to a final concentration of 0.2 mg/ml, and digested (while shaking) overnight at 37°C. Following enzyme digestion, breast cells were washed and the organoids separated from any undigested material. The organoids were then isolated from blood cells, fibroblasts, and endothelial cells by sequential filtration and back flushing from 140 and 53 μm pore size polyester monofilament meshes. Organoids were then disaggregated with 0.05% trypsin-EDTA and finally filtered through a 40 μm sieve (BD) to yield a predominantly single cell suspension. Cells were immediately processed for flow cytometric cell sorting on the basis of CD49f, ESA and 7-AAD staining (see Figure 7 and Figure S10) (Iriondo et al., 2015).

For CD49f/ESA staining, FITC-conjugated anti-ESA antibody and APC-conjugated anti-CD49f antibody were used (see Key Resources Table). In all cases, control samples were stained with isotype-matched control antibodies; the viability dye 7-aminoactinomycin D (7AAD) (BD) was used for dead cell exclusion and fluorescence minus one (FMO) controls were used to define the gates (Iriondo et al., 2015). In all cases, cells were analyzed and sorted using a FACSAria (Becton Dickinson) flow cytometer. Data were analyzed using FACSDiva software.

Primary tumor cell isolation and culture

Primary epithelial cells (from three distinct tumors (namely #1, #2, #3) from each mouse model or from three PDX implants) were obtained using the gentle MACS^{TM} Dissociator and Mouse Tumor dissociation kit (Miltenyi Biotec, Bisley, Surrey, UK) following the manufacturer's recommendations using the protocol for 'Dissociation of Tough Tumors' for mouse tumors and the protocol for 'Dissociation of Soft and Medium Tumors' for the PDX. To ensure efficient dissociation volumes of Enzyme D, Enzyme R and Enzyme A were scaled up according to the size of the tumor piece (100 μL, 50 μL and 12.5 μL respectively per each 0.5 cm^3). The optional steps - the short spin for collection of the dissociated material at the bottom of the MACS tube and red blood cell lysis - were included in the procedure.

Mouse cells were cultured in complete growth medium in 2D adherent conditions for expansion or in 3D for functional studies. Cells up to passage 5 were used for all the experiments in this study. Freshly isolated human PDX cells were grown in HuMEC Ready Medium (Thermo Fisher Scientific) in Matrigel in 3D. Cultures were maintained at 37°C in a 5% CO_2/5%O_2 atmosphere in a Galaxy 170R incubator (New Brunswick, Eppendorf).

Protein extraction and western blot analysis

3D cultured primary mammary cells were released from Matrigel using the BD cell recovery solution and lysed in Laemmli buffer. Protein extracts were separated by SDS-PAGE, transferred to PVDF membranes (IPVH00010, Merck Millipore, Hertfordshire, UK) and immunoblotted with antibodies detailed in the Key Resources Table. GAPDH or alpha-tubulin were used as loading controls. Resulting immunocomplexes were detected by HRP-conjugated anti-mouse IgG or anti-rabbit IgG secondary antibodies and enhanced chemiluminescent (ECL) reagents (WBLUF0100, Merck Millipore). Protein extracts (400 ug) from Ctr, BRCA1-, siCtr- and siPin1-MDA-MB-468 cells were processed and analyzed for phosphorylation of LYN (Y397) and SRC (Y419) using the Human Phospho-Kinase Antibody Array (R&D Systems) following the manufacturer's instructions.

Immunoprecipitation (IP) LYN kinase assay

Once recovered from Matrigel, 3D cultured cells were lysed in RIPA buffer (50mM Tris/HCl, pH 7.5, 150mM NaCl, 1% Triton X-100, 1% Na deoxycolate, 0.1% SDS) supplemented with 1mM Na orthovanadate and protease inhibitor-cocktail (Roche, Burgess Hill, West Sussex, UK). After centrifugation (14000 *g* for 10 min at 4°C), supernatants (150 μg of protein per sample) were pre-cleared with protein A-Sepharose beads (GE Healthcare, Cardiff, UK) for 45 min at 4°C prior to incubation with anti-LYN antibodies (rabbit polyclonal sc-15) for 2 hr at 4°C. Immunocomplexes were pulled down after binding to protein A-Sepharose beads (GE Healthcare) for 45 min at 4°C and washed twice with 20 mM HEPES, pH 7.4, 5 mM MgCl2, 3 mM MnCl2 1mM, 1mM Na orthovanadate (kinase buffer). Beads were then resuspended in 50 μL of kinase buffer with 2.75 μg of acid denatured enolase (Sigma), 5-10 μCi of γ^{32}P ATP (PerkinElmer, Seer Green, Buckinghamshire, UK) and 1 μM cold ATP. After a 10 min-incubation at 30°C, the reaction was stopped by adding 13 μL of 10mM ATP, 50 mM EDTA and samples were subjected to SDS-PAGE on a 10% acrylamide gel. Gels were fixed in 10% methanol/ 10% acetic acid solution, then dried and developed by autoradiography. Intensities of bands corresponding to phosphorylated enolase were measured using the ImageJ software.

LYN-PIN1 co-immunoprecipitation

Primary *BlgCre Brca1*$^{fl/fl}$ *p53*$^{+/-}$ mouse tumor cells were collected in cold PBS pH 8.3 buffer with 10 mM EDTA, 0.1% Tween 20, 10 mM Sodium Fluoride, 1 mM Sodium Orthovanadate, 10 mM Sodium Pyrophosphate, 100 mM β-Glycerophosphate, 2 mM PMSF, complete Protease Inhibitors (Roche) and lysed by passing through a 26G needle. After centrifugation (14000 *g* for 15 min at 4°C), supernatants (3-4 mg of protein) were pre-cleared with protein A-Sepharose beads (GE Healthcare) for 45 min at 4°C prior to incubation with anti-Pin1 (rabbit polyclonal (H-123), sc-15340, Santa Cruz) or control (IgG) antibodies overnight at 4°C. After

incubation with protein A-Sepharose beads for 45 min at 4°C, immunoprecipitates were pulled down by centrifugation (900 *g* for 5 min at 4°C), washed five times with lysis buffer and eluted with Laemmli buffer. Samples were then resolved by SDS-PAGE on 10% polyacrylamide gels (15 × 15 cm). Western blot analysis was carried out as described above.

Gene expression analysis

With the exception of purified human primary cell populations (see below), RNA was extracted using the RNeasy Mini Kit (QIAGEN, Manchester, UK) from freshly isolated primary mouse mammary cells and 2D cultured cells. Alternatively, Trizol reagent (ThermoFisher Scientific, Paisley, UK) was used for RNA isolation from 3D cultured cells. cDNA synthesis was carried out using QuantiTect Reverse Transcription Kit (QIAGEN) according to the manufacturer's instructions.

Semiquantitative PCR reactions (28 cycles) were performed using GoTaq® PCR Core System reagents (Promega, Southampton, UK) and up to 120 ng of cDNA as template. Primers are listed in Table S3. PCR products were separated by electrophoresis on a 2% agarose gel with the exception of c-Kit PCR products, which were resolved on a 4% agarose gel.

Quantitative real-time PCR (qPCR) was carried out using TAQMAN (Applied Biosystems, Life Technologies, Paisley, UK) Assays-on-Demand probes or Fast SYBR green Master Mix (Table S3) on freshly isolated RNA. Results were analyzed using the Δ-ΔCt method normalized to β-actin or GAPDH and expressed as relative to a comparator sample.

For normal primary human breast cell populations purified by flow cytometry, RNA was isolated using the Machery-Nagel NucleoSpin RNA, according to instructions of the manufacturer. DNase-treated RNA was used to synthesize cDNA using SuperScript VILO cDNA Synthesis Kit (Invitrogen, 11754050), following the manufacturer's protocol. Semi-cuantitative-PCR was performed using Phusion High-Fidelity DNA Polymerase (Thermofisher Scientific, F530S) and Deoxynucleotide (dNTP) Mix, PCR Reagents (Sigma, D7295) on a MyCycler thermal cycler (Bio-Rad). 10 ng of cDNA was used as template and amplified using the following conditions: 95°C for 15 min, 22 cycles of amplification (95°C for 30 s, 59°C for 30 s, 72°C for 1 min) and a final extension at 72°C for 5 min. Primer (Invitrogen) sequences can be found in the Table S3. Finally, PCR products were separated by 1.5% agarose gel and stained with GelRed Nucleic Acid Gel Stain (Biotium). GAPDH was used as an internal control.

Cell viability and growth assays

Cell density in 2D cultures of primary cells and HCC1937 was determined by absorbance measurement following fixation and staining with crystal violet. CellTiterGlo cell viability reagent (Promega, Southampton, UK) was used to assess relative cell number of 3D cultured primary cells and MDA-MB-231 cells. The GelCount platform and software (Oxford Optronix, Oxford, UK) were used to automatically determine the size of organoids grown in 3D.

Cell migration and invasion assay

Invasion and migration assays were performed using 24-well Transwell inserts (Corning, Amsterdam, the Netherlands) coated or not with Matrigel, respectively. After 24 hr-starvation cells (75.000) were resuspended in serum-free (250 μL) medium and seeded into the upper chamber. 750 μL of medium supplemented with 10% serum was added to the lower chamber. After 20 hr, cells on the lower side of the insert were fixed, stained with crystal violet and counted under a light microscope.

siRNA Transfection

MDA-MB-468 cells were transfected with Pin1 or control siRNA (Table S3) using Lipofectamine RNAimax reagent (ThermoFisher Scientific) in Opti-MEM serum-free medium (ThermoFisher Scientific). MCF7 cells were transfected with control (Non-Targeting siRNA Pool #1, Dharmacon; see Key Resources Table) or ESRP1 siRNA (ON-TARGETplus, Dharmacon; see Key Resources Table) using DharmaFECT 4 Transfection Reagent (Dharmacon). All analyses were performed 72 hr after transfection.

Lentiviral vectors and cell transduction

pLKO.1 lentiviral vectors carrying shRNA directed to Brca1, c-Kit and Lyn were selected from the corresponding pLKO.1 target gene MISSION TRC shRNA sets (Sigma; see Key Resources Table). The c-Kit knockdown oligos target both c-Kit isoforms.

For *LYNA*, *PIN1, c-KIT* and *Brca2*, *Pin1* knockdown experiments, DNA Oligonucleotide pairs for shRNA specifically targeting *LYNA*, *PIN1*, *Pin1* or shScr were ligated into the into the pENTR^{TM}/U6 Gateway system entry vector (ThermoFisher Scientific). Hairpin sequences were verified and then transferred, together with the U6 promoter, into a Gateway- modified pSEW lentiviral vector (Regan et al., 2012) by LR reaction (ThermoFisher Scientific). ORFs for *Lyn* mutants (*LynA* CA and *Lyn* TK), mouse *LynB,* human *LYNB*, human *LYNA* Y32F, *LYN* variants resistant to shLyn and human *BRCA1* (C61G, L1407P, A1708E) mutants were generated using the Quickchange Lightening site-directed mutagenesis kit (Agilent Technologies, Stockport, Cheshire, UK) according to the manufacturer's instructions. Primers and templates used are listed in Table S3. Successful mutagenesis was verified by sequence analysis. WT or mutagenized ORFs were then inserted into a Gateway modified pWPI lentiviral vector (Regan et al., 2012) by LR reaction. WPI lentiviral vectors carrying HA-wt BRCA1, BRCA1 mutants (C61G, L1407P, A1708E) or ESRP1-FLAG ORFs were obtained following a similar strategy (further details in Table S3; the ESRP1 plasmid was kindly provided by Prof Klaus Holzmann) (Leontieva and Ionov, 2009).

Viral supernatants were generated by co-transfection of the expression vector and two packaging vectors (psPAX2 and pMD2.G) into HEK293T cells. Cells were refed with fresh medium (DMEM/10% FBS; ThermoFisher) after 24 hr. Supernatants were harvested

48 and 72 hr after transfection, checked for absence of replication-competent virus and stored at −80°C until use. Lentiviruses derived from pWPI and pHIV-H2BmRFP plasmids were concentrated by ultracentrifugation (50,000*g*, 2 hr at 4°C). Relative lentiviral titer was determined by transducing NIH 3T3 cells using serial dilutions of the viral preparations. Freshly isolated primary cells were resuspended in viral supernatant (shRNA-carrying vectors) or concentrated viral particles in growth medium (overexpression vectors) and plated on to Matrigel or plastic as required for the specific assay. After 24 hr, medium was replaced with fresh medium (Regan et al., 2012). Puromycin (Sigma) (1.5 μg/ml) was added to culture medium of cells transduced PLKO.1 lentiviral vectors 36 hr after infection.

Generation and expression of LYN-GFP fusion proteins

ORFs for human *LYNA* and *LYNB* were cloned into pEGFP-N3 (EcoRI/BamHI). Primers and templates used are listed in Table S3. MDA-MB-231 cells were transiently transfected with pEGFP-N3-LYN A or pEGFP-N3-LYN B plasmids using Lipofectamine 3000 Reagent (Thermo Fisher Scientific) according to the manufacturer's instructions. After 48 hr cells were fixed, counterstained with DAPI and analyzed by confocal microscopy.

In vitro and *in vivo* Dasatinib treatment

For *in vitro* experiments culture medium with a range of Dasatinib concentrations (Selleckchem, Stratech, Newmarket, Suffolk, UK) was added to cells 24 hr after plating and replaced every other day. Sigmoidal curves from dose-response data were generated using Prism software.

For *in vivo* treatment, Dasatinib monohydrate (Selleckchem) was dissolved in DMSO at 20 mg/mL and stored in aliquots at −20°C. Aliquots were thawed and diluted in 5.1% polyethylene glycol (PEG-400) and 5.1% Tween 80 (vehicle, VEH) before use. Mice were treated with a single intraperitoneal (IP) injection of Dasatinib (DAS) (15 mg/Kg) daily. Control mice were treated with an equivalent concentration of DMSO dissolved in vehicle. Caliper measurements of tumor width (W) and length (L) were recorded every other day and tumor volumes were calculated using the formula $(L \times W^2)/2$).

In vivo conditional Lyn knockdown

Pairs of complementary DNA oligonucleotides (Table S3), encoding shLyn#2 (shLyn) or shScr, were annealed and cloned into a pENTR^{TM}/H1/TO vector (ThermoFisher Scientific). The H1/TO -shLyn or -shScr cassette was then transferred into a Gateway-modified pSEW lentiviral vector (Regan et al., 2012) via LR recombination. ORF of Tetracycline repressor (TetR) was amplified from pcDNA^{TM}6/TR plasmid (ThermoFisher Scientific) (Table S3) and cloned into a pHIV-H2BmRFP lentiviral vector. Primary mouse *BlgCre Brca1$^{fl/fl}$ p53$^{+/-}$* mammary tumor cells (line #2) were transduced using pHIV-RFP-TetR and pSEW-GFP-TO-H1(-shScr or -shLyn) lentiviral vectors. Cells positive for both GFP and RFP expression were then sorted on a FACSAria flow cytometer (BD Biosciences) and assessed for *Lyn* knockdown *in vitro* in the absence or in the presence of doxycyline (0.5 ug/ml). 250,000 (shLyn- or shScr-) cells were orthotopically injected into the fourth right mammary fat pad of nude mice. Mice were randomized to either a control (DOX-) or a doxycicline (DOX+) diet (TD.09761, Harlan Teklad, Harlan, Indianapolis, USA). Tumor volumes were calculated from caliper measurements of tumor width (W) and length (L) using the formula $(L \times W^2)/2$).

Immunofluorescence staining

For immunofluorescence analysis cells were grown in 8-well chamber slides (BD Biosciences) in 3D culture conditions (BD Biosciences). Cells were fixed in 4% formalin for 20 min and washed with PBS-glycine (0.7%) before blocking with PBS/0.1% Bovine Serum Albumin (BSA)/0.2% Triton X-100/0.05% Tween-20/10% goat serum for 1.5 hr.

Cultured MCF10A 3D acini were incubated for 2 hr with antibodies to Ki-67 (clone MM1) diluted 1:50 or to integrin-alpha6 (clone GoH3) diluted 1:100 in blocking buffer prior to incubation with Alexa Fluor® 488 Goat Anti-Mouse or Donkey Anti-Rat secondary antibodies, respectively, for 1 hr. All incubation steps were carried out at room temperature. Counterstaining with DAPI was then followed by mounting using the ProLong Antifade agent (ThermoFisher Scientific).

Indirect TUNEL was performed using The ApopTag® Red *In Situ* Apoptosis Detection Kit (Merck Millipore) following the manufacturer's protocol. Slides were analyzed on a Zeiss LSM 710 confocal microscope using a 20X objective.

Phospho-Histone H3 immunohistochemical staining

Immunohistochemistry was carried out following standard procedures. Fresh sections were cut from formalin-fixed and paraffin-embedded tumor tissue. Dewaxed and re-hydrated slices underwent antigen retrieval in citrate buffer, pH 6.0 (Sigma) in a pressure cooker for 5 min before incubation with a 3% hydrogen peroxyde solution for 20 min and then blocking in 1% BSA/0.1% Tween-20/TBS for 1 hr. Incubation with anti-phospho-Histone H3 (S10) antibodies (rabbit polyclonal, #9701, Cell Signaling Technology; diluted 1:200 in blocking buffer) was performed overnight at 4°C. Detection was carried out using the EnVision+System-HRP kit for rabbit primary antibody (Dako, Ely, Cambridgeshire, UK). Sections were then counterstained with hematoxyilin and mounted. Images were acquired using an Olympus BX43 microscope with a 20x/0.50 Ph1 objective.

PIN1 Immunohistochemistry and analysis of BRCA1 tumor TMA

PIN1 immunohistochemistry (IHC) was carried out by the Northern Ireland Biobank. Briefly, wax was removed from Formalin-Fixed Paraffin-Embedded (FFPE) tissue by three washes with Bond Dewax solution (Leica, Milton Keynes, UK) at 72°C, three washes with alcohol, and three washes with Bond Wash solution (Leica). Proteins were prepared for antibody binding by incubating in Bond Epitope Retrieval 1 solution (Leica) at 100°C for 20 min. Slides were then washed three times with Bond Wash solution. Incubation with primary antibody (anti-PIN1 Sc-46660) at 1:200 dilution was carried out for 15 min. The wash step was repeated before blocking in peroxide for 5min, washing again, and incubating in Post Primary anti-mouse antibody for 8 min. Antibody detection with DAB was carried out using the Bond Polymer Refine Detection kit (Leica) according to the manufacturer's instructions, counterstained in hematoxylin and mounted.

PIN1 scoring was based on a scale of 0-4 where 0 represented no visible staining of PIN1, 1 represents low, 2 represents medium, 3 represents high and 4 represents very high, as per the examples in Figure 4. Each of three cores per patient was scored independently; the highest score of the three was used as the overall score.

Isoform specific expression analysis by Affymetrix

The human LYN A isoform can be detected specifically by the microarray feature 3098998 on the Affymetrix Human Exon 1.0ST arrays. To establish LYN A's levels in human breast cancers, we extracted its isoform-specific expression across 177 previously published breast carcinomas enriched for the triple negative phenotype (Brasó-Maristany et al., 2016; Gazinska et al., 2013) (ArrayExpress accession number E-MTAB-570) and across a panel of breast cancer cell lines (Heiser et al., 2012). For each breast cancer sample, immunohistochemistry-based and PAM50 derived breast cancer subtypes, as well as breast cancer cell line subtypes were retrieved from the original publications, respectively (Gazinska et al., 2013; Heiser et al., 2012).

Isoform specific expression analysis by RNaseq

Level-3 RNaseq data and overall survival was downloaded from TCGA breast cancer (https://cancergenome.nih.gov/). LYNA and LYNB isoforms were manually identified as uc003xsk.* and uc003xsl.* (see Figure S7A for details). Ratios were calculated using raw RSEM values and log transformed for brevity. PAM50 classification was performed as described (Perou et al., 2000). Statistical analyses and respective data plots were generated in R version 3.2.2.

LYN pull-down for Tandem Mass Tag (TMT) labeling

TMT enables robust quantitation and comparison by mass spectrometry of protein levels between samples. MDA-MB-231 (LYN KD, LYN-A*, LYN-B*, LYN-YF*) cells were plated in T175 flasks and after two days were either serum-starved or left untreated overnight. The following day starved cells were treated with 50 ng ul^{-1} EGF for two hr. Next, both treated and untreated cells were lysed in 1% IGEPAL CA-630, 150 mM NaCl, 1mM MgCl2, 50 mM Tris pH 7.5, 5% glycerol, 10 mM Sodium Fluoride, 1 mM Sodium Orthovanadate, 10 mM Sodium Pyrophosphate, 100 mM β-Glycerophosphate and Complete Protease inhibitor cocktail (Roche).

After centrifugation (14000 *g* for 15 min at 4°C), cell lysates (3 mg of protein) were pre-cleared with protein A-Sepharose beads (GE Healthcare) for 45 min at 4°C prior to incubation with anti-LYN antibodies (rabbit polyclonal (44), sc-15, Santa Cruz) overnight at 4°C. After incubation with protein A-Sepharose beads for 45 min at 4°C, immunoprecipitates were pulled down by centrifugation (900 *g* for 5 min at 4°C), washed three times with lysis buffer, twice with lysis buffer devoid of IGEPAL CA-630 and after removal of the supernatants samples were stored at −80°C until being processed for TMT labeling.

TMT Labeling and High pH reversed-phase chromatography

Pull-down samples were digested with trypsin while on the beads (2μg trypsin; 37°C, overnight), labeled with Tandem Mass Tag (TMT) ten plex reagents according to the manufacturer's protocol (Thermo Fisher Scientific, Loughborough, LE11 5RG, UK) and the labeled samples pooled.

The pooled sample was evaporated to dryness, resuspended in 5% formic acid and then desalted using a SepPak cartridge according to the manufacturer's instructions (Waters, Milford, Massachusetts, USA). Eluate from the SepPak cartridge was again evaporated to dryness and resuspended in buffer A (20 mM ammonium hydroxide, pH 10) prior to fractionation by high pH reversed-phase chromatography using an Ultimate 3000 liquid chromatography system (Thermo Scientific). The sample was loaded onto an XBridge BEH C18 Column (130Å, 3.5 μm, 2.1 mm X 150 mm, Waters, UK) in buffer A and peptides eluted with an increasing gradient of buffer B (20 mM Ammonium Hydroxide in acetonitrile, pH 10) from 0%–95% over 60 min. The resulting fractions were evaporated to dryness and resuspended in 1% formic acid prior to analysis by nano-LC MSMS using an Orbitrap Fusion Tribrid mass spectrometer (Thermo Scientific).

Nano-LC Mass Spectrometry

High pH RP fractions were further fractionated using an Ultimate 3000 nano-LC system in line with an Orbitrap Fusion Tribrid mass spectrometer (Thermo Scientific). Peptides in 1% (vol/vol) formic acid were injected onto an Acclaim PepMap C18 nano-trap column (Thermo Scientific). After washing with 0.5% (vol/vol) acetonitrile 0.1% (vol/vol) formic acid peptides were resolved on a 250 mm × 75 μm Acclaim PepMap C18 reverse phase analytical column (Thermo Scientific) over a 150 min organic gradient, using 7 gradient segments (1%–6% solvent B over 1min., 6%–15% B over 58min., 15%–32%B over 58min., 32%–40%B over 5min., 40%–90%B

over 1min., held at 90%B for 6min and then reduced to 1%B over 1min.) with a flow rate of 300 nL min^{-1}. Solvent A was 0.1% formic acid and Solvent B was aqueous 80% acetonitrile in 0.1% formic acid. Peptides were ionized by nano-electrospray ionization at 2.0kV using a stainless steel emitter with an internal diameter of 30 μm (Thermo Scientific) and a capillary temperature of 275°C.

All spectra were acquired using an Orbitrap Fusion Tribrid mass spectrometer controlled by Xcalibur 2.0 software (Thermo Scientific) and operated in data-dependent acquisition mode using an SPS-MS3 workflow. FTMS1 spectra were collected at a resolution of 120 000, with an automatic gain control (AGC) target of 200 000 and a max injection time of 50ms. Precursors were filtered with an intensity threshold of 5000, according to charge state (to include charge states 2-7) and with monoisotopic precursor selection. Previously interrogated precursors were excluded using a dynamic window (60 s ± 10ppm). The MS2 precursors were isolated with a quadrupole mass filter set to a width of 1.2 m/z. ITMS2 spectra were collected with an AGC target of 10 000, max injection time of 70ms and CID collision energy of 35%.

For FTMS3 analysis, the Orbitrap was operated at 50 000 resolution with an AGC target of 50 000 and a max injection time of 105ms. Precursors were fragmented by high energy collision dissociation (HCD) at a normalized collision energy of 60% to ensure maximal TMT reporter ion yield. Synchronous Precursor Selection (SPS) was enabled to include up to 5 MS2 fragment ions in the FTMS3 scan.

TMT Data Analysis

The raw data files were processed and quantified using Proteome Discoverer software v2.1 (Thermo Scientific) and searched against the UniProt Human database (downloaded 14/09/17; 140000 sequences) plus LYNA and LYNB and LYNA_YF sequences using the SEQUEST algorithm. Peptide precursor mass tolerance was set at 10ppm, and MS/MS tolerance was set at 0.6Da. Search criteria included oxidation of methionine (+15.9949) as a variable modification and carbamidomethylation of cysteine (+57.0214) and the addition of the TMT mass tag (+229.163) to peptide N-termini and lysine as fixed modifications. Searches were performed with full tryptic digestion and a maximum of 2 missed cleavages were allowed. The reverse database search option was enabled and the data were filtered to satisfy false discovery rate (FDR) of 5%.

QUANTIFICATION AND STATISTICAL ANALYSIS

Unless otherwise stated, blots shown are representative of three independent experiments. Unless otherwise stated, all quantitation is shown as mean and SD from three independent experiments and statistical significance determined using two-tailed unpaired t tests. Gene expression analysis by quantitative real-time rtPCR is shown as mean ± 95% confidence intervals from three independent experiments, each of which was carried out using three technical replicates. Significance of real-time RT-PCR data was determined from confidence intervals (Cumming et al., 2007). *$p < 0.05$; **$p < 0.01$; ***$p < 0.001$.

Statistical analysis of tumor growth was conducted using the *glmer* function for generalized linear mixed models from the *lme4* package (Bates et al., 2015) in the R software (version 3.2.2). The final model accounted for the change in tumor VOLUME with time (DAY) and a DAY-by-TREATMENT interaction as fixed effects using variable random intercepts and slopes for each tumor (TUMOUR_ID). This relationship was specified as glmer (VOLUME ~ DAY + DAY:TREATMENT + (DAY|fTUMOUR_ID), family = Gaussian (link = “log). All modelling assumptions were confirmed to be reasonable on diagnostic residual plots.

Number of phospho-H3-positive cells in FFPE sections of grafted tumors was determined by using ImageJ image analysis software (https://imagej.nih.gov/ij/). Automatic counting was performed on binary images (8-12 fields per tumor) after applying consecutive dilations to coalesce multiple dots within the same cell.

Band intensities on gels and western blots were also quantified using ImageJ.

Cell Reports, Volume *25*

Supplemental Information

Dual Mechanisms of LYN Kinase Dysregulation

Drive Aggressive Behavior in Breast Cancer Cells

Giusy Tornillo, Catherine Knowlson, Howard Kendrick, Joe Cooke, Hasan Mirza, Iskander Aurrekoetxea-Rodríguez, Maria d.M. Vivanco, Niamh E. Buckley, Anita Grigoriadis, and Matthew J. Smalley

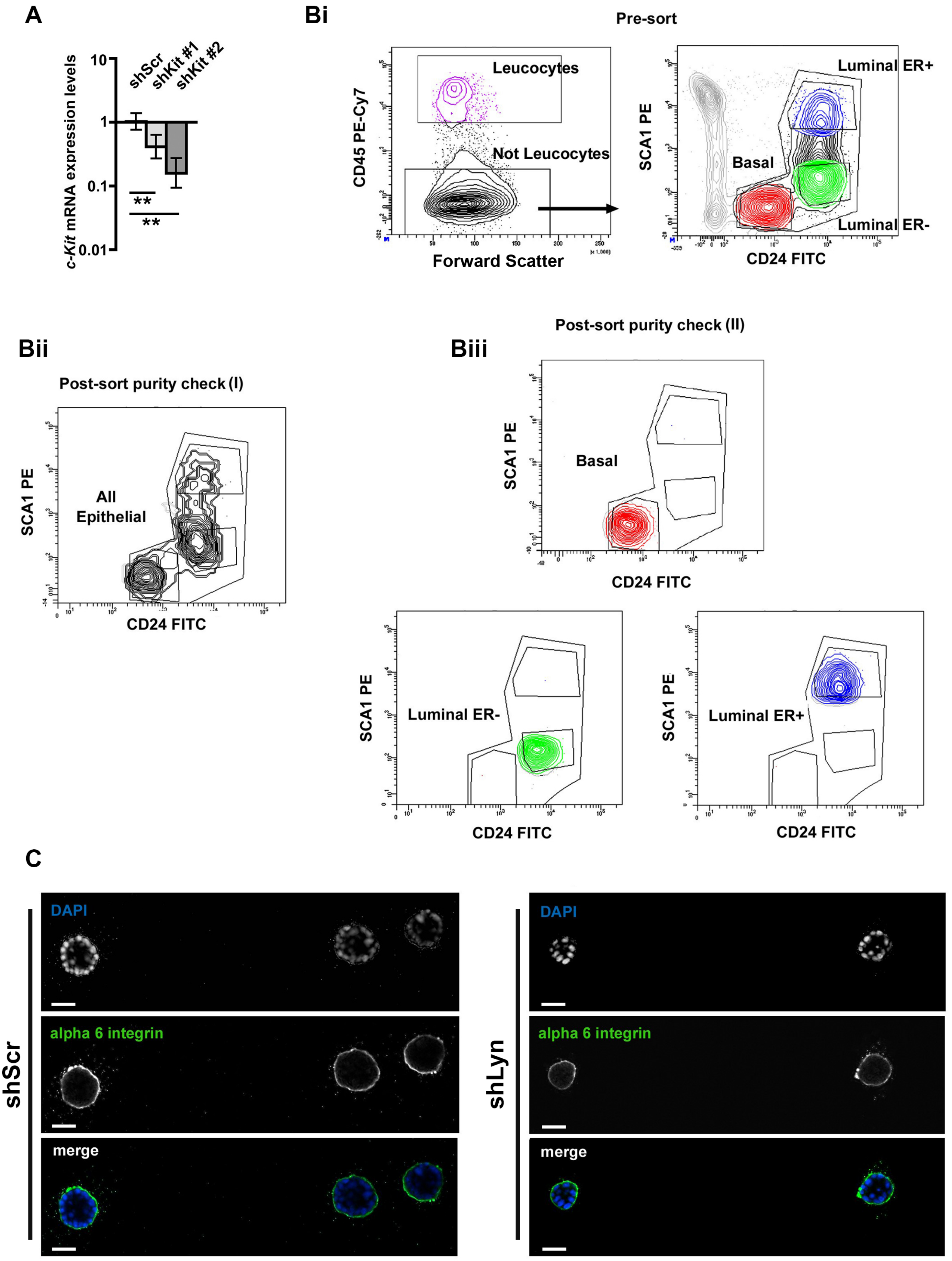
A
c-Kit mRNA expression levels
10
1
0.1
0.01
shScr
shKit #1
shKit #2
**
**
Bi
Pre-sort
CD45 PE-Cy7
Leucocytes
Not Leucocytes
Forward Scatter
SCA1 PE
Luminal ER+
Basal
Luminal ER-
CD24 FITC
Bii
Post-sort purity check (I)
SCA1 PE
All
Epithelial
CD24 FITC
Biii
Post-sort purity check (II)
SCA1 PE
Basal
CD24 FITC
SCA1 PE
Luminal ER-
CD24 FITC
SCA1 PE
Luminal ER+
CD24 FITC
C
shScr
DAPI
alpha 6 integrin
merge
shLyn
DAPI
alpha 6 integrin
merge

Figure S1. ***c-Kit*** **expression following shKit transduction, isolation of Luminal ER- progenitor cells and assessment of polarity in MCF10A acini (related to Figure 1 and Figure 2). (A)** Analysis of *c-Kit* expression by qrtPCR in primary mouse mammary organoids after transduction with control (shScr) or c-Kit-knock-down (shKit#1 and shKit#2) lentiviruses (mean±95% confidence intervals; significance of real time rtPCR data determined from confidence intervals; n=3 independent experiments each of 3 technical replicates per sample) (Cumming et al., 2007). **P<0.01 **(B) (i)** Flow cytometry of primary mammary cells stained with antibodies against CD45, CD24 and Sca-1. $CD45^+$ leucocytes (purple) were gated out (left plot) and the $CD45^-$ cells (right plot) gated to fractionate All Epithelial cells or Basal ($CD24^{+/low}$ $Sca\text{-}1^-$, red), Luminal ER- ($CD24^{+/high}$ $Sca\text{-}1^-$, green), and Luminal ER+ ($CD24^{+/high}$ $Sca\text{-}1^+$, blue) epithelial cell subpopulations as we have previously described (Kendrick et al., 2008; Regan et al., 2012; Sleeman et al., 2007). Post-sort purity checks for total epithelial cells **(ii)** and sorted sub-populations **(iii)** also shown. **(C)** Polarity of shScr-, shLyn#1- or shLyn#2-transduced MCF10A cells in 3D culture (day 12) determined by immunofluorescence analysis of alpha6 integrin expression. Scale bar, 50 μm.

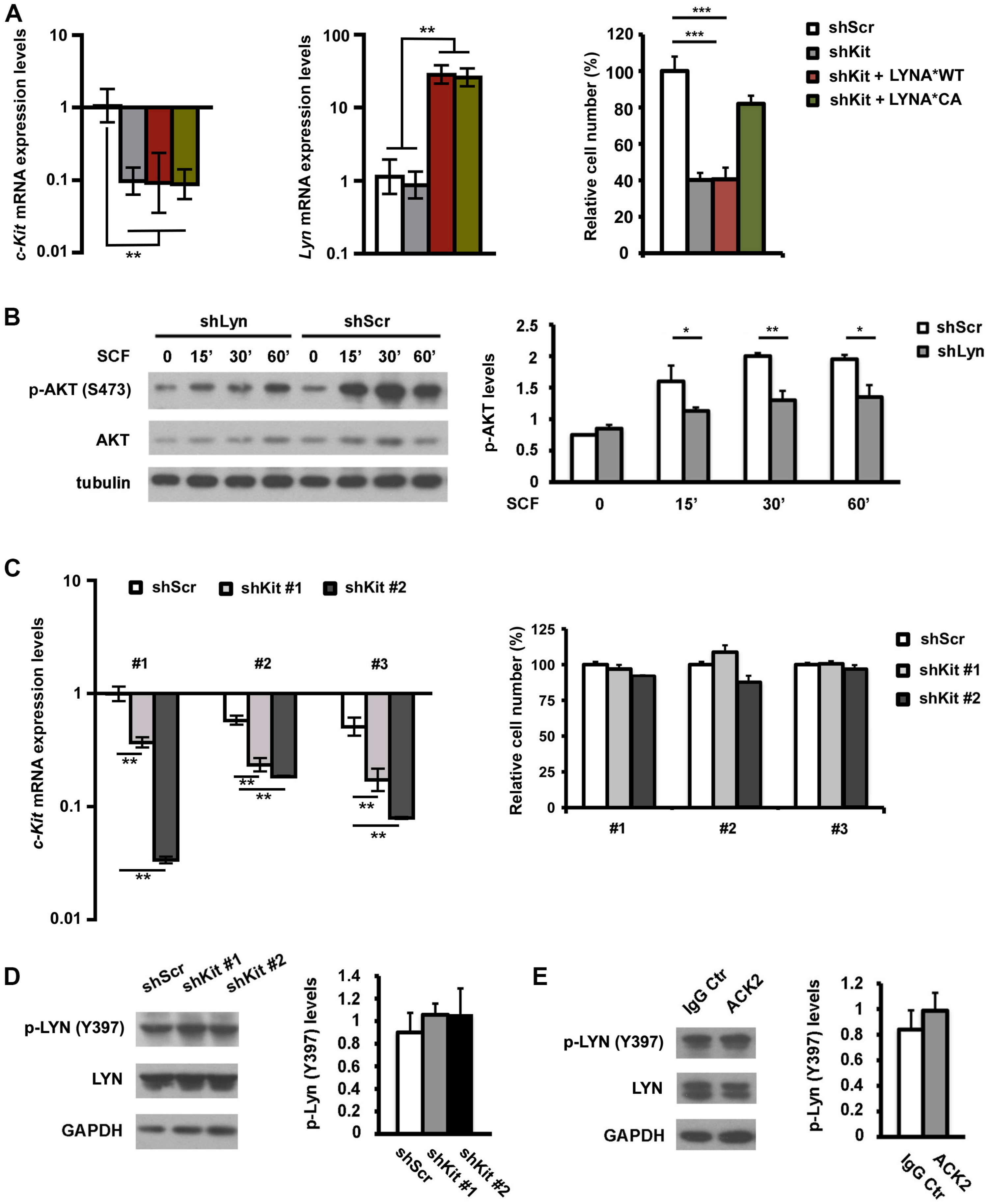

A
c-Kit mRNA expression levels
10
1
0.1
0.01
**
Lyn mRNA expression levels
100
10
1
0.1
**
Relative cell number (%)
120
100
80
60
40
20
0
***
***
shScr
shKit
shKit + LYNA*WT
shKit + LYNA*CA
B
shLyn
shScr
SCF
0
15'
30'
60'
p-AKT (S473)
AKT
tubulin
p-AKT levels
2.5
2
1.5
1
0.5
0
*
**
*
shScr
shLyn
C
c-Kit mRNA expression levels
shScr
shKit #1
shKit #2
#1
#2
#3
**
Relative cell number (%)
125
100
75
50
25
0
D
shScr
shKit #1
shKit #2
p-LYN (Y397)
LYN
GAPDH
p-Lyn (Y397) levels
1.4
1.2
1
0.8
0.6
0.4
0.2
0
E
IgG Ctr
ACK2

Figure S2. c-KIT promotes proliferation and survival of normal mammary epithelial cells but not BRCA1 tumor cells (related to Figure 2). **(A)** Mammary organoids were transduced with shScr and empty expression vectors, shKit and empty expression-vectors, or shKit and expression vectors carrying either wild-type LYNA (LYNA WT) or a constitutively active LYNA mutant (LYNA CA). *c-Kit* and *Lyn* expression levels were determined by qrtPCR 5 days after transduction (left and middle panels). Bar graph (right) shows cell number at day 5 of culture relative to shScr cells. **(B)** Western blot analysis (left) of AKT phosphorylation (S473) and total AKT levels in protein extracts from primary mouse mammary organoids transduced with either control (shScr) or *Lyn* knockdown (shLyn) lentiviruses, either unstimulated (0') or stimulated with SCF for 15, 30 or 60 minutes. Tubulin used as loading control. Right, quantification of phospho-AKT levels (normalized to total AKT). **(C)** *Brca1*-tumor derived cell lines (#1, #2, #3) were transduced with shScr control or two c-KIT knockdown lentivirus (shKit#1 and t#2). Left panel, qrtPCR analysis of *c-Kit* expression relative to shScr cells. Right panel, relative cell number of shScr, shKit#1 and #2 cells. **(D, E)** LYN autophosphorylation (Y397) in protein extracts from *Brca1*-tumor derived cells after transduction with control (shScr) or c-Kit-knockdown (shKit#1 and #2) lentivirus **(D)** or treatment with c-KIT blocking (ACK2) or IgG isotype (IgG Ctr) antibodies **(E)**. Blots are representative of three independent experiments. Quantitation shown as mean and SD (n=3 biological replicates; two-tailed unpaired t-tests), except for gene expression analysis by quantitative real time rtPCR (mean±95% confidence intervals; significance of real time rtPCR data determined from confidence intervals; n=3 independent experiments each of 3 technical replicates per sample) (Cumming et al., 2007). *P<0.05; **P<0.01; ***P<0.001.

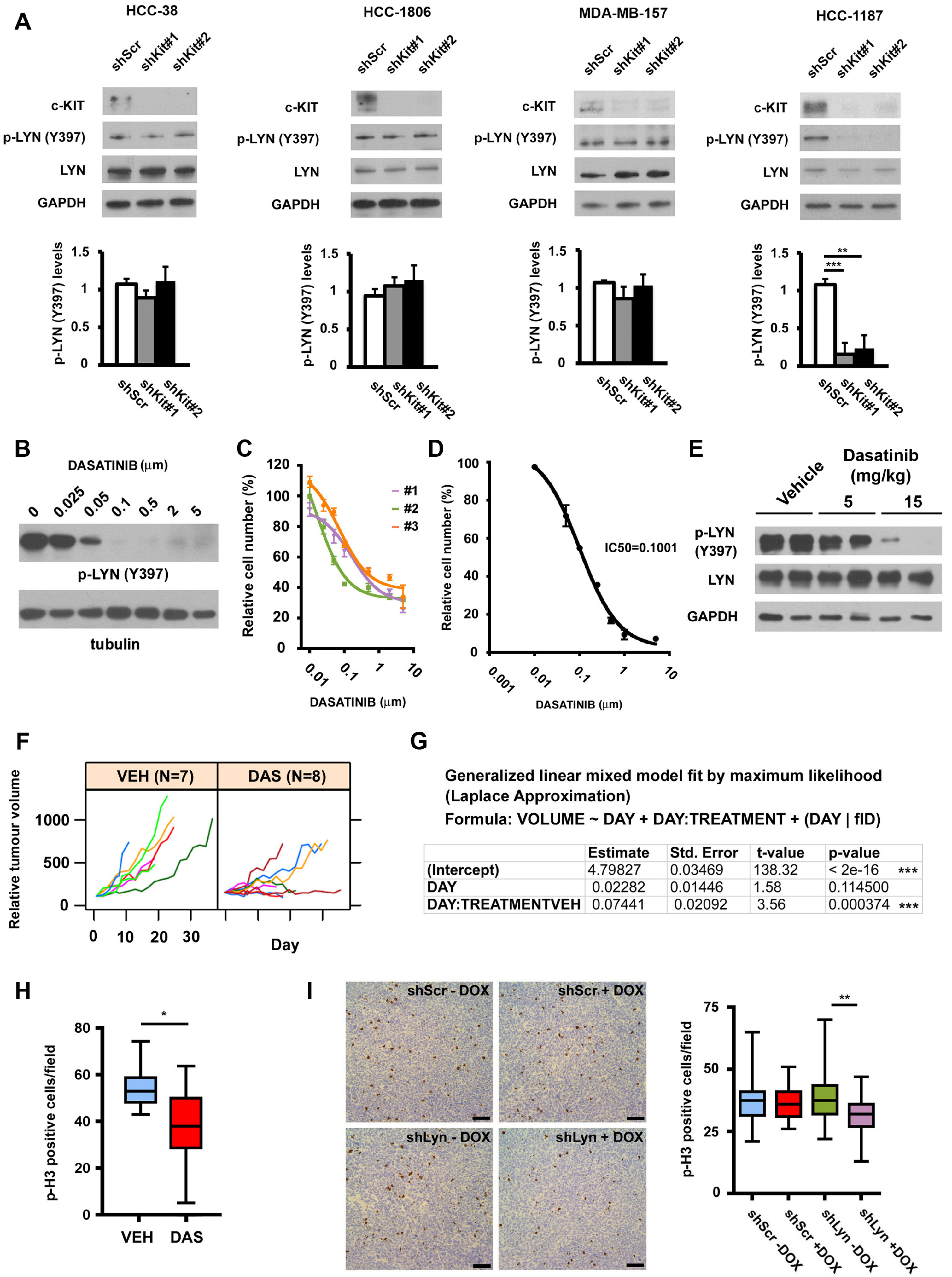

	Estimate	Std. Error	t-value	p-value	
(Intercept)	4.79827	0.03469	138.32	< 2e-16	***
DAY	0.02282	0.01446	1.58	0.114500	
DAY:TREATMENTVEH	0.07441	0.02092	3.56	0.000374	***

Figure S3. LYN phosphorylation is not affected by c-KIT knockdown in BRCA1 defective human breast cancer cell lines and Dasatinib both inhibits the growth of BRCA1 defective cell lines and blocks BRCA1 tumour growth *in vivo* (related to Figure 2 and Figure 3). (A) Representative western blot analyses and quantitation from three independent experiments of LYN autophosphorylation (Y397) following knockdown of c-KIT with two independent shRNAs in four c-KIT+ human breast cancer cell lines, HCC-38 (*BRCA1* silenced by methylation), HCC-1806 and MDA-MB-157 (BRCA1 low due to miRNA expression) and HCC-1187 (BRCA1 wt). **(B)** Western blot analysis of LYN autophosphorylation (Y397) in mammary organoids following treatment with vehicle or Dasatinib at concentrations from 0.025 to 5 μM. Tubulin used as loading control. **(C)** Primary *BlgCre* $Brca1^{fl/fl}$ $p53^{+/-}$ mouse mammary tumour cells (#1, #2 and #3) were seeded on Matrigel and treated with vehicle or the indicated Dasatinib concentrations for 5 days. Cell number assessed by CellTitreGlo assay relative to vehicle-only treated cells. Graph show dose response curves using a Nonlinear Regression (Curve Fit) model. **(D)** HCC1937 cells were treated with vehicle or the indicated Dasatinib concentrations stained with crystal violet after 7 days. Viable cell density was determined as in Figure 4A. Graph shows dose response curve and IC50 derived from nonlinear curve fitting (right panel). Blots are representative of three independent experiments. Unless otherwise stated, quantitation shown as mean and SD from three independent experiments and statistical significance determined using two-tailed unpaired t-tests. *P<0.05; **P<0.01; ***P<0.001. **(E)** Wild-type mice were treated with daily intra-peritoneal injection (IP) of vehicle (N=2), Dasatinib - 5mg/Kg (N=2) or Dasatinib - 15mg/Kg (N=2) for 7 days. Mammary glands were harvested from each mouse 6 hours after last injection and lysed. Protein extracts were analyzed for levels of phosphorylated (Y397) and total LYN by western blot. GAPDH was used as loading control. **(F)** *Blg-Cre* $Brca1^{fl/fl}p53^{+/-}$ mice carrying mammary tumours were treated with daily IP injection of vehicle (VEH, N=7) or Dasatinib (DAS, 15mg/Kg, N=8). Tumour size was recorded every two days. Tumour volume was calculated and plotted as relative to volume at day 0 of treatment. **(G)** Statistical analysis of tumour growth in vehicle- and Dasatinib-treated *Blg-Cre* $Brca1^{fl/fl}p53^{+/-}$ mice using the *glmer* function for generalized linear mixed models from the *lme4* package in the R software. The summary of the final model (VOLUME ∼ DAY + DAY:TREATMENT + (DAY|fTUMOUR_ID), family = gaussian (link = "log) is shown in the table. **(H)** Quantitation of phospho-histone3 (pH3) immunohistochemical staining of tumours derived from vehicle- or Dasatinib-treated *BlgCre* $Brca1^{fl/fl}$ $p53^{+/-}$ mice. Ten fields from each tumour were counted. **(I)** Phospho-Histone3 (pH3) immunohistochemical staining of tumours derived from transduced and transplanted *BlgCre* $Brca1^{fl/fl}$ $p53^{+/-}$ tumour cells (arrowheads indicate mitotic pH3-positive cells in insets) Scale bar = 100 um. Representative images and quantification of percentage of pH3-positive cells (box-plots) from ten fields each tumour (seven tumours per group). Blots are representative of three independent experiments. Quantitation shown as mean and SD from three independent experiments. Statistical significance was determined using two-tailed unpaired t-tests unless otherwise stated. *P<0.05; **P<0.01; ***P<0.001.

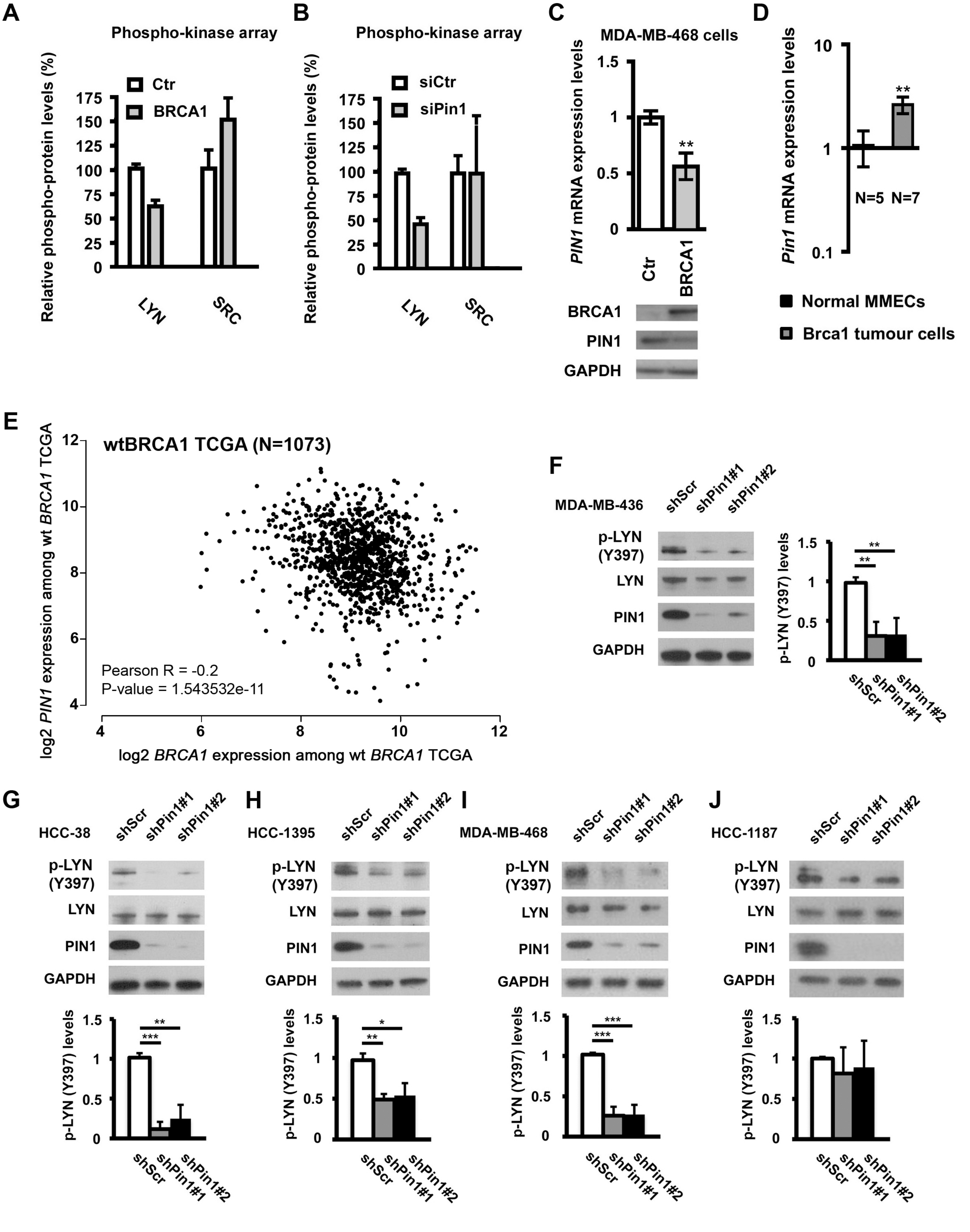
A
Phospho-kinase array
Ctr
BRCA1
Relative phospho-protein levels (%)
LYN
SRC
B
Phospho-kinase array
siCtr
siPin1
Relative phospho-protein levels (%)
LYN
SRC
C
MDA-MB-468 cells
PIN1 mRNA expression levels
Ctr
BRCA1
BRCA1
PIN1
GAPDH
D
Pin1 mRNA expression levels
N=5 N=7
Normal MMECs
Brca1 tumour cells
E
wtBRCA1 TCGA (N=1073)
Pearson R = -0.2
P-value = 1.543532e-11
log2 PIN1 expression among wt BRCA1 TCGA
log2 BRCA1 expression among wt BRCA1 TCGA
F
MDA-MB-436
shScr
shPin1#1
shPin1#2
p-LYN (Y397)
LYN
PIN1
GAPDH
p-LYN (Y397) levels
G
HCC-38
H
HCC-1395
I
MDA-MB-468
J
HCC-1187

Figure S4: LYN activity is regulated by BRCA1 via the prolyl isomerase PIN1 (related to Figure 4). (A, B) Quantification of relative phosphorylation levels of LYN and SRC proteins in MDA-MB-468 cells stably transfected with empty vector (Ctr) or wild-type BRCA1 (BRCA1) **(A)** and MDA-MB-468 cells transiently transfected with scrambled (siCtr) or PIN1 siRNA (siPin1) **(B).** Phosphorylation levels were determined by using a phospho-kinase array (R&D) and densitometry-based quantification by ImageJ (mean±SEM). **(C)** Human MDA-MB-468 BRCA1-low cells were stably transfected with either an empty vector (Ctr) or a vector carrying wild-type BRCA1 (BRCA1). PIN1 expression levels were assessed by qrtPCR (top) and western blot (below). Blots were also probed for BRCA1 and GAPDH as a loading control. **(D)** *Pin1* mRNA expression levels in primary mouse mammary epithelial cells from wild-type mice (normal MMECs, N= 5 preparations) and primary *BlgCre Brca1*$^{fl/fl}$ *p53*$^{+/-}$ mouse tumour cells (N=7 tumours). **(E)** Correlation between BRCA1 and PIN1 expression levels in germline wildtype BRCA1 breast cancers. Log2 base expression levels for PIN1 and BRCA1 were downloaded from TCGA level 3, for 1,073 TCGA BRCA1 wild type breast cancers. Pearson correlation was performed between the two gene expression levels across all samples. P value and r value are shown above the plot. **(F – J)** Representative western blot analyses and quantitation from three independent experiments of LYN autophosphorylation (Y397) following knockdown of PIN1 with two independent shRNAs in five cell lines, (F) MDA-MB-436 (BRCA1 mutant), (G) HCC-38 (BRCA1 deficient), (H) HCC-1395 (BRCA1 mutant), (I) MDA-MB-468 (BRCA1 low) and (J) HCC-1187 (BRCA1 wild type) (mean+SD; unpaired two-tailed t-test). *P<0.05; **P<0.01; ***P<0.001.

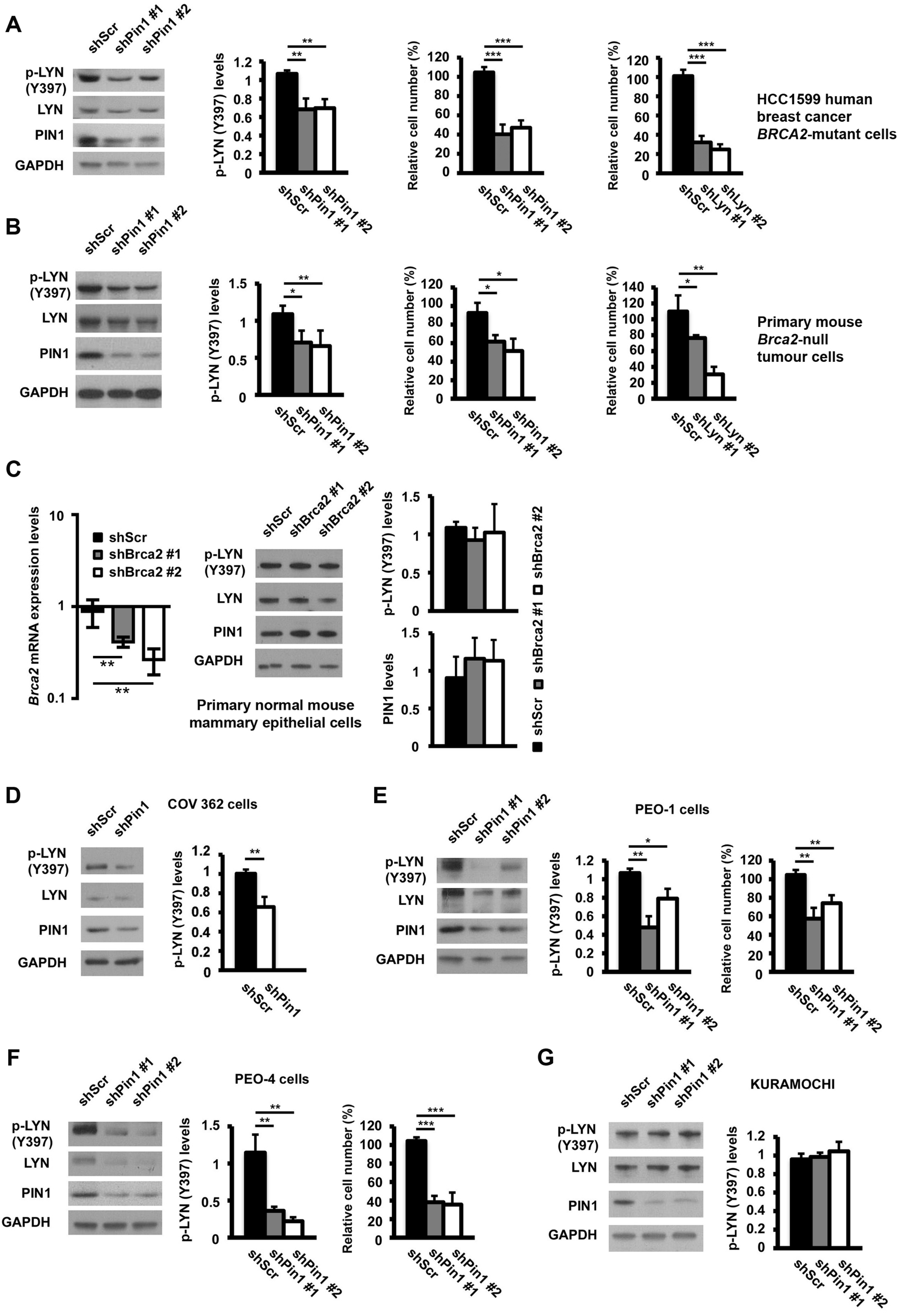

A
shScr
shPin1 #1
shPin1 #2
p-LYN (Y397)
LYN
PIN1
GAPDH
p-LYN (Y397) levels
Relative cell number (%)
shLyn #1
shLyn #2
HCC1599 human breast cancer BRCA2-mutant cells
B
Primary mouse Brca2-null tumour cells
C
Brca2 mRNA expression levels
shBrca2 #1
shBrca2 #2
Primary normal mouse mammary epithelial cells
PIN1 levels
D
COV 362 cells
shPin1
E
PEO-1 cells
F
PEO-4 cells
G
KURAMOCHI

Figure S5. PIN1 regulates LYN phosphorylation in BRCA2 null breast and ovarian cells, but BRCA2 does not regulate PIN1 (related to Figure 5).(A) Western blot analysis of LYN autophosphorylation levels in human HCC1599 BRCA2-deficient breast cancer cells transduced with either control (shScr) or or PIN1-knock-down lentiviruses (shPin1 #1 and shPin1 #2)(left). ShScr, shPin1 #1 and shPin1 #2 cells were also assayed for cell growth (middle). HCC1599 cells were also transduced with control (shScr) or Lyn-knock-down lentiviruses (shLyn#1 and shLyn#2) and tested for cell viability (right). **(B)** Primary cells isolated from three distinct *BlgCre* $Brca2^{fl/fl}$ $p53^{fl/fl}$ mouse mammary tumours were transduced with control (shScr) or *Pin1*-knock-down lentiviruses (shPin1 #1 and shPin1 #2) and assessed for p-LYN (Y397) levels by Western blot (left). ShScr, shPin1 #1 and shPin1 #2 *BlgCre* $Brca2^{fl/fl}$ $p53^{fl/fl}$ cells were seeded at low density in adherent conditions (2D) and stained with crystal violet after 6 days. Viable cell density was determined by absorbance measurement following solubilization of the dye (middle). *BlgCre* $Brca2^{fl/fl}$ $p53^{fl/fl}$ cells were also transduced with control (shScr) or Lyn-knock-down lentiviruses (shLyn#1 and shLyn#2) and tested for cell viability (right). **(C)** Primary mouse mammary organoids were transduced with control (shScr) or *Brca2* knock-down lentiviruses (shBrca2 #1 and shBrca2 #2). *Brca2* knock-down was assessed by qrtPCR (left; fold expression over comparator shScr cells). shScr and shBrca2 cells were assessed for levels of phospho-LYN (Y397), LYN, PIN1 and GAPDH by western blot after 4 days (middle panel, representative blot; right panel quantitation). **(D)** Western blot analysis of LYN autophosphorylation levels in human COV 362 BRCA1-deficient ovarian cancer cells transduced with either control (shScr) or PIN1-knock-down lentivirus (shPin1). **(E)** Analysis of LYN levels and cell viability in PEO1 human ovarian cancer cells after transduction with control (shScr) or PIN1-knock-down lentiviruses (shPin1 #1 and shPin1 #2). **(F)** Analysis of LYN levels and cell viability in PEO4 human ovarian cancer cells after transduction with control (shScr) or PIN1-knock-down lentiviruses (shPin1 #1 and shPin1 #2). **(G)** p-LYN (Y397) levels were measured in shScr, shPin1 #1 and shPin1 #2-transduced cells in KURAMOCHI human ovarian cancer cells. Blots are representative of three independent experiments. Quantitation shown as mean and SD from three independent experiments. Statistical significance was determined using two-tailed unpaired t-tests, except for gene expression analysis by quantitative real time rtPCR which is shown as mean±95% confidence intervals; significance of real time rtPCR data was determined from confidence intervals (n=3 independent experiments each of 3 technical replicates per sample) (Cumming et al., 2007). *P<0.05; **P<0.01; ***P<0.001.

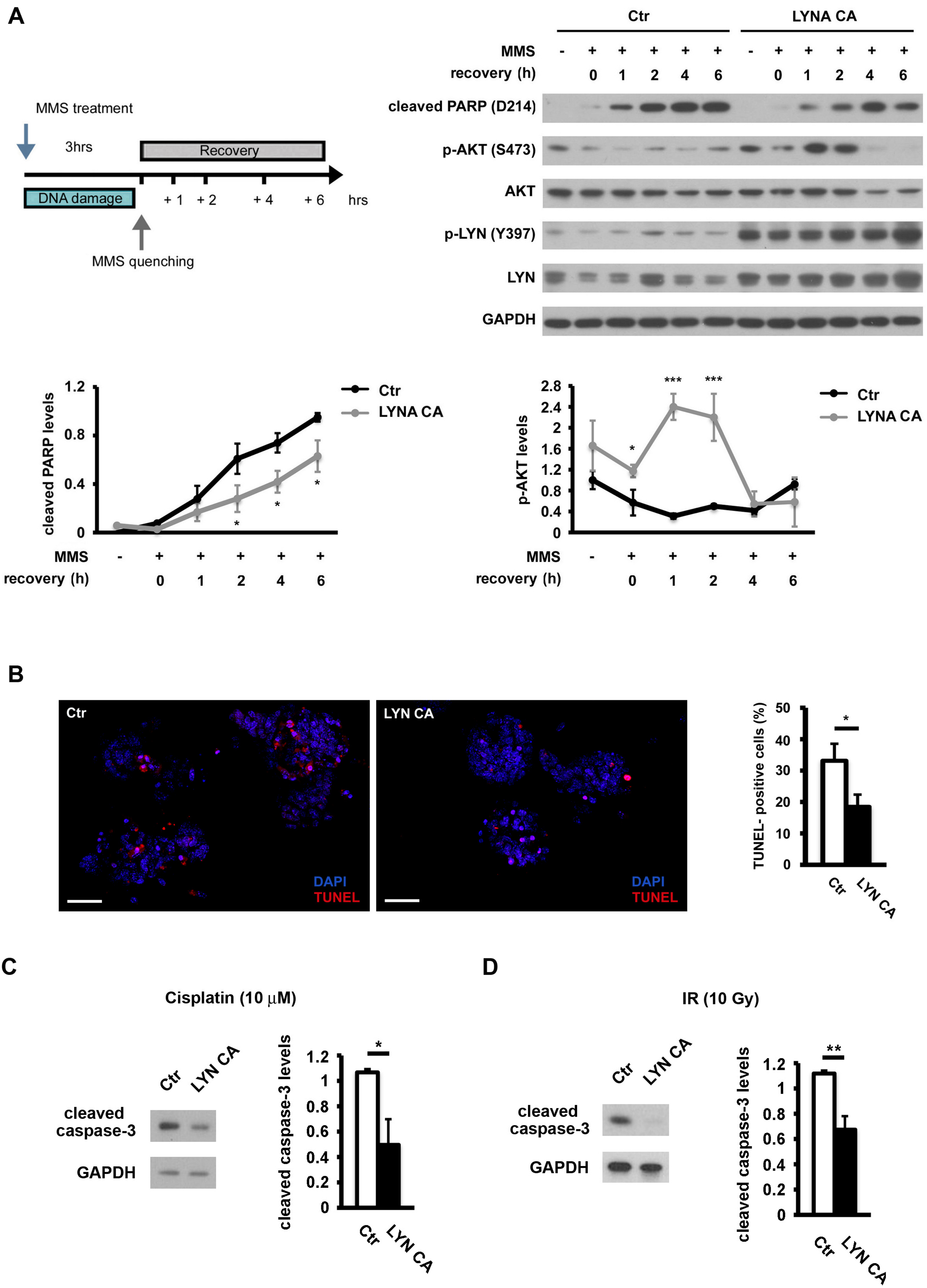

A
MMS treatment
3hrs
Recovery
DNA damage
+ 1 + 2 + 4 + 6 hrs
MMS quenching
Ctr
LYNA CA
MMS
recovery (h)
cleaved PARP (D214)
p-AKT (S473)
AKT
p-LYN (Y397)
LYN
GAPDH
cleaved PARP levels
p-AKT levels
B
Ctr
LYN CA
DAPI
TUNEL
TUNEL- positive cells (%)
C
Cisplatin (10 μM)
cleaved caspase-3
cleaved caspase-3 levels
D
IR (10 Gy)

Figure S6. LYN protects normal mammary from apoptosis in response to DNA damage (related to Figure 5). (A) Response of primary mouse mammary organoids transduced with either an empty control (Ctr) lentiviral vector or a vector expressing a constitutively active mutant LYN (LYN CA) to Methyl Methane Sulfonate (MMS). 4 days after transduction both Ctr- and LYN CA-organoids were exposed to 100 uM MMS or left untreated. After 3 hours, cells were lysed or, following MMS quenching with sodium thiosulfate (2.5%), left to recover for 1, 2, 4 or 6 hours before lysis. Top left, schematics of the experimental conditions. Lysates were analyzed for phospho-LYN (Y397), LYN, cleaved PARP, phospho-AKT (S473), AKT and GAPDH by western blot (top right). Quantification of phospho-AKT and cleaved PARP levels, relative to AKT and GAPDH levels, respectively, are shown below. **(B)** Detection of i*n situ* apoptosis by TUNEL immunofluorescent staining in MMS-treated Ctr- or LYN CA-transduced organoids. 4 days after transduction organoids were treated with 1 uM MMS. After 2 hours, MMS was quenched and cells were left to recover for 24 hours before fixation. Left, representative images (bar=50 um). Right, quantitation. **(C, D)** Analysis and quantitation of cleaved caspase 3 levels in primary organoids transduced as in (B) and then either treated with Cisplatin (10 uM) for 48 hours before lysis (C) or exposed to ionising radiation (IR, 10 Gy) and left to recover for 24 hours before lysis (D). Blots are representative of three independent experiments. Unless otherwise stated, quantitation shown as mean and SEM from three independent experiments. Statistical significance was determined using two-tailed unpaired t-tests. *$P<0.05$; **$P<0.01$; ***$P<0.001$.

A LYN exons and isoforms (UCSC)

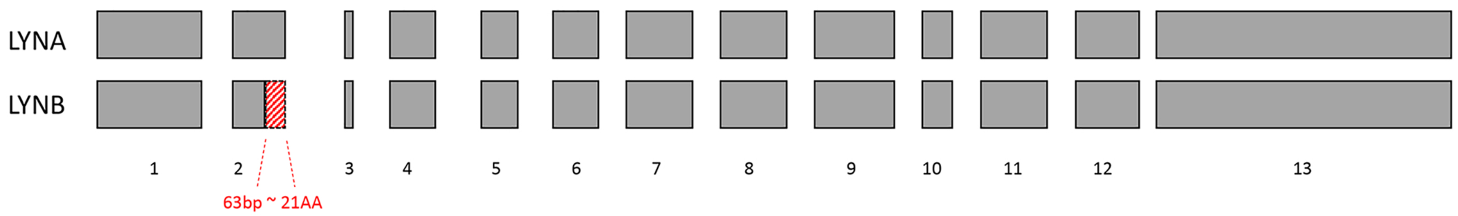

ExonNumber	exonStarts	exonEnds(uc003xsk.*, LYNA)	exonEnds(uc003xsl.*, LYNB)	ExonLength(LYNA)	ExonLength(LYNB)	DifferenceNT	DifferenceAA
Exon1	56792385	56792662	56792662	277	277	0	0
Exon2	56854413	56854550	56854487	137	74	63	21
Exon3	56859006	56859052	56859052	46	46	0	0
Exon4	56860176	56860282	56860282	106	106	0	0
Exon5	56863017	56863116	56863116	99	99	0	0
Exon6	56863239	56863343	56863343	104	104	0	0
Exon7	56864524	56864674	56864674	150	150	0	0
Exon8	56866390	56866543	56866543	153	153	0	0
Exon9	56879273	56879456	56879456	183	183	0	0
Exon10	56882275	56882352	56882352	77	77	0	0
Exon11	56910904	56911058	56911058	154	154	0	0
Exon12	56911976	56912108	56912108	132	132	0	0
Exon13	56922466	56925006	56925006	2540	2540	0	0

B

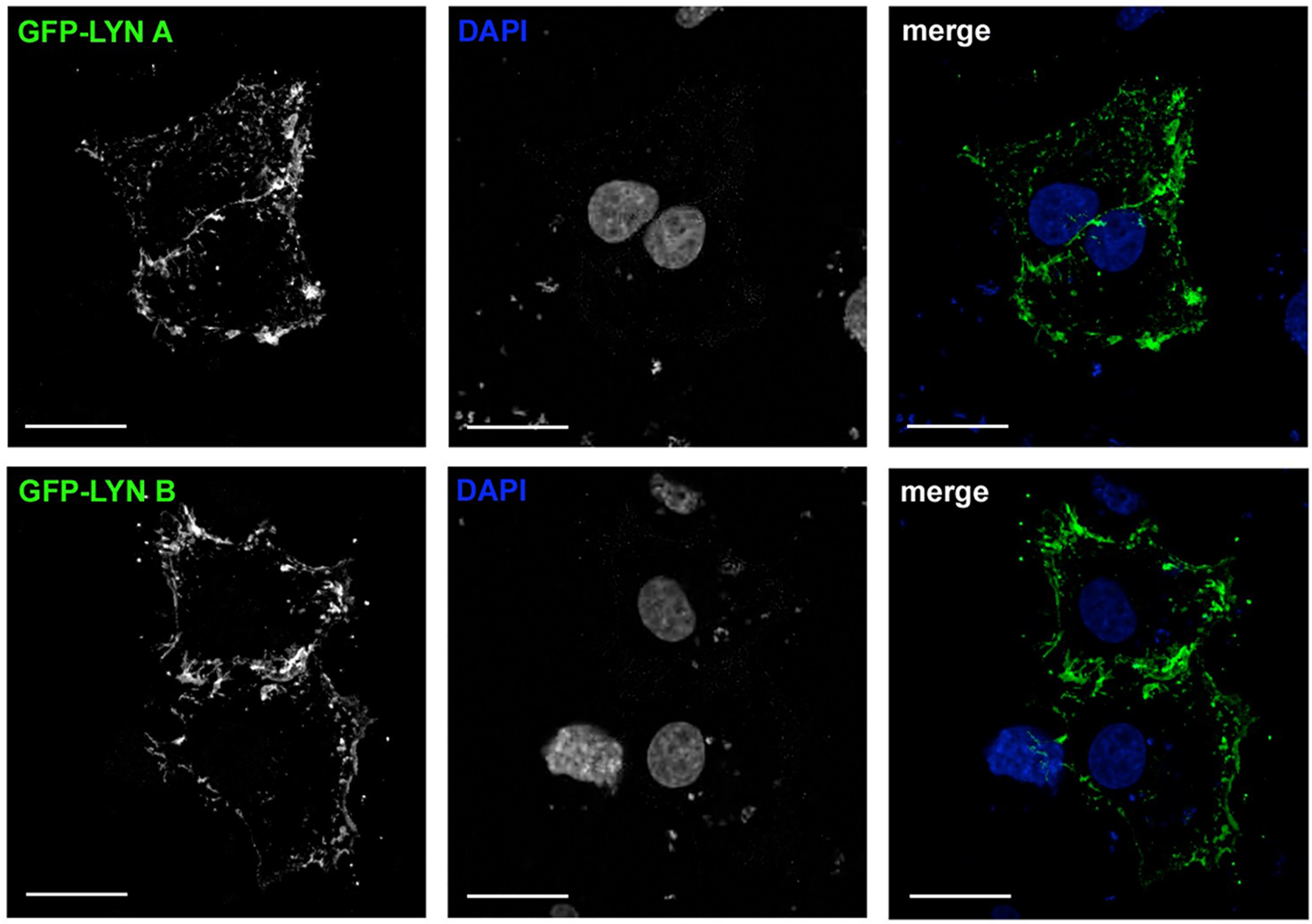

C

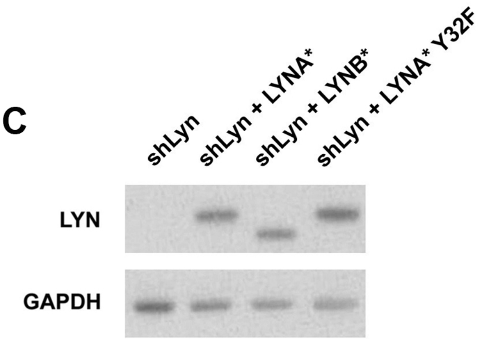

D

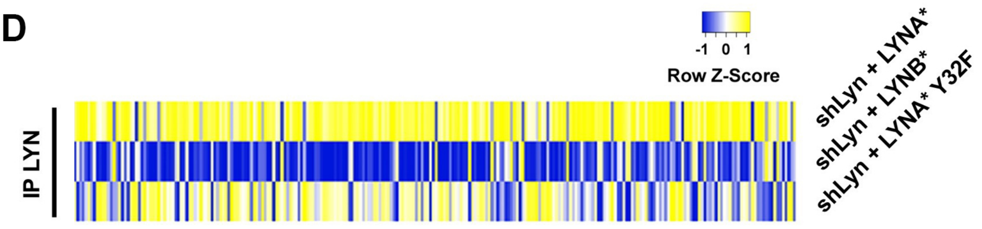

Figure S7. LYN isoforms have similar localisation but different binding partners (related to Figure 6). **(A)** Details of LYN isoforms and exon structure. **(B)** Localisation of LYNA-GFP and LYNB-GFP fusion proteins in transiently transfected MDA-MB-231 cells. Scale bar, 20 μm. **(C)** Representative western blot showing total LYN knock-down (shLyn) and LYN-A*, LYN-B* or LYN-A*^{Y32F} mutant reconstitution using shLyn-resistant forms (LYN-A*, B* or A*^{YF32}) in MDA-MB-231 cells. **(D)** Heatmap visualization of relative abundance levels of LYN co-immunoprecipitated proteins from shLyn + LYN-A*, LYN-B* or LYN-A*^{YF32} MDA-MB-231 cells. Each column represents a protein and the colour in each slot indicates its corresponding abundance. Note that the LYN-A*^{YF32} sample is more similar to LYN-A* than LYN-B*.

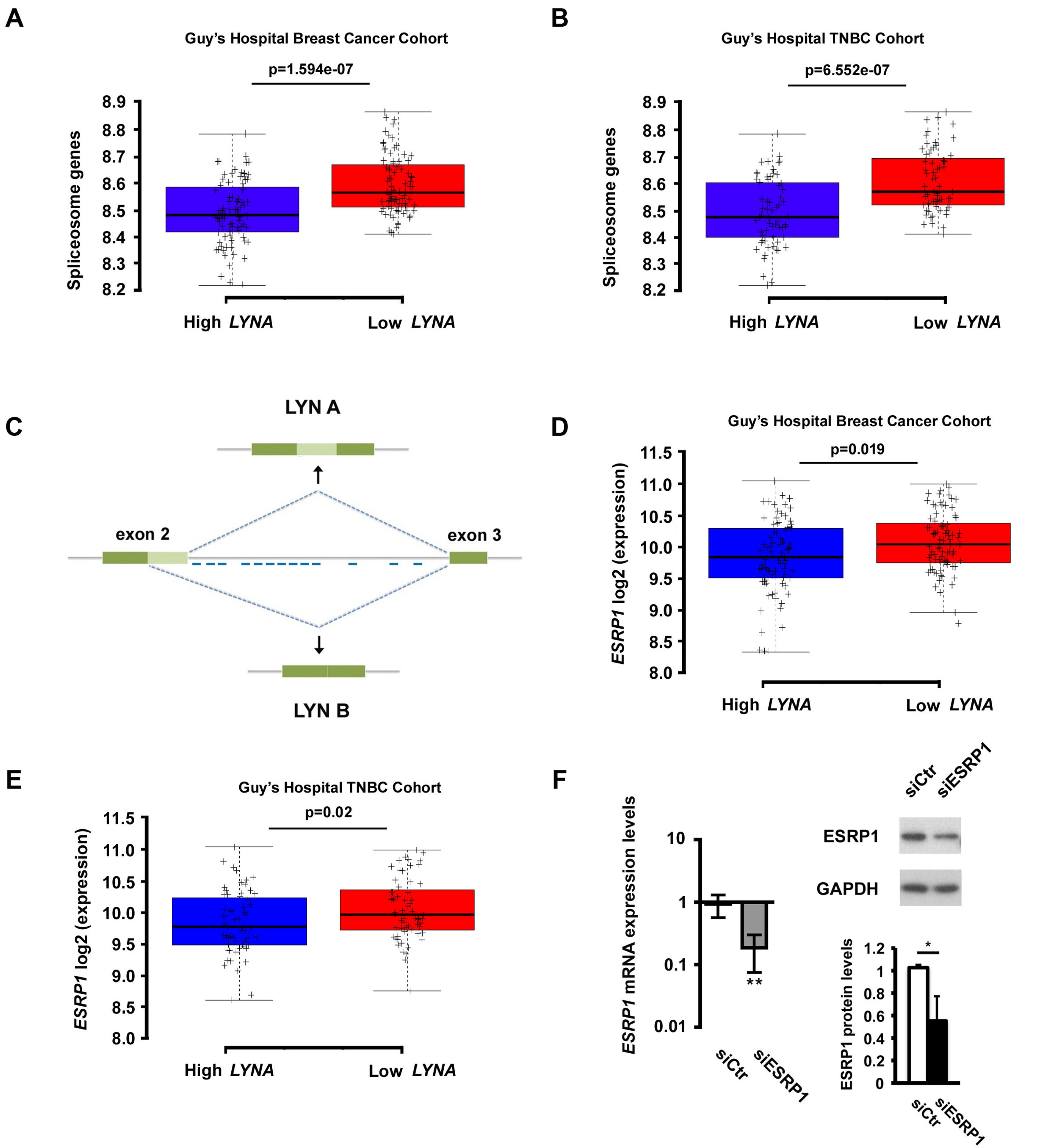

A
Guy's Hospital Breast Cancer Cohort
p=1.594e-07
Spliceosome genes
8.9
8.8
8.7
8.6
8.5
8.4
8.3
8.2
High LYNA
Low LYNA
B
Guy's Hospital TNBC Cohort
p=6.552e-07
Spliceosome genes
High LYNA
Low LYNA
C
LYN A
exon 2
exon 3
LYN B
D
Guy's Hospital Breast Cancer Cohort
p=0.019
ESRP1 log2 (expression)
11.5
11.0
10.5
10.0
9.5
9.0
8.5
8.0
High LYNA
Low LYNA
E
Guy's Hospital TNBC Cohort
p=0.02
ESRP1 log2 (expression)
High LYNA
Low LYNA
F
ESRP1 mRNA expression levels
10
1
0.1
0.01
**
siCtr
siESRP1
ESRP1
GAPDH
ESRP1 protein levels
1.2
1
0.8
0.6
0.4
0.2
0
*

Figure S8. LYN splicing is regulated by ESRP1 (related to Figure 6). (A, B) Boxplots showing the expression of a set of genes involved in splicing regulation (spliceosome) across 176 breast cancers **(A)** or 126 TNBCs divided based on *LYN A* transcript expression **(B)**. The significance of expression level differences between the two subgroups were evaluated with two-sided Student's t-tests. **(C)** Schematic of *LYN* transcript alternative splicing. Use of an alternative 5' (donor) splicing site within exon 2 results in the generation of LYN B transcript. UGG-rich motifs, putative binding elements for ESRP1 (Warzecha et al., 2010), are indicated by blue bars. **(D, E)** Boxplots showing *ESRP1* expression across 176 breast cancers **(D)** or 126 TNBC **(E)** divided based on *LYN A* transcript expression. The significant expression level differences between the two subgroups were evaluated with two-sided Student's t-tests. **(F)** MCF7 cells were transfected with control (siCtr) or *ESRP1* (siESRP1) siRNA. 72 hrs after transfection, *ESRP1* knock-down was assessed by qrtPCR (left; fold expression over comparator siCtr cells; mean±95% confidence intervals; n=3 independent experiments each of 3 technical replicates per sample) and western blot (right; mean+SD; n=3 independent experiments; two-tailed unpaired t test; **P<0.01).

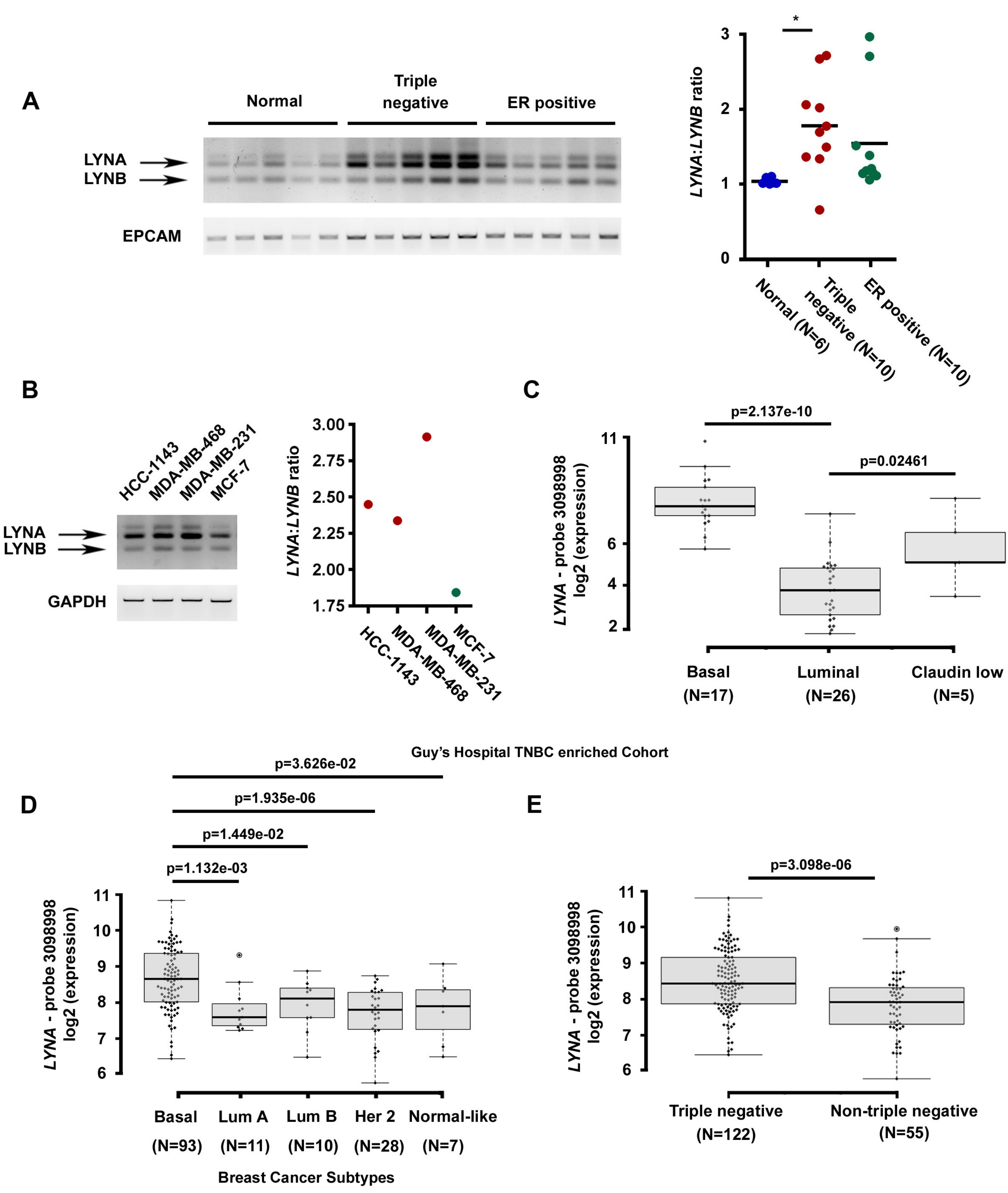
A
Normal
Triple negative
ER positive
LYNA
LYNB
EPCAM
LYNA:LYNB ratio
*
Normal (N=6)
Triple negative (N=10)
ER positive (N=10)
B
HCC-1143
MDA-MB-468
MDA-MB-231
MCF-7
LYNA
LYNB
GAPDH
LYNA:LYNB ratio
C
p=2.137e-10
p=0.02461
LYNA - probe 3098998 log2 (expression)
Basal (N=17)
Luminal (N=26)
Claudin low (N=5)
Guy's Hospital TNBC enriched Cohort
D
p=3.626e-02
p=1.935e-06
p=1.449e-02
p=1.132e-03
LYNA - probe 3098998 log2 (expression)
Basal (N=93)
Lum A (N=11)
Lum B (N=10)
Her 2 (N=28)
Normal-like (N=7)
Breast Cancer Subtypes
E
p=3.098e-06
LYNA - probe 3098998 log2 (expression)
Triple negative (N=122)
Non-triple negative (N=55)

Figure S9. LYNA expression in TN/BLBC is significantly higher than in other breast cancer subtypes (related to Figure 7). (A) Analysis of *LYNA* and *LYNB* expression in human breast tissue by RT-PCR. Arrows indicate PCR products of the expected size using primers spanning the *LYN* splicing insert. *EPCAM*, an epithelial marker, was used for normalizing to control for possible differences in amount of epithelium in tissue samples. Left panel, gels showing representative PCR results. Note the band above the *LYNA* band results from hetero-hybridization of *LYNA* and *LYNB* PCR products. Right panel, quantitation of *LYNA*:*LYNB* ratio (normal tissue from reduction mammoplasty, blue; TNBC, red; ER-positive breast cancer, green)**.** Bars indicate mean values. *$P<0.05$, unpaired two-tailed t-test. **(B)** RT-PCR analysis and quantitation of *LYNA*, *LYNB* and *GAPDH* expression in a panel of human breast cancer cell lines. MCF-7, ER-positive breast cancer cell line; MDA-MB-231, MDA-MB-468, HCC1143, TN breast cancer cell line. **(C, D, E)** Boxplots showing the *LYN A* isoform expression across breast cancer cell lines divided based on their subtypes **(C)**, across 177 breast cancers classified based on their PAM50 breast cancer subtypes **(D)** or their immune-histochemical defined subtypes **(E)**. The significant expression level differences between the different subgroups were evaluated with two-tailed t-tests.

P4 = Luminal progenitors; P5 = Luminal differentiated; P6 = stroma; P7 = basal/stem

BP213

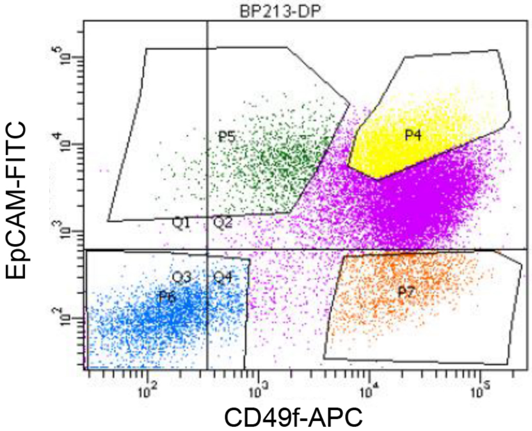

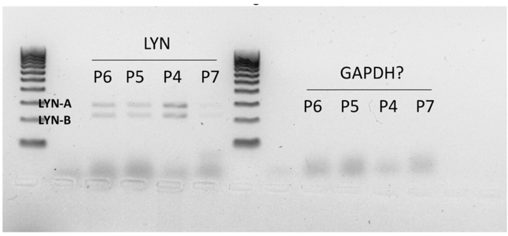

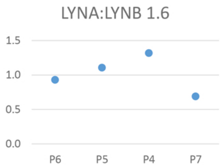

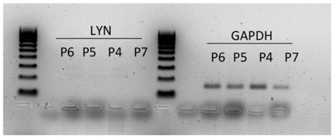

Comment: GAPDH failed in first PCR analysis; LYN very weak in second analysis

BP228

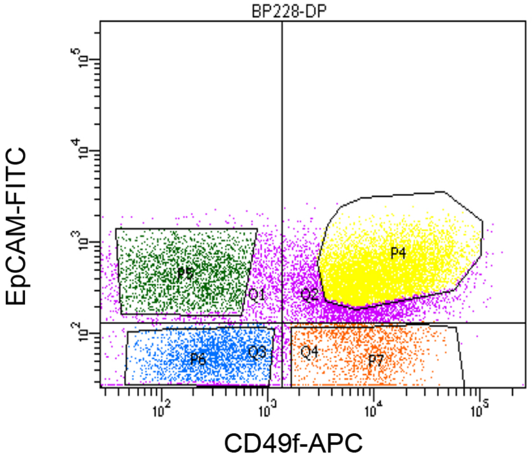

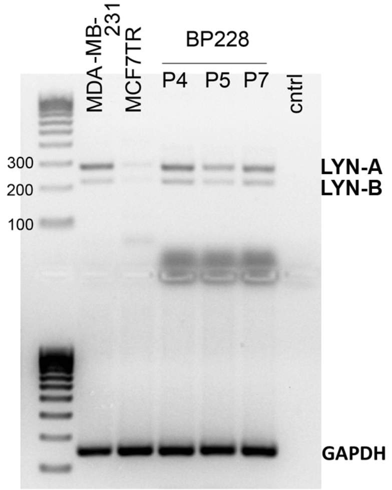

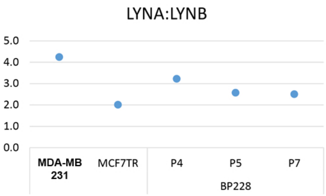

Comment: P6 stroma material insufficient for analysis

BP236

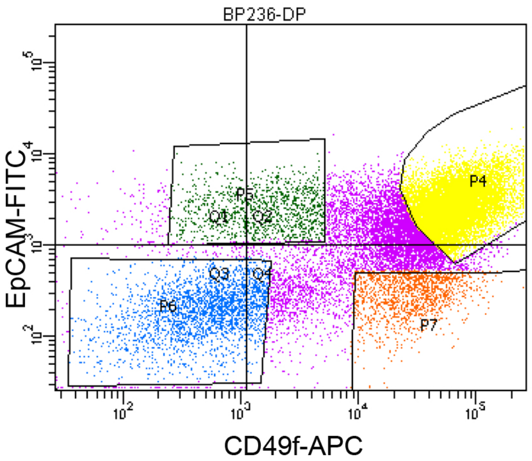

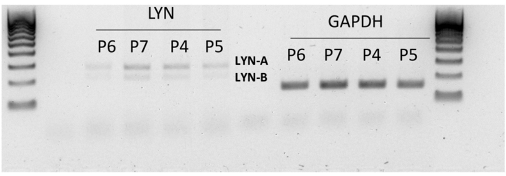

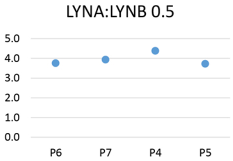

BP257

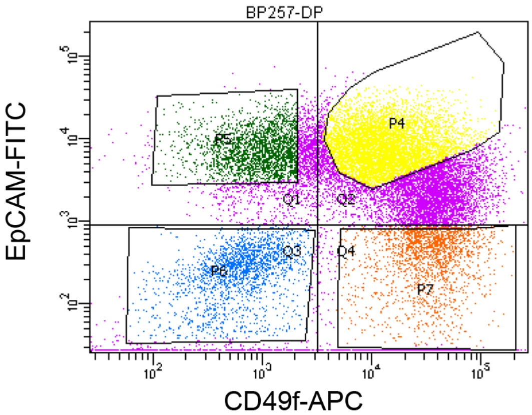

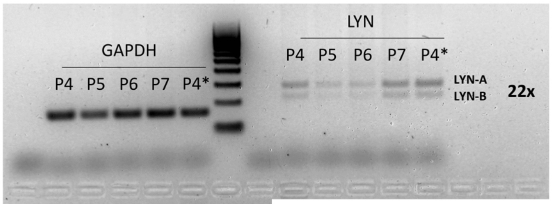

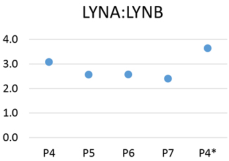

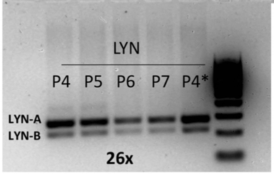

Comment: Analysis done with both 22 and 26 cycles; P4 populations collected twice with two different gates drawn, P4 and P4*. Plot shows P4*.

Figure S10. Isolation of primary normal human breast epithelial cells from reduction mammoplasty tissue (related to Figure 7). EpCAM-FITC and CD49f-APC dot plots and semi-quantitative rtPCR analysis results of *LYN* isoform expression from four independent breast preparations. Identity of the gated populations is indicated and based upon previous studies.
